# Total synthesis of avenaol

**DOI:** 10.1038/s41467-017-00792-1

**Published:** 2017-09-22

**Authors:** Motohiro Yasui, Rina Ota, Chihiro Tsukano, Yoshiji Takemoto

**Affiliations:** Graduate School of Pharmaceutical Sciences, Kyoto University, Yoshida, Sakyo-ku, Kyoto, 606-8501 Japan

## Abstract

Avenaol, isolated from the allelopathic plant black oat, was the first C_20_ germination stimulant related to strigolactones. Structurally, it consisted of a bicyclo[4.1.0]heptanone skeleton containing a cyclopropane ring bearing three main chains projecting in the same direction (i.e. all*-cis-*substituted cyclopropane). Herein, we report the total synthesis of avenaol using a robust strategy involving the formation of an all-*cis*-substituted cyclopropane via an alkylidenecyclopropane. The key factors in the success of this total synthesis include the Rh-catalysed intramolecular cyclopropanation of an allene, an Ir-catalysed stereoselective double-bond isomerisation, and the differentiation of two hydroxymethyl groups via the regioselective formation and oxidation of a tetrahydropyran based on the reactivity of a cyclopropyl group. This strategy effectively avoids the undesired ring opening of the cyclopropane ring and the formation of a caged structure. Furthermore, this study confirms the proposed structure of avenaol, including its unique all-*cis*-substituted cyclopropane moiety.

## Abstract (web summary)

Avenaol is a potent germination stimulant that can be extracted from black oat. Here, the authors report the total synthesis of avenaol by developing a strategy to access all-*cis*-substituted cyclopropanes.

## Introduction

**Fig. 1:**
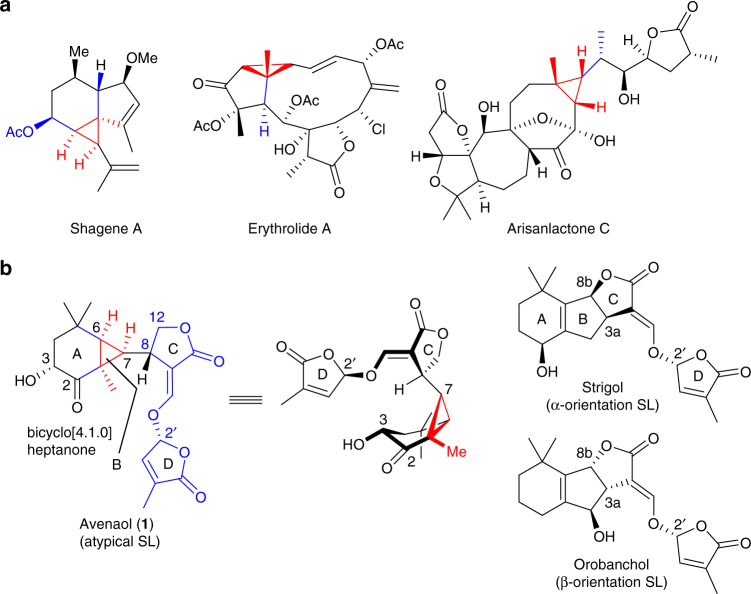
Natural products containing all-*cis*-substituted cyclopropanes. **a** Various natural products containing an all-*cis*-substituted cyclopropane, all of which have been reported to show intriguing biological activities. **b** The structures of avenaol and two typical strigolactones. The major differences between these compounds include the AB ring carbon skeleton and the connectivity between the BC rings. The construction of the AB ring of avenaol represents a challenging problem

Natural products containing complex three-dimensional structures represent challenging synthetic targets, and compounds of this type have consequently received considerable interest from research groups all over the world^1–3^. Notably, cage-shaped natural products have been reported to show several interesting biological activities, and numerous polycyclic terpene^4–7^ and alkaloid^8^ targets of this type have inspired synthetic chemists to develop innovative new strategies to access these compounds. Non-cage-shaped natural products that are capable of being converted to cage-shaped materials also exhibit an interesting range of biological activities, as exemplified by avenaol^9^, shagene A^10^, erythrolide A^11^ and arisanlactone C^12^ (Fig. 1a). These compounds are characterised by their bicyclo[4.1.0]heptane (avenaol, shagene A), bicyclo[3.1.0]hexane (erythrolide A), and bicyclo[6.1.0]nonane (arisanlactone C) skeletons, all of which contain a cyclopropane ring bearing three main chains projecting in the same direction (i.e. an all*-cis-*substituted cyclopropane). Although the synthesis of these natural products is considered to be as challenging as the synthesis of their cage-shaped counterparts, there have been very few reports to date pertaining to the synthesis of all*-cis-*substituted cyclopropanes. In fact, there have been no reports to date concerning the total synthesis of non-cage-shaped natural products containing an all-*cis*-substituted cyclopropane. Furthermore, it is envisaged that these syntheses would lead to the identification of several stereochemically interesting structures. With this in mind, we became interested in investigating the synthesis of natural products containing an all-*cis*-substituted cyclopropane using the non-typical strigolactone avenaol, which shows important biological activity, as a representative example.

Avenaol (**1**), which was first isolated from the allelopathic plant black oat (*Avena strigosa* Schreb.) by Yoneyama and co-workers in 2014, was the first reported natural C_20_ germination stimulant structurally related to strigolactones (SLs)^9^. Extensive NMR and high-resolution mass spectrometry analyses indicated that the structure of **1** consisted of a bicyclo[4.1.0]heptanone skeleton, a C ring lactone, four contiguous stereogenic centres and an all*-cis-*substituted cyclopropane. Although the all*-cis-*substituted cyclopropane ring in **1** might readily cyclise to form a caged structure via the formation of a C–C bond between the A and C rings, the analytic data for this compound were consistent with a non-cage structure (Fig. 1b). Given that the structure of avenaol differs from those of typical SLs such as strigol and orobanchol, it was designated as a novel class of SL. Avenaol shows potent germination-stimulating activity for *Phelipanche ramosa* seeds, but much lower activities for *Striga hermonthica* and *Orobanche minor*. Although the biosynthetic pathways and target protein of a few typical SLs have recently been reported^13, 14^, the biological properties of compound **1** have been poorly explored. Further study is therefore required to understand the relationship between the properties of avenaol and those of a few typical SLs. Although a variety of different strategies have been reported for the synthesis of typical SLs^15–25^, there have been no reports to date for the synthesis of avenaol.

Herein, we report the total synthesis of avenaol based on a strategy for the construction of all-*cis-*substituted cyclopropanes using alkylidenecyclopropane as a key intermediate. The core structure is constructed through the Rh-catalysed intramolecular cyclopropanation of an allene, and an Ir-catalysed stereoselective double-bond isomerisation. This strategy effectively avoids the undesired ring opening of the cyclopropane ring and the formation of a caged structure. Furthermore, this study confirms the proposed structure of avenaol, including its unique all-*cis*-substituted cyclopropane moiety.

## Results

### Retrosynthetic analysis

**Fig. 2:**
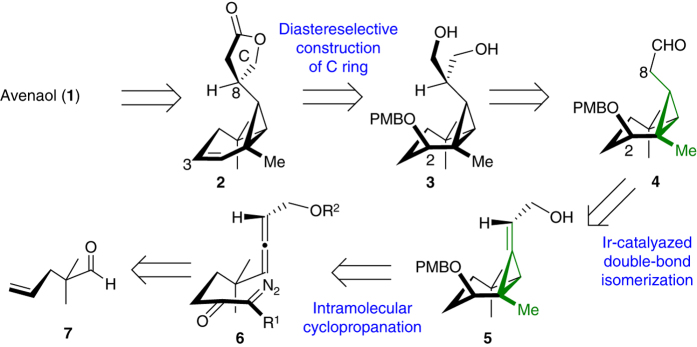
Retrosynthesis of avenaol (**1**). Our strategy is characterised by the use of an alkylidenecyclopropane intermediate **5**, providing a robust route to the required all-*cis*-substituted cyclopropane, while avoiding the undesired ring opening of the cyclopropane ring and the formation of a caged structure. The numbering of the carbon atoms is consistent with that used for avenaol. PMB *p*-methoxybenzyl

The main challenges associated with the synthesis of avenaol include the construction of a bicyclo[4.1.0]heptanone skeleton containing an all-*cis*-substituted cyclopropane, controlling the stereochemistry at the C8 position of the C ring, and the introduction of a C3 hydroxyl group on the A ring. The construction of bicyclo[4.1.0]heptanone skeletons has mainly been investigated in the context of constructing caged structures^26–28^. For non-caged structure, the direct synthesis of these systems has been limited to the 1,4-addition of a suitable anion of a *trans*-chloroallylphosphonamide^29^ or Ir-catalysed or Rh-catalysed *cis*-selective cyclopropanation reactions^30, 31^. However, preliminary work in our own group has shown that these methods are unsuitable for the synthesis of avenaol (Supplementary Fig. 1). Furthermore, cyclopropane rings bearing an electron-withdrawing group can readily undergo a ring-opening reaction^32–34^, further highlighting the difficulties of this approach. On the basis of these issues, we envisioned that the use of alkylidenecyclopropane^35^ as an appropriate intermediate would avoid an unwanted ring-opening reaction and the formation of a caged structure. We also envisioned that avenaol could be obtained from **2** by the dihydroxylation of its convex face and the introduction of the D ring (Fig. 2). The C ring lactone could be constructed by the diastereoselective transformation of the diol based on the reactivity of the cyclopropyl group in **3**, which could be obtained by introduction of a hydroxymethyl group to the all-*cis-*substituted cyclopropane **4**. Compound **4** could be synthesised by the intramolecular cyclopropanation of allene **6**, which could be readily prepared from aldehyde **7**, followed by double-bond isomerisation of alkylidenecyclopropane **5**. The intramolecular cyclopropanation of an allene to form a six-membered carbocycle has not been reported, indicating that development work would be required to allow for the construction of the bicyclo[4.1.0]heptanone core.

### Construction of an all-*cis*-substituted cyclopropane

**Fig. 3:**
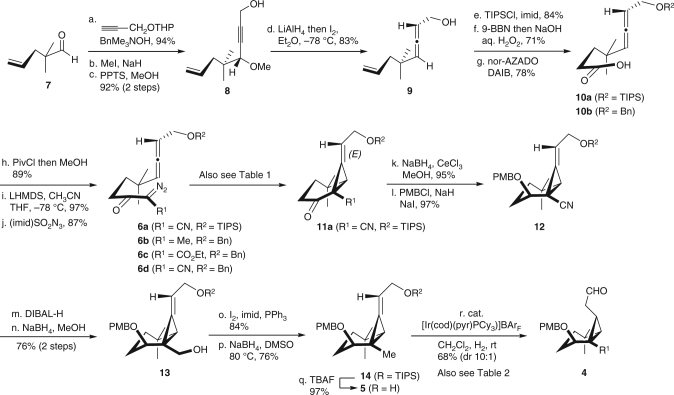
Synthesis of all-*cis*-substituted cyclopropane **4**. Conditions: a HC≡CCH_2_OTHP, BnMe_3_NOH, DMSO, 94%; b NaH, MeI, THF–DMPU; c pyridinium *p*-toluenesulfonate, MeOH, 92% (two steps); d LiAlH_4_ then I_2_, Et_2_O, −78 °C, 83%; e TIPSCl, imidazole, DMF, 84%; f 9-BBN, then aq. NaOH, aq. H_2_O_2_, 71%; g nor-AZADO, DAIB, CH_2_Cl_2_–pH 7 buffer, 78%; h PivCl, *i*Pr_2_NEt, then MeOH, DMAP, 89%; i lithium bis(trimethylsilyl)amide, CH_3_CN, THF, −78 °C, 97%; j (imidazoyl)SO_2_N_3_, pyridine, CH_3_CN, 87%; k NaBH_4_, CeCl_3_, MeOH 95%, (dr 17:1); l PMBCl, NaH, NaI, THF, 0–50 °C, 97%; m diisobutylaluminium hydride, toluene–THF, −78 °C to rt; n NaBH_4_, MeOH, 0 °C, 76% (two steps); o I_2_, PPh_3_, imidazole, CH_2_Cl_2_, 0 °C; 84%, p NaBH_4_, DMSO, 80 °C, 76%; q TBAF, THF, 97%; r [Ir(cod)(pyr)PCy_3_]BAr_F_, CH_2_Cl_2_, H_2_, 68% (dr 10:1). THP tetrahydropyranyl, DMPU *N*,*N*′-dimethylpropylene urea, triisopropylsilyl, DAIB (diacetoxyiodo)benzene, DMAP 4-dimethylaminopyridine, *BAr*_*F*_ (3,5-bisCF_3_C_6_H_3_)_4_B^−^

Our synthesis began with the preparation of the cyclisation precursor **6** (Fig. 3). The treatment of known aldehyde **7** ^36^ with 2-(prop-2-yn-1-yloxy)tetrahydro-*2H*-pyran and BnMe_3_NOH^37^, followed by methylation and acidic treatment gave **8**. The subsequent hydroalumination of **8**, followed by the treatment of the resulting intermediate with iodine gave **9** ^38^, which was converted to carboxylic acid **10a** via sequential protection as a triisopropylsilyl (TIPS) ether, hydroboration and oxidation by 9-azanoradamantane *N*-oxyl (nor-AZADO)^39^. The cyclisation precursor α-diazo-β-ketonitrile **6a** was synthesised by sequential β-ketonitrile formation and diazo transfer reactions^40^. A similar sequence was used to prepare the benzyl-protected methyl diazoketone **6b**. The α-diazo-β-ketoester **6c** and ketonitrile **6d** were also synthesised via **10b** (Supplementary Fig. 2).

We subsequently investigated the formation of the alkylidenecyclopropane intermediates via the intramolecular cyclopropanation of allenes **6a**–**d** using a Rh or Cu catalyst^41^. We initially investigated the cyclisation of methyl diazoketone **6b** with Rh_2_(OAc)_4_, but this reaction failed to afford the desired cyclised product (Table 1, entry 1). Substrates bearing a β-ketoester or ketonitrile instead of a methyl group were also evaluated in an attempt to stabilise the metal carbene. The reaction of **6c** with Rh_2_(OAc)_4_ or Cu(CH_3_CN)_4_PF_6_ did not give the desired product (Table 1, entries 2 and 3). In sharp contrast, the cyclisation reactions of the α-diazo-β-ketonitriles **6a** and **6d** with Rh_2_(OAc)_4_ proceeded smoothly to give the alkylidenecyclopropanes **11a** and **11d**, respectively, in excellent yields, most likely because of the more electrophilic nature of the metal carbene (Table 1, entries 4 and 5)^42^. It is noteworthy that this reaction only afforded the *E* isomer, because the metal carbene only approached from the less hindered face of the allene. For further transformation toward the all-*cis*-substituted cyclopropane, we used compound **11a** because of the ease with which this compound could undergo protecting group manipulation.

**Table 1:** Formation of alkylidenecyclopropane by allene cyclopropanation


Entry	Substrate	R^1^	R^2^	Catalyst	Yield^a^
1	**6b**	Me	Bn	Rh_2_(OAc)_4_	0%^b^
2	**6c**	CO_2_Et	Bn	Rh_2_(OAc)_4_	0%^c^
3	**6c**	CO_2_Et	Bn	Cu(CH_3_CN)_4_PF_6_	0%^d^
4	**6d**	CN	Bn	Rh_2_(OAc)_4_	85%
5	**6a**	CN	TIPS	Rh_2_(OAc)_4_	84%

^a^Isolated yield

^b^Carboxylic acid **10b** was obtained in 26% yield

^c^Carboxylic acid **10b** was obtained in 35% yield

^d^The reaction gave a complex mixture

**Fig. 4:**
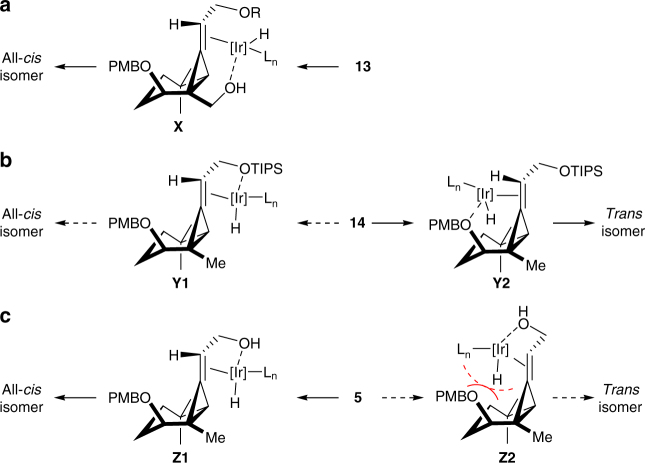
Stereoselectivity of Ir-catalysed double-bond isomerisation. The stereoselectivity of the double-bond isomerisation could be controlled by the directing group. **a** The Ir catalyst coordinated preferentially to the alcohol moiety in **13**, resulting in the exclusive formation of the all-*cis* isomer. **b** Coordination to the TIPS ether was found to be less favourable than the PMB ether, resulting in the formation of the *trans* isomer through intermediated **Y2**. **c** After the coordination of the alcohol to the catalyst, the reaction proceeded through intermediate **Z1**, which was favoured over **Z2** because of steric repulsion

Next, we focused on the construction of the all-*cis*-substituted cyclopropane structure from alkylidenecyclopropane **11a**. We initially investigated the hydrogenation of the alkylidenecyclopropanes (Supplementary Fig. 3). For example, compound **12**, which was prepared by reduction of **11a**, followed by the PMB protection of the resulting alcohol, was hydrogenated over a Pd on carbon catalyst using H_2_ gas. Surprisingly, this reaction gave the undesired *trans* isomer as the major product, most likely because of the steric effect of the nitrile group. We subsequently investigated the transition-metal-catalysed isomerisation of the double bond in this system using a directing group to reverse this selectivity. To determine the best position for the directing groups, we prepared alcohol **13**, silyl ether **14** and allyl alcohol **5** by the stepwise reduction of the nitrile group (Fig. 3). Despite our initial concerns regarding the ring-opening of the cyclopropane system during these transformations, the cyclopropane ring remained intact because it was stabilised as an alkylidenecyclopropane. Compound **12** was initially treated with Crabtree’s catalyst^43^, which was preactivated with H_2_, but failed to afford the all-*cis*-substituted cyclopropane (Table 2, entry 1). Use of a substrate having nitrile and hydroxyl groups resulted in no reaction, and recovery of the starting material (Supplementary Fig. 4). These results indicated that the nitrile group deactivated the Ir catalyst rather than acting as a directing group. In contrast, the reaction of **13** bearing a hydroxymethyl group under these conditions, allowed for the successful isomerisation of the olefin under H_2_ to give the silyl enol ether **15a** in 92% yield with excellent stereoselectivity (Table 2, entry 2). The success of this reaction indicated that the Ir catalyst approached from one face after its coordination to the alcohol (i.e. **X**, Fig. 4a), resulting in the exclusive formation of the all-*cis* isomer. However, the hydroxymethyl group on **15a** could not be converted to a methyl group without opening the cyclopropane ring. Notably, the treatment of silyl ether **14** with Crabtree’s catalyst resulted in very little reaction, because of the lack of a directing group of the substrate and relatively low reactivity of the catalyst (Table 2, entry 3). The reactivity improved when we used Pfaltz’s modified Ir catalyst bearing a non-coordinating counter anion (i.e. BAr_F_)^44–46^, although this catalyst only afforded the *trans* isomer (Table 2, entry 4). The selectivity of this reaction was attributed to intermediate **Y2**, where the PMB ether would act as a better directing group rather than the corresponding TIPS ether (Fig. 4b). Allyl alcohol **5** was therefore used for this conversion. The reaction of **5** under the same conditions^44, 45^ gave aldehyde **4** and alcohol **16b** as 2.7:1 isomeric mixtures, respectively (Table 2, entry 5). This selectivity can be explained by the preferential formation of reaction intermediate **Z1** over **Z2**, which would suffer considerable steric hindrance (Fig. 4c). The diastereomeric ratio improved considerably when we used Pfaltz’s modified Ir catalyst to promote the coordination of the substrate to the catalyst, affording an all-*cis*:*trans* ratio of 10:1 (Table 2, entry 6). Most notably, the selectivity of this step was found to be highly reproducible, allowing us to generate gram-scale quantities of the all-*cis*-substituted cyclopropane **4** for further transformations.

**Table 2:** Formation of all-*cis*-substituted cyclopropane


Entry	Substrate	R^1^	R^2^	X	Yield (all-*cis*:*trans*)^a,b^
1	**12**	CN	TIPS	PF_6_	No reaction
2	**13**	CH_2_OH	TIPS	PF_6_	**15a**: 92% (all-*cis* only)
3	**14**	Me	TIPS	PF_6_	**15b**: 6% (2.3:1)^c^
4	**14**	Me	TIPS	BAr_F_	**15b**: 17% (*trans* only)
					**16a**: 75% (*trans* only)
5	**5**	Me	H	PF_6_	**4**: 61% (2.7:1)
					**16b**: 5% (2.7:1)
6	**5**	Me	H	BAr_F_	**4**: 68% (10:1)
					**16b**: <5% (2.6:1)

BAr_F_, (3,5-bisCF_3_C_6_H_3_)_4_B^−^, cod, cyclooctadiene, pyr, pyridine, Cy, cyclohexyl

^a^Isolated yield

^b^The ratio was estimated using ^1^H NMR spectroscopy

^c^Starting material **14** was recovered (64%)

### Total synthesis of avenaol

**Fig. 5:**
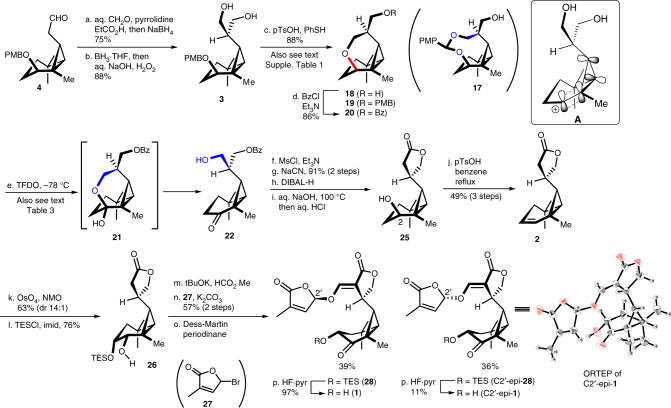
Total synthesis of avenaol (**1**). Conditions: a aq. CH_2_O, pyrrolidine, EtCO_2_H, *i*PrOH, 45 °C, then NaBH_4_, MeOH, 0 °C, 75%; b BH_3_·THF, then aq. NaOH, aq. H_2_O_2_, 88%; c *p-*toluenesulfonic acid, PhSH, CH_2_Cl_2_, 88%; d BzCl, Et_3_N, DMAP, CH_2_Cl_2_, 86%; e TFDO, CH_2_Cl_2_, −78 °C, f mesyl chloride, Et_3_N, CH_2_Cl_2_; g NaCN, 15-crown-5, DMSO, 91% (two steps); h diisobutylaluminium hydride, THF, −20 °C; i aq. NaOH, 100 °C, then 1 M aq. HCl; j *p-*toluenesulfonic acid, benzene, reflux, 49% (three steps); k OsO_4_, 4-methylmorpholine *N*-oxide, acetone–*t*BuOH–H_2_O, 63% (dr 14:1); l TESCl, imidazole, DMF, 76%; m *t*BuOK, HCO_2_Me, THF; n 5-bromo-3-methylfuran-2-one, K_2_CO_3_, 1-methyl-2-pyrrolidinone, 57% (two steps), dr 1:1; o Dess–Martin periodinane, CH_2_Cl_2_–pyr, 39% (for **28**) and 36% for (C2′-epi-**28**); p HF·pyr, THF, 97% (for **1**), 11% (for C2′ epi-**1**). Bz Benzoyl, TES triethylsilyl, TFDO methyl(trifluromethyl)dioxirane

Having established a successful route to the all-*cis*-substituted cyclopropane, we turned our attention to the formation of the C ring. Aldehyde **4** was converted to diol **3** by the introduction of an *exo* methylene at the α position of the aldehyde, followed by a hydroboration (Fig. 5). We subsequently screened a wide range of conditions to allow for the differentiation of the hydroxymethyl group. Surprisingly, the diastereoselective DDQ-mediated intramolecular cyclisation of **3** to give acetal **17** resulted in the formation of the caged compound **18** in ~30% yield as a single isomer (Supplementary Table 1, entry 1). We envisaged that the selective transformation of **18** to **22** via an oxidative ring cleavage reaction would allow for the two hydroxymethyl groups to be differentiated. Thus, we switched our focus to the unexpected formation of the tetrahydropyran ring. The treatment of this system with Cu(OTf)_2_ was found to be ineffective, whilst Zn(OTf)_2_ and Sc(OTf)_3_ gave the cyclised products **18** and **19** (Supplementary Table 1, entries 2–4). These results indicated that acidic conditions would be important, and that this transformation would proceed via the secondary cation intermediate **A**. With this in mind, we investigated the addition of BF_3_·OEt_2_ and *p*-toluenesulfonic acid (pTsOH) (Supplementary Table 1, entries 6 and 7). The results revealed that pTsOH gave a best yield, although a large portion of the other hydroxy group also reacted with a by-product derived from PMB group to give mixture of **18** and **19**. The addition of thiophenol (PhSH) was effective for trapping this by-product, allowing for the diastereoselective formation of the desired product **18** as a single product. Interestingly, the ring-opening product was not observed under these conditions, most likely because of the stability of the bisected cyclopropylcarbinyl cation intermediate **A**, where the π-orbitals of the cation would interact with the *sp* ^2^-like orbitals of the cyclopropane ring^47–49^.

The C–H oxidation of the tetrahydropyran ring in **18** was investigated for the construction of compound **22**. The alcohol moiety in compound **18** was initially protected by a benzoyl group. The addition of a stoichiometric charge of CrO_3_ or a combination of RuCl_3_ and NaIO_4_ resulted in the oxidation of the ring to give the undesired lactone **23** and carboxylic acid **24**, respectively (Table 3, entries 1 and 2). We also conducted the C–H oxidation according to the procedure reported by White’s group using (2*S*,2′*S*-(−)-[*N*,*N*′-bis(2-pyridylmethyl)]-2,2′-bipyrrolidinebis(acetonitrile)iron(II) hexafluoroantimonate ((*S*,*S*)-Fe(pdp))^50^, which gave the desired alcohol **22** in 65% yield via **21**. It is noteworthy, however, that this reaction required a stoichiometric amount of an iron reagent (Table 3, entry 3). In an attempt to improve the yield, we investigated the use of dimethyldioxirane (DMDO) and methyltrifluromethyldioxirane (TFDO)^51^. The use of an excess of DMDO resulted in a slow reaction (i.e. starting material remaining after 1 day) (Table 3, entry 4), whereas the use of TFDO at 0 °C gave a mixture of alcohol **22** and lactone **23**, presumably because of its high reactivity (Table 3, entry 5). This reaction was subsequently performed at −78 °C to improve the regioselectivity and proceeded smoothly to give **22** in excellent yield (Table 3, entry 6).

**Table 3:** C–H oxidation of tetrahydrofuran 20


Entry	Conditions	Yield of **22** ^a^
1	CrO_3_, AcOH, rt	0% (**23**: 35%)
2	RuCl_3_, NaIO_4_,CCl_4_–MeCN–pH7 buffer, rt	0% (**23**: 7%, **24**: 33%)
3	(*S*,*S*)-Fe(pdp), AcOH, aq. H_2_O_2_, rt	65%
4	DMDO, CH_2_Cl_2_, rt	22% (30% brsm)
5	TFDO, CH_2_Cl_2_, 0 °C	24% (**23**: 36%)
6	TFDO, CH_2_Cl_2_, −78 °C	96%

DMDO, dimethyldioxirane, pdp, *N,N*’-bis(2-pyridylmethyl)]-2,2′-bipyrrolidine, TFDO, methyl(trifluoromethyl)dioxirane

^a^Isolated yield

The final stage of the total synthesis involved the formation of an α-hydroxyketone and the introduction of the D ring of avenaol. Mesylation of the alcohol moiety in **22**, followed by the substitution of the resulting mesylate with cyanide gave the corresponding nitrile (Fig. 5). The subsequent reduction of the ketone moiety, hydrolysis of the nitrile and benzoyl protecting groups, followed by an acidic treatment, resulted in the formation of the lactone ring to give **25**. To avoid the possibility of an intramolecular cyclisation between the α position of the lactone and the C2 position of the A ring (Supplementary Fig. 5), we proceeded via the dehydration of the alcohol rather than an oxidative transformation. The stereoselective dihydroxylation of **2**, followed by the selective protection of the alcohol at the C3, gave **26** with excellent selectivity. Formylation, followed by the introduction of a butenolide unit **27** ^16^ gave a mixture of C2′ epimers. Dess–Martin oxidation gave the protected avenaol **28**, which was separated from the corresponding C2′ epi-**28** by column chromatography over silica gel. To determine the stereochemistry, the silyl groups in both **28** and C2′ epi-**28** were subsequently removed using HF·pyridine to give avenaol (**1**) and C2′ epi-**1**, respectively. Despite the low yield for the latter of these two reactions, we were able to obtained a crystal of C2′ epi-**1** for X-ray crystallography. The X-ray crystal structure of C2′ epi-**1** confirmed that the relative stereochemistry between C2′ and C8 was as shown in Fig 5. Moreover, the spectroscopic data obtained for synthetic avenaol (**1**) (i.e. ^1^H, ^13^C NMR and HRMS) were identical to those of the natural sample **1** ^9^. These results therefore confirm that the proposed structure is correct.

## Discussion

In summary, we have achieved the total synthesis of avenaol. The key feature of this synthesis is the use of an alkylidenecyclopropane, which allowed for the robust formation of an all-*cis*-substituted structure via a stereoselective double-bond isomerisation and avoided the cleavage of the cyclopropane ring and the formation of a caged structure. The regioselective formation and C–H oxidation of a tetrahydrofuran ring were also important steps in this synthesis. The interesting structural features of avenaol, including its all-*cis*-substituted cyclopropane were confirmed to be correct as a consequence of our total synthesis. This established route has provided synthetic samples for further biological evaluation and is also suitable for the synthesis of a range of analogues. Furthermore, this strategy will provide a platform for the synthesis of other natural products containing this structure. The extension of this strategy to structurally related natural products, as well as structure–activity relationship studies of avenaol, are currently underway.

## Methods

### General

All non-aqueous reactions were run in dried glassware under a positive pressure of argon atomosphere. Reactions were monitored by thin-layer chromatography using Silica gel 60 plates (Merck, Darmstadt, Germany). Silica gel column chromatography was performed using Chromatorex BW-300 (Fuji silysia, Aichi, Japan) and Kanto silica gel 60 (particle size 63–210 μm, Kanto, Tokyo, Japan). Proton nuclear magnetic resonance (^1^H NMR) spectra were taken with a JNM-AL 400 (JEOL) at 400 MHz or a JNM-ECA 500 (JEOL, Tokyo, Japan) at 500 MHz. Chemical shifts were measured relative to the residual solvent peak in C_6_D_6_ (*δ* 7.16) or Me_4_Si (*δ* 0.00) in CDCl_3_. Multiplicity was indicated by one or more of the following: s (singlet); d (doublet); t (triplet); q (quartet); m (multiplet); br (broad). Carbon nuclear magnetic resonance (^13^C NMR) spectra were recorded on a JNM-AL 400 at 100 MHz or a JNM-ECA 500 at 126 MHz. Chemical shifts were measured relative to CDCl_3_ (*δ* 77.0) or C_6_D_6_ (*δ* 128). Infrared spectra were collected on a FT/IR-4100 Fourier-transform infrared spectrometer (JASCO, Tokyo, Japan) as ATR (attenuated total reflectance). Low and high-resolution mass spectra were recorded on a LCMS-IT-TOF (Shimadzu, Kyoto, Japan) for ESI-MS and JMS-700 mass spectrometer (JEOL) for FAB-MS.

### Experimental data

For the experimental procedures and spectroscopic and physical data of the compounds and the crystallographic data of C2′-epi avenaol, see Supplementary Methods. For NMR spectra of synthetic intermediates, see Supplementary Figs. 5–35. For the comparisons of ^1^H and ^13^C NMR spectra of the natural and synthetic avenaol, see Supplementary Figs. 36 and 37.

### Data availability

The X-ray crystallographic coordinates for the structure of C2′-epi avenaol have been deposited at the Cambridge Crystallographic Data Centre (CCDC), under deposition number 1544731. These data can be obtained free of charge from The Cambridge Crystallographic Data Centre via www.ccdc.cam.ac.uk/data_request/cif. Other data are available from the authors upon reasonable request.

## Electronic supplementary material

Supplementary Information
