# Supplementary material for: Total synthesis of avenaol
Source: Nat Commun. 2017 Sep 22;8:674. doi: 10.1038/s41467-017-00792-1 (PMC5610312; doi:10.1038/s41467-017-00792-1)
Supplement: Supplementary file 1 — Supplementary Information [file 41467_2017_792_MOESM1_ESM.pdf]

SI GUIDE

File Name: Supplementary Information
Description: Supplementary Figures and Supplementary Tables.

Supplementary Figures

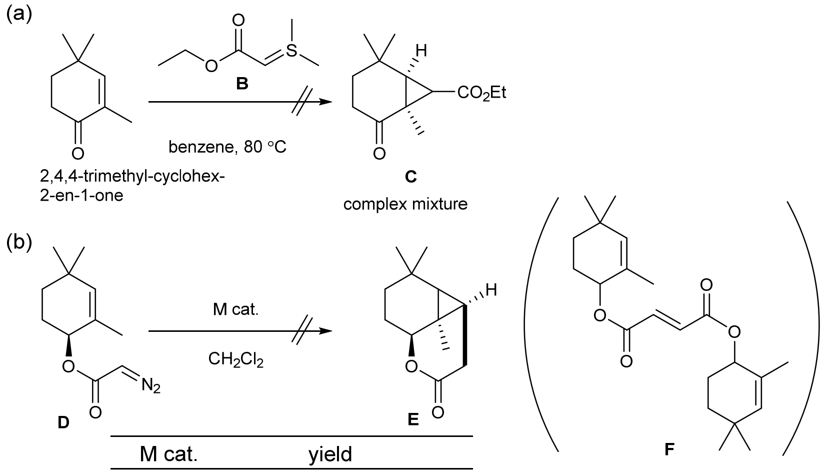

M cat.	yield
$Rh_2(cap)_4$	**E** 0% (complex mixture)
Cu(TBS)$_2$	**E** 0%, **F** 80%

Supplementary Fig. 1. Initial attempts for construction of bicyclo[4.1.0]heptanone having an all-*cis* cyclopropane. To construct an all-*cis* substituted cyclopropane, we initially attempted an intermolecular 1,4-addition of 2,4,4-trimethylcyclohex-2-en-1-one with dimethylsulfonium ylide **B**, but the reactions did not proceed at all (a). An intramolecular cyclopropanation of diazoester **D** was next attempted. A treatment of **D** with $Rh_2(cap)_2$ gave a complex mixture (b). On the other hand, use of Cu(TBS)$_2$ resulted in dimerization to give compound **F** in high yield. During these investigations, the desired products **C** and **E** were not detected at all.

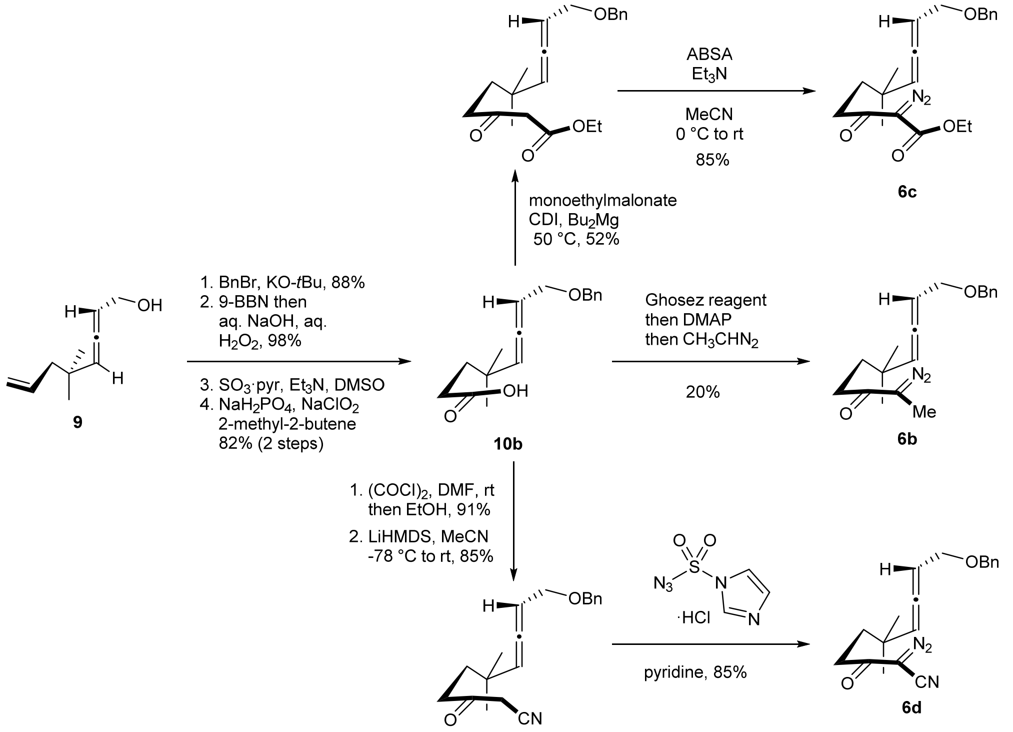

Supplementary Fig. 2. Synthesis of α-diazoketones 6b-d. To prepare benzyl-protected methyl diazoketone **6b**; α-diazo-β-ketoester **6c** and ketonitrile **6d** were also synthesized via **10b** using pproximately the same sequence for synthesis of **6a**.

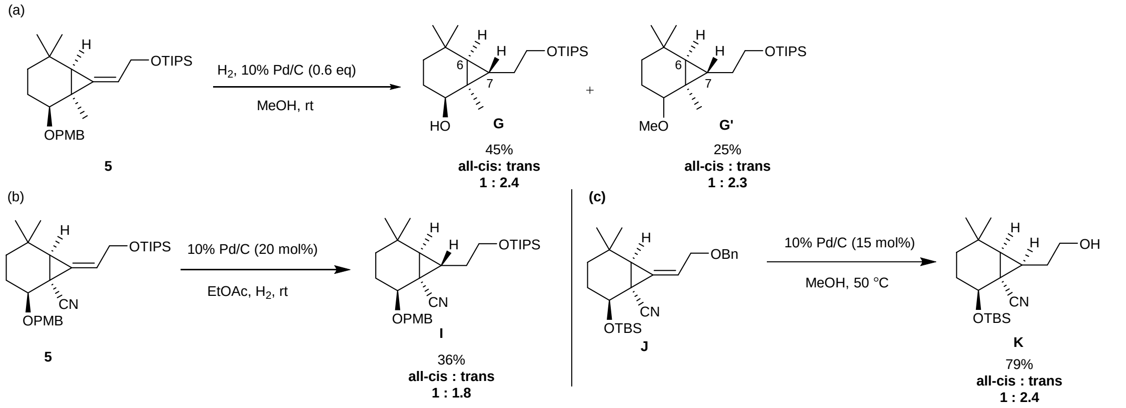

Supplementary Fig. 3. Hydrogenation of alkylidenecyclopropane. Hydrogenation of alkylidenecyclopropane **5** with Pd/C under H_2 in MeOH gave an undesired trans isomer **G** as a major product (a). We also tried hydrogenation in EtOAc or with another substrate having nitrile group. These reactions gave a mixture in which a trans substituted silyl ether **I** and alcohol **K** were major products, respectively (b and 3).

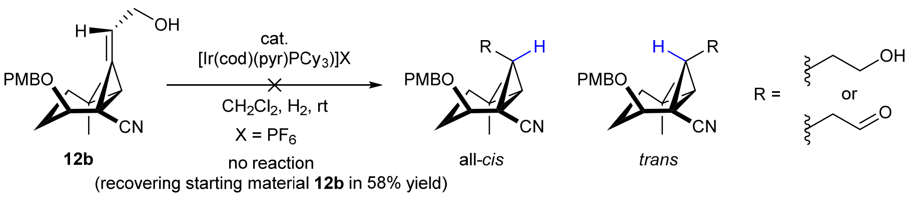

Supplementary Fig. 4. Failed attempt of formation of all-*cis*-substituted cyclopropane. Use of a substrate **12b** having nitrile and hydroxyl groups resulted in no reaction, and recovering a starting material in 58% yield.

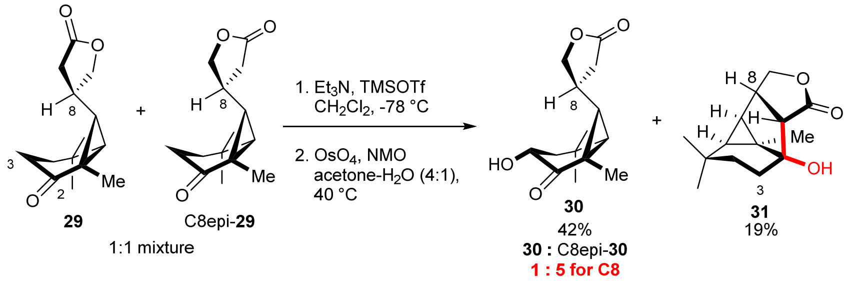

Supplementary Fig. 5. Unexpected formation of cage structure. A diastereomeric mixture of ketolactone **29** and C8-epi-**29** was converted to silyl enol ethers followed by dihydoxylation with OsO_4. While the oxidant approached from α-face to give compound **30** having desired stereochemistry, it was minor because an intramolecular aldol cyclization proceeded to give product **31** from compound **29**. These results indicated that **29** was readily to cyclize to form cage-structure under basic conditions, and while stereoselective C3 oxidation of C8-epi-**29** occurred to give an undesired isomer C8-epi-**30**.

Supplementary Table

Supplementary Table 1. Formation of tertahydrofuran.

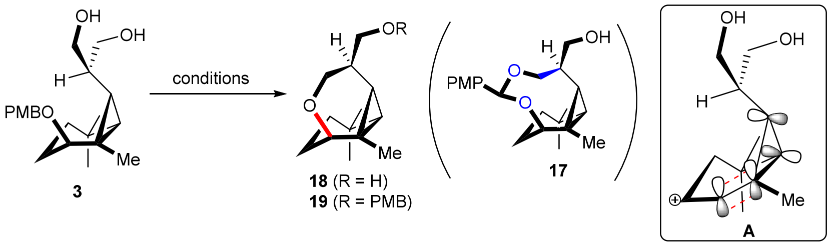

Entry	Conditions	Isolated yield
1	DDQ, MS 4A, CH_2Cl_2, 0 °C	**18**: *ca.* 30% (**17**: 0%)
2	$Cu(OTf)_2$, CH_2Cl_2, rt	**18**: trace
3	$Zn(OTf)_2$, CH_2Cl_2, rt	**18**: 14%
4	$Sc(OTf)_3$, CH_2Cl_2, rt	**18**: 27%, **19**: 36%
5	$BF_3{\cdot}OEt_2$, CH_2Cl_2, rt	**18**: 64%, **19**: 17%
6	pTsOH, CH_2Cl_2, rt	**18**: 35%, **19**: 65%
7	pTsOH, PhSH, CH_2Cl_2, rt	**18**: 88%

In the case of DDQ, the reaction gave various unidentified products, and the only isolated product was compound **18** (Supplementary Table 1, entry 1). Use of $Cu(OTf)_2$ was not effective, the reaction did not proceed, and almost all of the starting material was recovered (Supplementary Table 1, entry 2). In the case of $Zn(OTf)_2$, a significant amount of the starting material was recovered, while the reaction gave the desired product in 14% yield (Supplementary Table 1, entry 3). Through these examinations as shown in this table, a ring-opening product was not observed.

Supplementary Methods

General Information

All non-aqueous reactions were carried out under a positive atmosphere of argon in dried glassware. Analytical thin-layer chromatography was performed with Silica gel 60 (Merck). Silica gel column chromatography was performed with Kanto silica gel 60 (particle size, 63–210 μm) and Fuji silysia Chromatorex BW-300. All melting points were determined on YAMAMOTO micro melting point apparatus and are uncorrected. Proton nuclear magnetic resonance (^{1}H NMR) spectra were recorded on a JEOL JNM-ECA 500 at 500 MHz or a JEOL JNM-AL 400 at 400 MHz. Chemical shifts are reported relative to Me_4Si (δ 0.0) in $CDCl_3$, and residual solvents of C_6D_6 (δ 7.16). Multiplicity is indicated by one or more of the following: s (singlet); d (doublet); t (triplet); q (quartet); m (multiplet); br (broad). Carbon nuclear magnetic resonance (^{13}C NMR) spectra were recorded on a JEOL JNM-ECA 500 at 126 MHz or a JEOL JNM-AL 400 at 100 MHz. Chemical shifts are reported relative to $CDCl_3$ (δ 77.0), C_6D_6 (δ 128.0). Infrared spectra were recorded on a FT/IR-4100 Fourier-transform infrared spectrometer ATR (attenuated total reflectance). Low and High resolution mass spectra were recorded on JEOL MS700 mass spectrometer for FAB-MS, and Shimadzu LCMS-IT-TOF for ESI-MS. Optical rotations were determined with a JASCO P-2200KDT polarimeter and are the average of five measurements.

Synthetic procedure for total synthesis of avenaol (1).

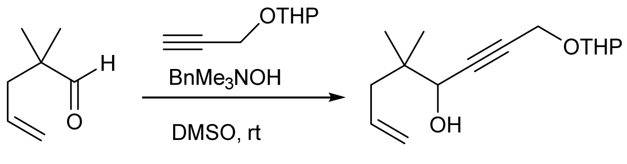

7 94% 1'

Compound 1': To a solution of 2,2-dimethyl-4-pentenal **7** (43.3 g, 386 mmol) in DMSO (193 mL) were added triton B (40% in MeOH, 14.7 ml, 41.9 mmol) and (2-propynyloxy)tetrahydropyran (49.2 g, 351 mmol). The mixture was stirred at room temperature for 24 h, and it became a dark red solution. The resulting mixture was diluted with water at 0 °C, extracted with EtOAc three times. The combined organic layers were dried over Na_2SO_4 and filtered. The filtrate was concentrated under reduced pressure, and a residue was purified by silica gel column chromatography (5-30% EtOAc/hexane) to afford **1'** (96.8 g, 94%) as a colorless oil; ^{1}H NMR (500 MHz, $CDCl_3$) δ 5.83 (m, 1H), 5.09 (d, 1H *J* = 5.5 Hz), 5.06 (s, 1H), 4.32 (dd, 2H, *J* = 15.8, 24.9 Hz), 4.13 (s, 1H), 3.84 (m, 1H), 3.55 (m, 1H), 2.21-2.07 (m, 2H), 1.87-1.71 (m, 3H), 1.65-1.52 (m, 4H), 0.98 (s, 3H), 0.97 (s, 3H); ^{13}C NMR (125 MHz, $CDCl_3$) δ 134.8, 117.7, 96.6, 85.5, 82.0, 70.1, 62.0, 62.0, 60.3, 54.1, 42.7, 38.6, 30.2, 25.3, 22.6, 22.4, 19.0, 14.1; IR (ATR) ν = 3438, 3073, 2942, 2871, 1638 cm^{-1}; HRMS (ESI) *m/z* calcd for $C_{15}H_{24}O_3Na$ [$(M+Na)^+$] 275.1618, found 275.1611.

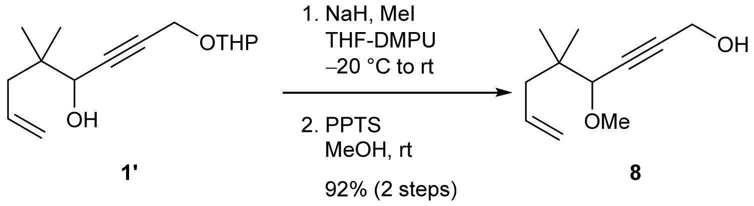

Compound 8: To a solution of compound **1’** (26.4 g, 105 mmol) in THF/DMPU (v/v = 4:1, 350 ml) was added sodium hydride (60% in mineral oil, 6.48 g, 162 mmol) at −20 °C. The mixture was stirred for 30 min. Methyl iodide (9.80 mL, 157 mmol) was added to the reaction mixture. Then, the reaction mixture was stirred at room temperature for 1 h. The resulting mixture was quenched with satd. aq. NH_4Cl and extracted with ether three times. The combined organic layers were dried over Na_2SO_4, filtered, and concentrated under reduced pressure to give a crude propargyl methyl ether (34.8 g, crude).

To a solution of the above crude propargyl methyl ether (34.8 g, crude) in MeOH (290 mL) was added PPTS (18.8 g, 74.9 mmol) at room temperature. The solution was stirred overnight. The reaction was quenched with satd. aq. $NaHCO_3$ at 0 °C. The mixture was extracted with ether three times, and the combined organic layers were washed with brine. The organic layer was dried over Na_2SO_4, filtered and concentrated under reduced pressure. The residue was purified by silica gel column chromatography (5-25% EtOAt/hexane) to give **8** (17.5 g, 92% for 2 steps) as a colorless oil: ^{1}H NMR (500 MHz, $CDCl_3$) δ 5.80 (m, 1H), 5.07 (s, 1H), 5.04 (d, *J* = 6.0 Hz), 4.34 (dd, 2H, *J* = 1.5 Hz, *J* = 6.0 Hz), 3.62 (s, 1H), 3.40 (s, 3H), 0.60 (d, 1H, *J* = 8.0 Hz), 2.14 (d, 1H, *J* = 7.5 Hz), 2.11 (d, 1H, *J* = 7.5 Hz), 2.08 (d, 1H, *J* = 8.0 Hz), 0.97 (s, 3H), 0.95 (s, 3H); ^{13}C NMR (125 MHz, $CDCl_3$) δ 134.7, 117.5, 85.0, 83.3, 79.1, 57.4, 51.2, 42.9, 38.2, 23.1, 22.7; IR (ATR) ν = 3414, 3075, 2975, 2822, 2362 cm $^{-1}$; HRMS (ESI) m/z calcd for $C_{11}H_{19}O_2$ [$(M+H)^+$] 183.1380, found *m/z* 183.1372.

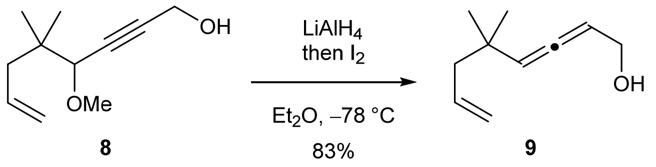

Compound 9: To a solution of $LiAlH_4$ (3.99 g, 105 mmol) in Et_2O (331 mL) was added an Et_2O (19 mL) solution of **8** (6.39 g, 35.1 mmol) dropwise through cannula at −78 °C. After the reaction mixture was stirred at room temperature for 1 h, I_2 (23.9 g, 94.2 mmol) was added slowly at −78 °C, and then stirred at room temperature for 45 min. The reaction was quenched with a mixed solution of satd. aq. Rochelle salt and 10% aq. $Na_2S_2O_3$. The resulting mixture was extracted with ether three times, dried over Na_2SO_4, filtered and concentrated under reduced pressure. The residue was purified by silica gel column chromatography (10-20% EtOAc/hexane) to afford as **9** colorless oil

(4.44 g, 83%): ^{1}H NMR (500 MHz, $CDCl_3$) δ 5.82 (m, 1H), 5.43 (dd, 2H, J = 6.0, 11.7 Hz), 5.30 (m, 1H), 5.05 (m, 2H), 4.12 (m, 2H), 0.76 (d, 2H), 1.41 (t, 1H), 1.04 (s, 3H), 1.03 (s, 3H); ^{13}C NMR (125 MHz, $CDCl_3$) δ 200.6, 135.1, 117.0, 104.1, 93.4, 60.5, 47.1, 27.6, 27.2; IR (ATR) ν = 3377, 3074, 2961, 2927, 2870, 1960 cm^{-1}; HRMS (ESI) *m/z* calcd for $C_{10}H_{17}O$ [$(M+H)^+$] 153.1274, found 153.1273.

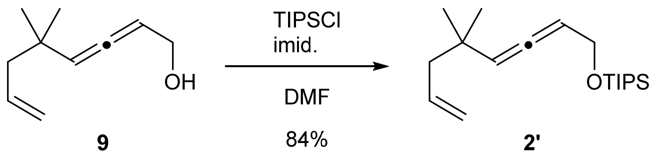

Compound 2': To a solution of alcohol **9** (1.01 g, 6.64 mmol) in DMF (66 mL) were added imidazole (1.00 g, 14.7 mmol) and TIPSCl (1.6 mL, 7.55 mmol) at 0 °C. After the solution was stirred at room temperature for 1 h, the reaction was quenched with satd. aq. $NaHCO_3$. The mixture was extracted with ether three times, and the combined organic layers were washed with water, and brine. The organic layer was dried over Na_2SO_4, filtered and concentrated under reduced pressure. The residue was purified by silica gel column chromatography (hexane) to give **2'** (1.71 g, 84%) as a colorless oil: ^{1}H NMR (500 MHz, $CDCl_3$) δ 5.81 (m, 1H), 5.32 (dd, 2H, J = 6.6, 12.5 Hz), 5.17 (m, 1H). 5.02 (m, 2H), 4.25 (m, 2H), 2.07 (d, 2H, J = 7.4 Hz), 1.14-1.05 (m, 21H), 1.01 (s, 6H); 13C NMR (125 MHz, $CDCl_3$) δ201.4, 135.7, 117.1, 102.9, 93.9, 62.4, 47.7, 34.9, 27.8, 27.8, 18.2, 12.2; IR (ATR) ν = 2959, 2952, 2892, 2865, 1463, 1091 cm^{-1}; HRMS (ESI) *m/z* calcd for $C_{19}H_{37}O$ Si[$(M+H)^+$] 309.2608, found 309.2600.

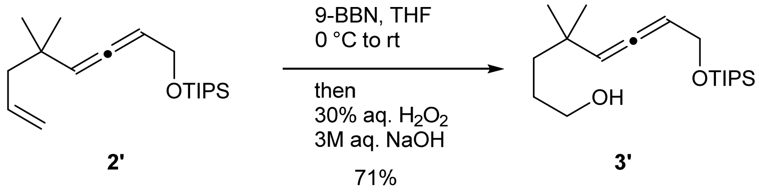

Compound 3': To a solution of silyl ether **2'** (28.7 g, 93.1 mmol) in THF (465 mL) was added 9-BBN (0.5 M in THF, 186 mL, 93.1 mmol) at 0 °C dropwise. The reaction mixture was stirred at room temperature for 25 min. Additional 9-BBN (93 mL, 46.6 mmol) was added at 0 °C three times every 25 min. After confirming the starting material disappeared by TLC, 3 M aq. NaOH (186 mL) and 30% H_2O_2 (63 mL) were added at 0 °C, and the resultant solution was stirred at room temperature for 25 min. After addition of satd. aq. NH_4Cl, the reaction mixture was extracted with EtOAc three times. The combined organic layers were washed with brine, dried over Na_2SO_4, filtered and concentrated under reduced pressure. The residue was purified by silica gel column chromatography (10-25% EtOAc/hexane) to afford **3'** as a colorless oil (21.5 g, 71%): ^{1}H NMR (500

MHz, $CDCl_3$) δ 5.31 (dd, 1H, *J* = 5.7, 10.7 Hz), 5.11 (m,1H), 4.17 (m,2H), 3.55 (m, 2H), 1.53-1.44 (m, 1H), 1.45-1.31 (m, 3H), 1.16-1.04 (m, 21H), 0.94 (s, 6H); ^{13}C NMR (125 MHz, $CDCl_3$) δ 201.6, 102.8, 93.6, 63.7, 62.4, 39.0, 34.4, 28.4, 28.3, 18.2, 12.2; IR (ATR) ν = 3345, 2956, 2941, 2892, 2865, 1463 cm^{-1}; HRMS (ESI) *m/z* calcd for $C_{19}H_{38}O_2SiNa$ [$(M+Na)^+$] 349.2533, found 349.2524.

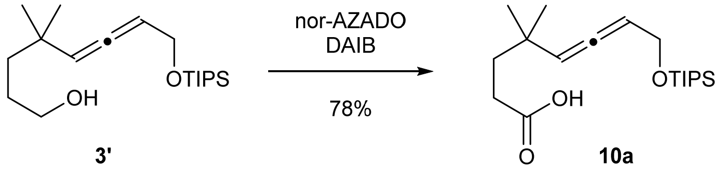

Compound 10a: To a solution of alcohol **3’** (99.3 mg, 0.304 mmol) in CH_2Cl_2-pH 7 phosphate buffer (1:1, 3.0 mL) were added nor-AZADO (10.0 mg, 0.0724 mmol) and $PhI(OAc)_2$ (211 mg, 0.655 mmol) at 0 °C. After the reaction mixture was stirred for 7 h at room temperature, additional $PhI(OAc)_2$ (40.6 mg, 0.126 mmol) was added. The solution was stirred for 2 h. The reaction was then quenched with 10% aq. $Na_2S_2O_3$. The mixture was extracted with $CHCl_3$ three times and dried over Na_2SO_4. The filtrate was concentrated under reduced pressure and the residue was purified by silica gel column chromatography (10-35% EtOAc/hexane) to afford **10a** as a colorless oil (80.2 mg, 78%): ^{1}H NMR (500 MHz, $CDCl_3$) δ 5.34 (dd, 1H, *J* = 6.0, 12.2 Hz), 5.09 (m, 1H), 4.25 (m, 2H), 2.36 (t, 2H, *J* = 8.6 Hz), 1.68 (t, 2H, *J* = 8.3 Hz), 1.14-1.04 (m, 21H), 1.03 (s, 6H); ^{13}C NMR (125 MHz, $CDCl_3$) δ 201.7, 180.8, 102.0, 94.2, 62,2, 37.4, 34.6, 30.2, 27.9, 27.7, 18.2, 12.2; IR (ATR) ν = 2959, 2942, 2892, 2865, 1711 cm^{-1}; HRMS (ESI) *m/z* calcd for $C_{19}H_{36}O_3SiNa$ [$(M+Na)^+$] 363.2326, found 363.2325.

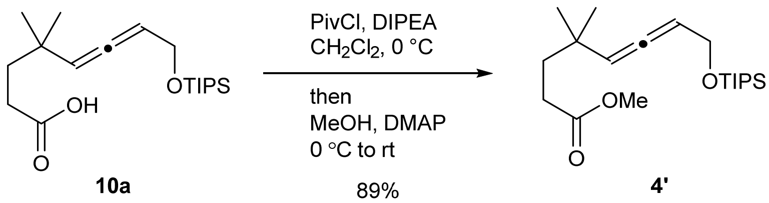

Compound 4’: To a mixed solution of carboxylic acid **10a** (80.2 mg, 0.236 mmol) and DIPEA (0.098 mL, 0.565 mmol) in CH_2Cl_2 (2.4 mL) was added PivCl (0.035 mL, 0.283 mmol) dropwise 0 °C for 5 min. After 5 min, MeOH (0.029 mL, 0.707 mmol) and DMAP (5.6 mg, 0.0458 mmol) were added. The solution was stirred at room temperature overnight. The reaction was then quenched with satd. aq. $NaHCO_3$ at 0 °C and extracted with $CHCl_3$ three times. The combined organic layers were dried over Na_2SO_4, filtered, and concentrated. The residue was purified by silica gel column chromatography (3-7% EtOAc/hexane) to afford **4’** (74.0 mg, 89%) as a colorless oil: ^{1}H NMR (500 MHz, $CDCl_3$) δ 5.25 (1H, dd, *J* = 6.6, 12.6 Hz), 5.01 (m, 1H), 4.16 (m, 2H),

3.59 (s, 3H), 2.24 (t, 2H, J = 8.3 Hz), 1.59 (t, 2H, J = 8.3 Hz), 1.06-0.96 (m, 21H), 0.94 (s, 6H); ^{13}C NMR (125 MHz, $CDCl_3$) δ 201.4, 174.6, 101.8. 93.8, 61.9, 51.5, 37.5, 34.3, 29.9, 27.6, 27.4, 26.4, 17.9, 11.9; IR (ATR) ν = 3000, 1770, 1757, 1749, 1245 cm^{-1}; HRMS (ESI) *m/z* calcd for $C_{20}H_{38}O_3SiNa$ [$(M+Na)^+$] 377.2482, found 377.2500.

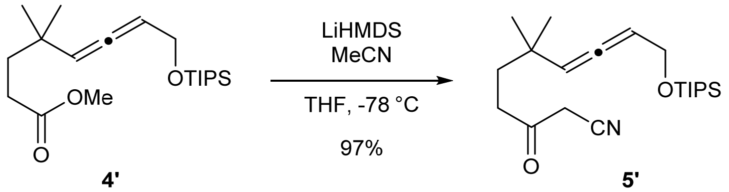

4' 5'

Compound 5': To a solution of LiHMDS (1.3 M in THF, 0.288 mL, 0.374 mmol) in THF (1.9 mL) was added MeCN (0.020 mL, 0.374 mmol) dropwise at −78 °C. After 30 min, methyl ester **4'** in THF (25 mL) was added dropwise via dropping funnel at −78 °C. The resultant solution was stirred for at −78 °C for 5 min. After the solution was diluted with Et_2O, 1 M aq. HCl and satd. aq. NH_4Cl were added. The mixture was extracted with Et_2O three times. The combined organic layers were washed with brine, dried over Na_2SO_4, filtered and concentrated under reduced pressure. The residue was purified by silica gel column chromatography (7%-20% EtOAc/hexane) to afford **5'** as a colorless oil (65.9 mg, 97%): ^{1}H NMR (500 MHz, $CDCl_3$) δ 5.33 (dd, 1H J = 6.3, 11.9 Hz), 5.05 (m, 1H), 4.23 (m, 2H), 3.47 (s, 2H), 2.59 (m, 2H), 1.64 (m, 2H), 1.15-1.03 (m, 21H), 1.02 (s, 3H), 1.00 (s, 3H); ^{13}C NMR (125 MHz, $CDCl_3$) δ 201.4, 197.7. 113.7, 101.6, 94.1, 61.8, 38.1, 35.5, 34.2, 31.9, 28.1, 27.3, 17.9, 11.9; IR (ATR) ν = 3734, 2989, 1770, 1758, 1733, 1245, 1053 cm^{-1}; HRMS (ESI) *m/z* calcd for $C_{21}H_{37}NO_2SiNa$ [$(M+Na)^+$] 386.2486, found 386.2486.

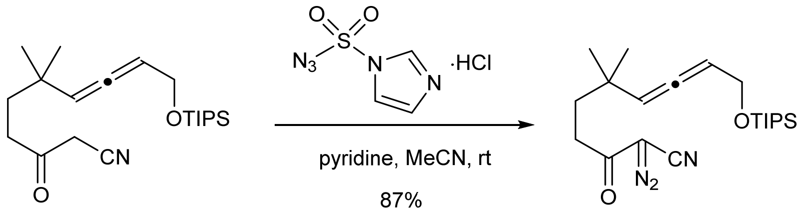

5' 6a

Compound 6a: To a solution of **5'** (58.7 mg, 0.161 mmol) in MeCN (1.6 mL) were added pyridine (47 mL, 0.581 mmol) and imidazole-1-sulfonylazide (33.5 mg, 0.193 mmol) at room temperature. After stirring overnight, an additional pyridine (13 μL, 0.161 mmol) and imidazole-1-sulfonylazide (28.6 mg, 0.164 mmol) were added to the reaction mixture. After stirring for 6 h, the reaction was quenched with satd. aq. NH_4Cl, and the resultant mixture was extracted with EtOAc three times. The combined organic layers were washed with brine, dried over Na_2SO_4, and filtered. The filtrate was concentrated and the residue was purified by silica gel column chromatography (3%-10%

EtOAc/hexane) to afford **6a** as a yellowish oil (54.5 mg, 87%): ^{1}H NMR (500 MHz, $CDCl_3$) δ 5.35 (dd, 1H, *J* = 6.6, 12.5 Hz), 5.07 (m, 1H), 4.29-4.19 (m, 2H), 2.60 (t, 2H, *J* = 7.7 Hz), 1.69 (t, 2H, *J* = 7.7 Hz), 1.19-1.04 (m, 21H), 1.03 (s, 3H), 1.02 (s, 3H); ^{13}C NMR (125 MHz, $CDCl_3$) δ 201.4, 190.5, 108.4, 101.5, 94.2, 61.9, 36.6, 35.6, 34.5, 27.9, 27.3, 17.9, 11.9; IR (ATR) ν = 3734, 3000, 1758, 1244, 1055 cm^{-1}; HRMS (ESI) *m/z* calcd for $C_{21}H_{35}N_3O_2SiNa$ [(M+Na)$^+$] 412.2391, found 412.2403.

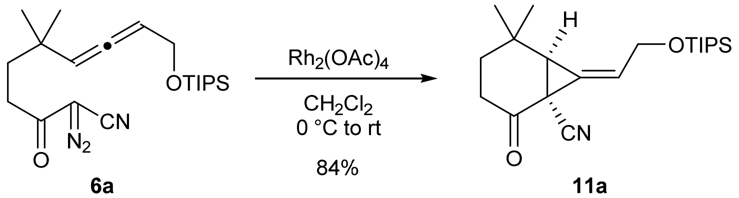

6a **11a**

Compound 11a: To a solution of **6a** (51.3 mg, 0.132 mmol) in CH_2Cl_2 (2.6 mL) was added $Rh_2(OAc)_4$ (3.3 mg, 7.47 μmol) at 0 °C. The solution was stirred at room temperature for 30 min. The resulting solution was concentrated, and purified by silica gel column chromatography (7-15% EtOAc/hexane) to afford **11a** as a colorless oil (40.1 mg, 84%): ^{1}H NMR (500 MHz, $CDCl_3$) δ 6.42 (dt, 1H, *J* = 2.6, 6.0 Hz), 4.44 (m, 2H), 2.76 (s, 1H), 2.32 (dd, 2H *J* = 4.3, 9.2 Hz), 1.76-1.57 (m, 1H), 1.40-1.34 (m, 1H), 1.23 (s, 3H), 1.20 (s, 3H), 1.14-0.98 (m, 21H); ^{13}C NMR (125 MHz, $CDCl_3$) δ 197.5, 126.0, 122.0, 116.7, 62.3, 42.8, 32.7, 30.8, 30.6, 29.1, 27.6, 25.1, 17.9, 11.8; IR (ATR) ν = 3734, 2992, 1770, 1758, 1702, 1246 cm^{-1}; HRMS (ESI) *m/z* calcd for $C_{21}H_{36}NO_2Si$ [(M+H)$^+$] 362.2510, found 362.2520.

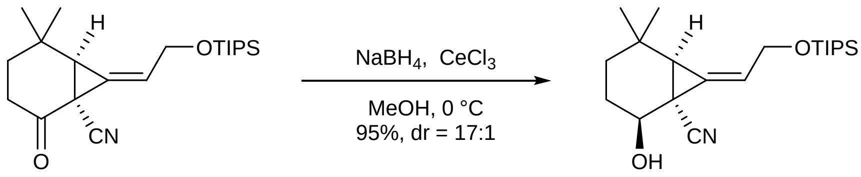

11a **6'**

Compound 6': To a solution of **11a** (33.7 mg, 0.0932 mmol) in MeOH (1.9 mL) were added $CeCl_3$·$7H_2O$ (41.1 mg, 0.110 mmol) and $NaBH_4$ (5.4 mg, 0.143 mmol) at 0 °C. After stirring for 5 min, the reaction was quenched with water, and the resultant solution was extracted with ether three times. The combined organic layers were washed with brine, dried over Na_2SO_4, filtered and concentrated under reduced pressure. The residue was purified by silica gel column chromatography (10%-15% EtOAc/hexane) to afford **6'** as a white solid (32.1 mg, 95%, dr =17:1): ^{1}H NMR (500 MHz, $CDCl_3$) δ 6.23 (s, 1H), 4.37 (d, 2H, *J* = 6.0 Hz), 4.11 (s, 1H), 2.26 (br, OH), 2.18 (s, 1H), 1.56-1.48 (m, 1H), 1.44 (t, 1H, *J* = 13.5 Hz), 1.24 (s, 3H), 1.06 (s, 18H), 1.16-1.03 (m, 5H), 1.01 (s, 3H); ^{13}C NMR (125 MHz, $CDCl_3$) δ 124.3, 123.1, 64.3, 62.7, 35.1, 30.7, 30.0, 29.5,

29.4, 25.9, 18.3, 17.9, 17.9, 11.9; IR (ATR) ν = 2994, 2950, 1770, 1758, 1375, 1245, 1057 cm^{-1}; HRMS (ESI) *m/z* calcd for $C_{21}H_{37}NO_2SiNa$ $[(M+Na)^+]$ 386.2486, found 386.2469.

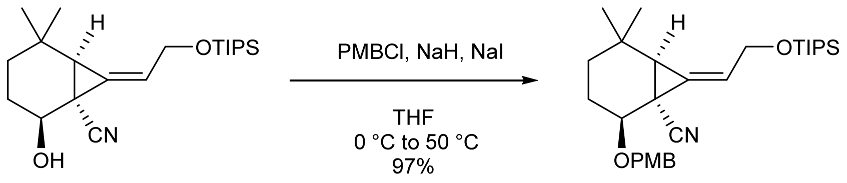

6' **12**

Compound 12: To a solution of **S6** (6.19 g, , 17.0 mmol) in THF (170 mL) was added NaH (60% in mineral oil, 921 mg, 23.0 mmol) slowly at 0 °C. After the reaction mixture was stirred at 0 °C for 30 min, PMBCl (3.5 mL, 25.5 mmol) and NaI (777 mg, 5.18 mmol) were added. The mixture was then stirred at 50 °C for 12 h. The reaction was quenched with satd. aq. NH_4Cl at 0 °C, and the resultant mixture was extracted with EtOAc three times. The combined organic layers were washed with brine, dried over Na_2SO_4, filtered and concentrated. The residue was purified by silica gel column chromatography 3-15 % EtOAc/hexane) to afford **12** as a colorless oil (8.09 g, 97%): ^1H NMR (500 MHz, $CDCl_3$) δ 7.37 (d, 2H, *J* = 8.3Hz), 6.90 (d, 2H, *J* = 8.3 Hz), 6.21 (s, 1H), 4.67 (d, 1H, *J* = 11.5 Hz), 4.60 (d, 1H, *J* = 11.5 Hz), 4.36 (s, 2H), 3.81(s, 3H), 3.74 (s, 1H), 2.15 (s, 1H), 1.47-1.36 (m, 2H), 1.22 (s, 3H), 1.15-0.91 (m, 23H), 1.00 (s, 3H); ^{13}C NMR (125 MHz, $CDCl_3$) δ 159.2, 129.9, 129.4, 124.7, 123.1, 120.7, 113.8, 71.4, 71.2, 62.7, 55.2, 35.2, 30.6, 30.4, 29.6, 29.5, 22.8, 17.9, 17.0, 11.9; IR (ATR) ν = 2994, 2866, 1770, 1758, 1513, 1246 cm^{-1}; HRMS (ESI) *m/z* calcd for $C_{29}H_{45}NO_3Si$ $[(M+Na)^+]$ 506.3061, found 506.3051.

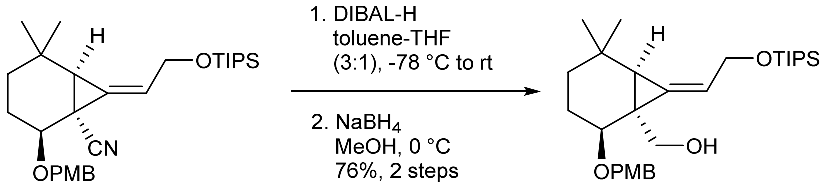

12 **13**

Compound 13: To a solution of **12** (27 mg, 56.2 μmol) in toluene-THF (3:1, 1 mL) was added slowly DIBAL-H (1.0 M solution in toluene, 112 μL, 112 μmol) at −78 °C. The solution was warmed up to room temperature gradually. After the starting material completely consumed, acetone (100 μL), EtOAc (100 μL), and pH 7 phosphate buffer (100 μL) were added to the solution. The resulting mixture was stirred for 20 min vigorously. Na_2SO_4 was then added and the mixture was stirred for 30 min. After filtration through Na_2SO_4 and silica gel, the filtrate was concentrated to afford aldehyde as a colorless oil (26 mg, crude), which was used for the next reaction without further purification: ^1H NMR (500 MHz, $CDCl_3$) δ 9.23 (s, 1H), 7.31 (d, 2H, *J* = 8.3 Hz), 6.88 (d, 2H, *J* = 8.0 Hz), 5.90 (s, 1H), 4.60 (d, 1H, *J* = 11.5 Hz), 4.53 (d, 1H, *J* = 11.2 Hz), 4.36 (d, 2H, *J*

=6.0 Hz), 4.22 (s, 1H), 3.81 (s, 3H), 2.22 (s, 1H), 1.69-1.60 (m, 1H), 1.55 (t, 1H, *J* = 14.3 Hz), 1.19 (s, 3H), 1.14-1.00 (m, 23H), 1.05 (s, 3H); IR (ATR) ν = 2942, 2865, 2725, 1769, 1758, 1710, 1613, 1513 cm^{-1}; HRMS (ESI) *m/z* calcd for $C_{29}H_{46}O_4SiNa$ $[(M+Na)^+]$ 509.3058, found 509.3040. To a solution of the above aldehyde (26 mg, crude) in MeOH (1 mL) was added $NaBH_4$ (2.6 mg, 68.7 μmol) at 0 °C. The reaction mixture was stirred at 0 °C for 10 min. After addition of water, the mixture was extracted with Et_2O three times and washed with brine. The combined organic layers were dried over Na_2SO_4, filtered, concentrated, and purified by silica gel column chromatography (3-15 % EtOAc/hexane) to afford **13** as a colorless oil (21 mg, 76%, 2 steps): ^1H NMR (500 MHz, $CDCl_3$) δ 7.29 (d, 2H, *J* = 8.3 Hz), 6.90 (d, 2H, *J* = 8.3 Hz), 5.83 (s, 1H), 4.60 (d, 1H, *J* = 11.2 Hz), 4.38 (d, 1H, *J* = 11.2 Hz), 4.34-4.26 (m, 2H), 3.97 (d, 1H, *J* = 11.5 Hz), 3.92 (br, OH), 3.81 (s, 3H), 3.21 (d, 1H, *J* = 10.0 Hz), 3.00 (t, 1H, *J* = 10.9 Hz), 1.72-1.56 (m, 2H), 1.47-1.38 (m, 2H), 1.26 (s, 1H), 1.19 (s, 3H), 1.15-0.77 (m, 21H), 0.98 (s, 3H); ^{13}C NMR (125 MHz, $CDCl_3$) δ 159.2, 131.3, 130.2, 129.3, 118.7, 113.9, 75.9, 75.8, 70.5, 68.3, 63.6, 55.2, 31.8, 31.1, 29.9, 29.2, 29.2, 22.6, 18.0, 12.0; IR (ATR) ν = 3537, 2941, 2864, 1613, 1513, 1462, 1248 cm^{-1}; HRMS (ESI) *m/z* calcd for $C_{29}H_{49}O_4Si$ $[(M+H)^+]$ 511.3214, found 511.3196.

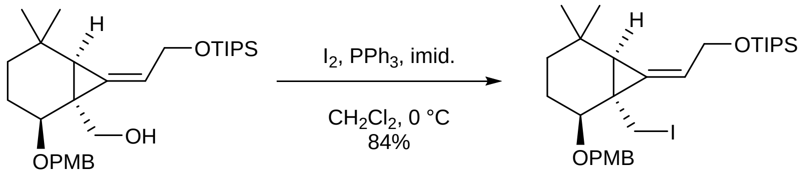

13 **7'**

Compound 7': To a solution of imidazole (642 mg, 9.43 mmol) and PPh_3 (834 mg, 3.18 mmol) in CH_2Cl_2 (7.8 mL) was added I_2 (854 mg, 3.36 mmol) at 0 °C in dark. An alcohol **13** in CH_2Cl_2 (7.8 mL) was then added to the mixture through cannula after stirred for 5 min. The reaction mixture was stirred at 0 °C in dark for 20 min. the resulting mixture was quenched with satd. aq. $Na_2S_2O_3$ and extracted with $CHCl_3$ three times. The combined organic layers were dried over Na_2SO_4, filtered and concentrated. The residue was purified by silica gel column chromatography (3-10% EtOAc/hexane) to afford alkyl iodide **7'** as a yellowish oil (785 mg, 84%): ^1H NMR (500 MHz $CDCl_3$) δ 7.36 (d, 2H, *J* = 8.3 Hz), 6.90 (d, 2H, *J* = 8.3 Hz), 6.14 (s, 1H), 4.56 (d, 1H, *J* = 10.9 Hz), 4.46 (d, 2H, *J* = 10.6 Hz), 4.34 (d, 1H, *J* = 8.6 Hz), 3.91 (s, 1H), 3.81 (s, 3H), 2.82 (d, *J* = 8.9 Hz), 1.69-1.41 (m, 2H), 1.43 (s, 1H), 1.16-0.97 (m, 1H), 0.91-0.75 (m, 1H) 1.13 (s, 3H), 1.12-0.98 (m, 21H), 0.93 (s, 3H); 13C NMR (125 MHz, $CDCl_3$) δ 159.0, 133.0, 131.0, 129.5, 117.8, 113.7, 72.3, 71.3, 63.3, 55.2, 36.5, 32.7, 31.3, 30.5, 29.4, 28.2, 21.7, 18.0, 17.7, 12.0; IR (ATR) ν = 2942, 2864, 1770, 1614, 1514, 1464, 1247, 1219 cm^{-1}; HRMS (ESI) *m/z* calcd for $C_{29}H_{47}O_3SiINa$ $[(M+Na)^+]$ 621.2231, found 621.2220.

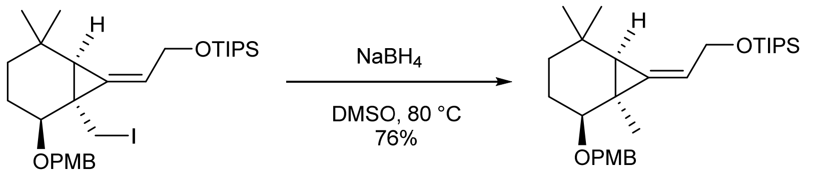

7' 14

Compound 14: To a solution of alkyl iodide **7’** (17.7 mg, 29.6 μmol) in DMSO (100 μL) was added $NaBH_4$ (2.2 mg, 58.2 μmol). The solution was stirred at 80 °C for 1 h. The reaction was quenched with satd. aq. NH_4Cl at room temperature. After 30 min, the resultant mixture was extracted with Et_2O three times. The combined organic layers were washed with brine, dried over Na_2SO_4, filtered and concentrated. The residue was purified by silica gel column chromatography (1-7% EtOAc/hexane) to afford **14** (11.5 mg, 76%) as a colorless oil: ^{1}H NMR (500 MHz, $CDCl_3$) δ 7.30 (d, 2H, *J* = 8.3 Hz), 6.88 (d, 2H, *J* = 8.3 Hz), 5.82 (s, 1H), 4.55 (d, 1H, *J* = 11.5 Hz), 4.37 (d, 1H, *J* = 11.7 Hz), 4.36-4.26 (m, 2H), 3.81 (s, 3H), 3.57 (s, 1H), 1.52-1.40 (m, 2H), 1.37-1.28 (m, 1H), 1.26 (s, 1H), 1.19 (s, 3H), 1.14 (s, 3H), 1.13-1.00 (m, 21H), 0.99-0.79 (m, 1H), 0.92 (s, 3H); ^{13}C NMR (125 MHz, $CDCl_3$) δ 158.9, 136.1, 131.4, 129.0, 117.2, 113.6, 74.7, 70.6, 63.9, 55.2, 33.6, 32.7, 31.3, 30.2, 29.7, 23.3, 22.5, 21.4, 18.0, 12.0; IR (ATR) ν = 2993, 1770, 1373, 1245 cm^{-1}; HRMS (ESI) *m/z* calcd for $C_{29}H_{48}O_3SiNa$ [$(M+Na)^+$] 495.3265, found 495.3249.

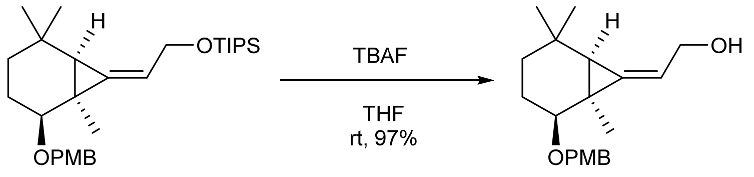

14 5

Compound 5: To a solution of **14** (486 mg, 1.03 mmol) in THF (10 mL) was added TBAF (1.0 M in THF) at room temperature. The solution was stirred for 1 h, and then diluted with EtOAc and satd. aq. NH_4Cl, extracted with EtOAc three times. The combined organic layers were washed with brine, dried over Na_2SO_4, filtered and concentrated. The residue was purified by silica gel column chromatography to afford **5** (313 mg, 97%) as a colorless oil; ^{1}H NMR (500 MHz, $CDCl_3$) δ 7.30 (d, 2H, *J* = 8.3 Hz), 6.89 (d, 2H, *J* = 8.3 Hz), 5.88 (t, 1H, *J* = 6.0 Hz), 4.56 (d, *J* = 11.5 Hz), 4.37 (d, *J* = 11.5 Hz), 4.24 (m, 2H), 3.58 (t, 1H, *J* = 3.3 Hz), 1.53-1.45 (m, 1H), 1.35-1.24 (m, 2H), 1.22 (s, 1H), 1.20 (s, 3H), 1.16 (s, 3H), 0.98 (m, 1H), 0.96 (s, 3H); ^{13}C NMR (125 MHz, $CDCl_3$) δ 158.9, 131.1, 128.9, 113.7, 74.9, 71.7, 63.3, 55.2, 36.5, 33.4, 33.0, 29.6, 29.0, 28.4, 26.7, 24.4, 23.8, 20.4; IR (ATR) ν = 3407, 2992, 2953, 2861, 1768, 1756 cm^{-1}; HRMS (ESI) *m/z* calcd for $C_{20}H_{28}O_3Na$ [$(M+Na)^+$] 339.1931, found 339.1927.

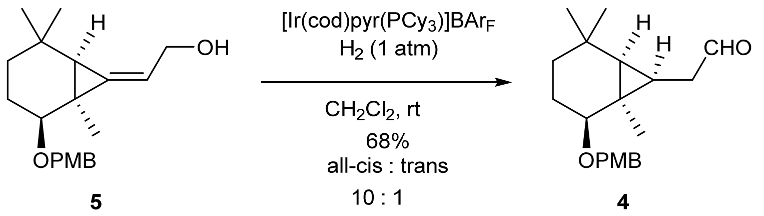

Compound 4: To a solution of **5** (41 mg, 0.132 mmol) in CH_2Cl_2 (2.6 mL) was added [Ir(cod)(pyr)(PCy_3)]BAr_F (2.4 mg, 1.58 μmol) at room temperature. After the reaction mixture was babbled with H_2 gas via needle for 3 min, the orange solution turned yellow. After 5 min, additional [Ir(cod)(pyr)(PCy_3)]BAr_F (4.7 mg, 3.08 μmol) was added and H_2 gas was babbled for 1 min. After the starting material was completely consumed, Et_3N (two drops) was added, and the solution was concentrated. The residue was purified by silica gel column chromatography (7-20% EtOAc/hexane) to afford **4** (28 mg, 68%, dr = 10:1) as a colorless oil: ^{1}H NMR (500 MHz, $CDCl_3$) δ 9.77 (s, 1H), 7.28 (d, 2H, J = 8.3 Hz), 6.87 (d, 2H, J = 8.3 Hz), 4.54 (d, 1H, J = 11.7 Hz), 4.40 (d, 1H, J = 11.7 Hz), 3.79 (3H, s), 3.05 (dd, 1H, J = 11.7, 4.3 Hz), 2.65 (dd, 1H, J = 17.9, 6.2 Hz), 2.43 (dd, 1H, J = 17.9, 8.9 Hz), 1.66-1.59 (m, 1H), 1.48 (dd, 1H, J = 25.6, 13.6 Hz), 1.35 (d, 1H, J = 14.0 Hz), 1.24 (s, 3H), 1.11 (d, 1H, J = 7.7 Hz), 1.10 (s, 3H), 0.94-0.84 (m, 1H), 0.92 (s, 3H), 0.50 (d, 1H, J = 9.5 Hz); ^{13}C NMR (125 MHz, $CDCl_3$) δ 201.7, 128.8, 128.7, 114.0, 113.4, 73.9, 71.6, 55.3, 40.0, 36.4, 33.1, 32.6, 29.2, 28.6, 26.6, 23.3, 21.0, 20.5; IR (ATR) ν = 3369, 2957, 2864, 2718, 1725, 1613, 1512 cm^{-1}; HRMS (ESI) *m/z* calcd for $C_{20}H_{28}O_3Na$ [$(M+Na)^+$] 339.1931, found 339.1937. The same procedure was applied to a gram-scale synthesis of compound **4** (1.07 g, 78%, dr 9.5:1).

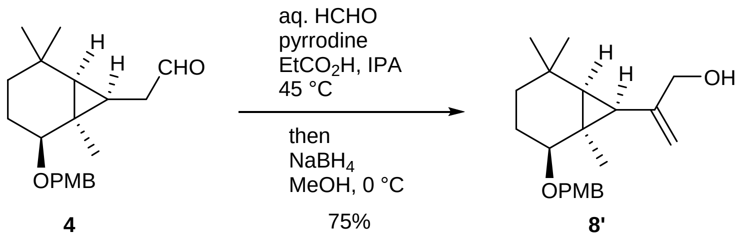

Compound 8': To a solution of aldehyde **4** (57.9 mg, 0.183 mmol) in 2-propanol (1.8 mL) were added 37% aq. HCHO (16.3μL, 0.201 mmol), pyrrolidine (7.5 μL, 0.0913 mmol) and propionic acid (7.8 μL, 0.0913 mmol) at room temperature. The mixture was stirred at 45 °C for 24 h. MeOH (1.8 mL) and $NaBH_4$ (10.4 mg, 0.275 mmol) were added to the resulting solution at 0 °C, and the mixture was stirred at 0 °C for 20 min. The reaction was then quenched with satd. aq. NH_4Cl and extracted with EtOAc three times. The combined organic layers were washed with brine, dried over Na_2SO_4 and filtered. The filtrate was concentrated and the residue was purified by silica gel column chromatography (7-20% EtOAc/hexane) to afford **8'** (45.2 mg, 75%) as a colorless oil: ^{1}H NMR

(500 MHz, $CDCl_3$) δ 7.27 (d, 2H, J = 8.3 Hz), 6.87 (d, 2H, J = 8.3 Hz), 5.25 (s, 1H), 4.99 (s, 1H), 4.49 (d, 1H, J = 11.5 Hz), 4.44 (d, 1H, J = 11.5 Hz), 4.03 (d, 1H, J = 14.3 Hz), 3.92 (d, 1H, J = 14.3 Hz), 3.80 (s, 3H), 3.31 (dd, 1H, J = 9.7, 4.9 Hz), 1.65-1.62 (m, 2H), 1.58-1.50 (m, 1H), 1.29-1.24 (m, 1H), 1.18-1.13 (m, 1H), 1.26 (s, 3H), 1.14 (s, 3H), 1.11 (s, 3H), 0.71 (d, 1H, J = 9.5 Hz); ^{13}C NMR (125 MHz, $CDCl_3$) δ 158.9, 145.6, 131.1, 129.0, 113.6, 111.8, 74.2, 71.2, 67.3, 60.3, 55.2, 37.5, 34.0, 33.0, 32.1, 30.2, 29.8, 25.3, 24.4, 22.8, 21.0, 14.1; IR (ATR) ν = 3421, 2950, 2864, 1738, 1613, 1512, 1244 cm^{-1}; HRMS (ESI) *m/z* calcd for $C_{21}H_{30}O_3Na$ [$(M+Na)^+$] 353.2087, found 353.2072.

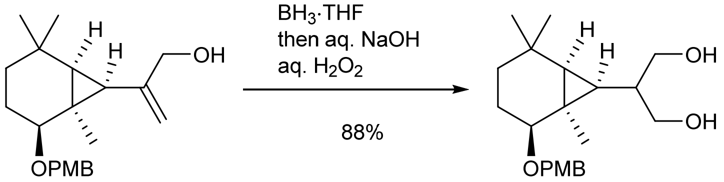

8' 3

Compound 3: To a solution of alkene **8'** (273 mg, 0.827 mmol) in THF (8.3 mL) was added BH_3·THF (1.0M in THF, 910 µL, 0.910 mmol) at 0 °C dropwise. The solution was stirred at room temperature for 20 min. Additional BH_3·THF (750 µL, 0.750 mmol) was added to 0 °C, and the resultant solution was stirred for 50 min at room temperature. 3M aq. NaOH (1.7 mL) and 30% H_2O_2 (563 µL) were then added at 0 °C. The resultant mixture was stirred at room temperature for 15 min. The reaction was quenched with satd. aq. NH_4Cl at 0 °C, and the mixture was extracted with EtOAc three times. The combined organic layers were washed with brine, dried over Na_2SO_4, filtered and concentrated under reduced pressure. The residue was purified by silica gel column chromatography (35-55% EtOAc/hexane) to afford **3** as a colorless oil (255 mg, 88%): ^{1}H NMR (500 MHz, $CDCl_3$) δ 7.31 (d, 2H, J = 8.6 Hz), 6.90 (d, 2H, J = 8.6 Hz), 4.55 (dd, 2H, J = 25.5, 11.5 Hz), 4.06 (br, 1H), 3.87-3.73 (m, 4H), 3.72-3.64 (m, 1H), 3.60 (t, 1H, J = 8.9 Hz), 3.20 (dd, 1H, J = 11.5, 4.0 Hz), 2.43 (br, OH, 2.09 (br, OH), 1.77-1.64 (m, 2H), 1.53-1.35 (m, 2H), 1.15 (s, 3H), 1.11 (s, 3H), 1.05 (s, 3H), 1.02-0.82 (m, 1H), 0.40 (d, 1H, J = 9.2 Hz), 0.18 (t, 1H, 11.2 Hz); ^{13}C NMR (125 MHz, $CDCl_3$) δ 159.0, 130.9, 129.0, 128.8, 113.7, 75.1, 71.2, 68.7, 67.3, 55.2, 38.5, 36.7, 33.7, 33.3, 30.1, 29.9, 26.5, 25.8, 24.1, 20.6; IR (ATR) ν = 3369, 2953, 2864, 1737, 1613, 1512, 1244 cm^{-1}; HRMS (ESI) *m/z* calcd for $C_{21}H_{32}O_4Na$ [$(M+Na)^+$] 371.2193, found 371.2192.

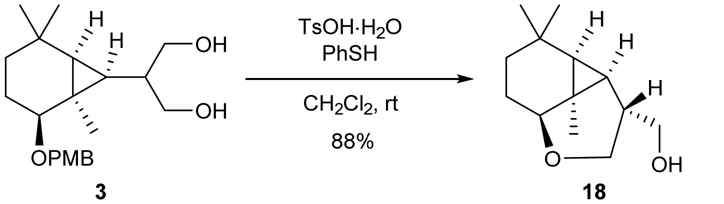

3 18

Compound 18: To a solution of diol **3** (21 mg, 0.0606 mmol) in CH_2Cl_2 (1.2 mL) were added PhSH (62 μL, 0.606 mmol) and TsOH·H_2O (0.0195 mmol) at room temperature. The solution was stirred for 6 h. The reaction was quenched with satd. aq. $NaHCO_3$, and the resultant mixture was extracted with EtOAc three times. The combined organic layers were washed with brine, dried over Na_2SO_4, filtered and concentrated under reduced pressure. The residue was purified by silica gel column chromatography (15-60% EtOAc/hexane) to afford **18** as a colorless oil (11 mg, 88%): ^{1}H NMR (500 MHz, $CDCl_3$) δ 4.11 (s, 1H), 3.73 (dd, 1H, *J* = 3.7, 10.0 Hz), 3.67 (dd, 1H, *J* = 6.0, 10.9 Hz), 3.56 (t, 1H, *J* = 8.3 Hz), 2.99 (t, 1H, *J* = 10.6 Hz), 1.88-1.79 (m, 1H), 1.55-1.42 (m, 3H), 1.39-1.31 (m, 1H), 1.17 (s, 3H), 1.05 (s, 6H), 1.02-0.98 (m, 1H), 0.49 (dd, 1H, *J* = 6.3, 8.9 Hz), 0.36 (d, 1H, *J* = 9.2 Hz); ^{13}C NMR (125 MHz, $CDCl_3$) δ 71.8, 65.3, 64.0, 34.6, 32.5, 31.0, 29.4, 29.0, 28.5, 28.0, 27.0, 25.3; IR (ATR) ν = 3423, 2994, 1770, 1463, 1381, 1245 cm^{-1}; HRMS (ESI) *m/z* calcd for $C_{13}H_{22}O_2Na$ [$(M+Na)^+$] 211.1693, found 211.1688.

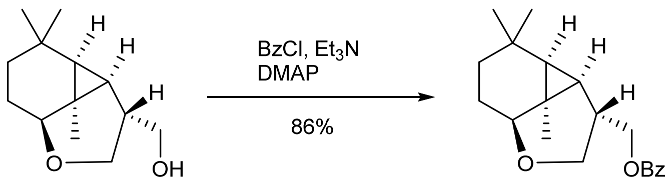

18 **20**

Compound 20: To a solution of alcohol **18** (253 mg, 1.21 mmol) in CH_2Cl_2 (24 mL) were added Et_3N (676 μL, 4.86 mmol), DMAP (78.0 mg, 0.638 mmol) and BzCl (282 μL, 2.43 mmol) at room temperature. The solution was stirred for 1 h. The reaction was quenched with satd. aq. $NaHCO_3$, and the resultant mixture was extracted with $CHCl_3$ three times. The combined organic layers were dried over Na_2SO_4, filtered and concentrated. The residue was purified by silica gel column chromatography (3-5% EtOAc/hexane) to afford **20** as a colorless oil (327 mg, 86%): ^{1}H NMR (500 MHz, $CDCl_3$) δ 8.04 (d, 2H, *J* = 8.3 Hz), 7.55 (t, 1H, *J* = 7.2 Hz), 7.43 (t, 2H, *J* = 7.6 Hz), 4.34 (dd, 1H, *J* = 6.3, 10.9 Hz), 4.18 (dd, 1H, *J* = 7.7, 10.7 Hz), 4.13 (s, 1H), 3.76 (dd, 1H, *J* = 3.4, 10.0 Hz), 3.07 (t, 1H, *J* = 10.5 Hz), 2.18-2.06 (m, 1H), 1.57-1.47 (m, 1H), 1.43-1.31 (m, 1H), 1.18 (s, 3H), 1.07 (s, 3H), 1.05 (s, 3H), 1.06-0.99 (m, 1H), 0.61 (dd, 1H, *J* = 6.4, 9.0 Hz), 0.39 (d, 1H, *J* = 9.2 Hz); ^{13}C NMR (125 MHz, $CDCl_3$) δ 166.5, 133.0, 130.0, 129.5, 128.3, 71.8, 66.5, 63.8, 32.5, 31.8, 30.9, 29.3, 28.9, 28.4, 27.0, 25.5, 12.4; IR (ATR) ν = 2953, 2864, 1719, 1602, 1451, 1267, 1096 cm^{-1}; HRMS (ESI) *m/z* calcd for $C_{20}H_{26}O_3Na$ [$(M+Na)^+$] 337.1774, found 337.1761.

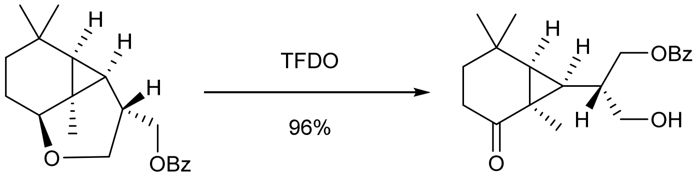

20 **22**

Compound 22: To a solution of ether **20** (17.0 mg, 0.0547 mmol) in CH_2Cl_2 (1 mL) was added TFDO (in trifluoroacetone, *ca.* 0.5 M, 20 drops) was added at −78 °C. After the solution was stirred for 5 min at −78 °C, the TFDO solution was added until ether **17** was completely completed. The reaction solution was concentrated, and the residue was purified by silica gel column chromatography (25-50% EtOAc/hexane) to afford **22** as a colorless oil (17.0 mg, 96%): ^{1}H NMR (500 MHz, $CDCl_3$) δ 8.03 (d, 2H, *J* = 7.4 Hz), 7.57 (t, 1H, *J* = 7.4 Hz), 7.44 (t. 2H, *J* = 7.6 Hz), 4.71 (dd, 1H, *J* = 11.0, 3.3 Hz), 4.42 (dd, 1H, *J* = 11.0, 7.6 Hz), 3.73-3.59 (m, 2H), 2.48-2.32 (m, 2H), 2.30-2.19 (m, 1H), 2.18-2.10 (m, 1H), 1.88-1.77 (m, 1H), 1.56 (dd, 1H, *J* = 14.3, 8.6 Hz), 1.37-1.11 (m, 2H), 1.29 (s, 3H), 1.28 (s, 3H), 1.15 (s, 3H), ^{13}C NMR (125 MHz, $CDCl_3$) δ 211.7, 166.8, 133.2, 129.7, 128.4, 64.7, 62.6, 41.7, 38.7, 34.8, 33.2, 32.8, 31.9, 30.6, 30.5, 30.0, 22.5; IR (ATR) ν = 3445, 2958, 2885, 1717, 1668, 1451, 1270 cm^{-1}; HRMS (ESI) *m/z* calcd for $C_{20}H_{27}O_4$ $[(M+H)^+]$ 331.1904, found 331.1920.

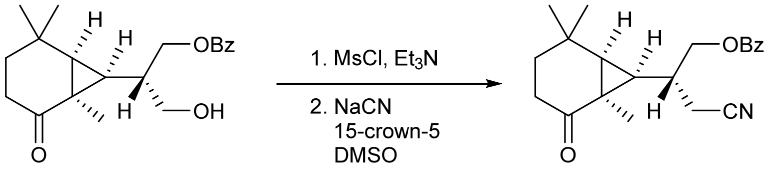

22 **9'**

Compound 9': To a solution of alcohol **22** (4.3 mg, 0.013 mmol) in CH_2Cl_2 (1 mL) were added Et_3N (3.6 μL, 0.026 mmol) and MsCl (2.0 μL, 0.026 mmol) at 0 °C. The solution was stirred at 0 °C for 10 min. The additional Et_3N (7.2 μL, 0.052 mmol) and MsCl (4.0 μL, 0.052 mmol) were added, and the solution was stirred for 25 min. The reaction was quenched with 1 M aq. HCl, and the resultant solution was extracted with $CHCl_3$ three times. The combined organic layers were dried over Na_2SO_4, filtered and concentrated under reduced pressure to give a crude mesylate (5.1 mg, crude)

To a solution of the above crude mesylate (5.1 mg) in DMSO (850 μL) was added 15-crown-5 (12.9 μL, 0.0651 mmol) and NaCN (3.8 mg, 0.0775 mmol) at room temperature. The reaction mixture was stirred at 50 °C for 4.5 h. After dilution with Et_2O and water at 0 °C, the mixture was extracted with Et_2O three times. The combined organic layers were washed with brine, dried over Na_2SO_4, filtered and concentrated under reduced pressure. The residue was purified by silica gel column chromatography (25-40% EtOAc/hexane) to afford **9'** as a colorless oil (4.0 mg, 91%, 2

steps): ^{1}H NMR (500 MHz, $CDCl_3$) δ 7.98 (d, 2H, *J* = 7.7 Hz), 7.53 (t, 1H, *J* = 7.4 Hz), 7.40 (t, 2H, *J* = 7.7 Hz), 4.74 (dd, 1H, *J* = 11.6, 3.6 Hz), 4.19 (dd, 1H, *J* = 11.5, 9.2 Hz), 2.59 (dd, 1H, *J* = 16.9, 3.7 Hz), 2.46 (dd, 1H, *J* = 16.9, 5.7 Hz), 2.39-2.29 (m, 1H), 2.28-2.11 (m, 1H), 2.17 (dd, 1H, *J* = 19.0, 5.3 Hz), 1.69 (dt, 1H, *J* = 20.0, 6.8 Hz), 1.58 (dd, 1H, *J* = 14.0, 8.0 Hz), 1.29 (s, 3H), 1.25 (s, 3H), 1.24-1.09 (m, 2H), 1.14 (s, 3H), ^{13}C NMR (125 MHz, $CDCl_3$) δ 210.6, 166.2, 133.5, 129.7, 129.2, 128.5, 117.1, 65.9, 41.2, 35.2, 33.4, 33.1, 31.4, 30.5, 30.2, 30.1, 22.2, 19.9; IR (ATR) ν = 2962, 2923, 1721, 1681, 1271, 1236, 1091 cm^{-1}; HRMS (ESI) *m/z* calcd for $C_{21}H_{25}NO_3$ [$(M+Na)^+$] 362.1727, found 362.1723.

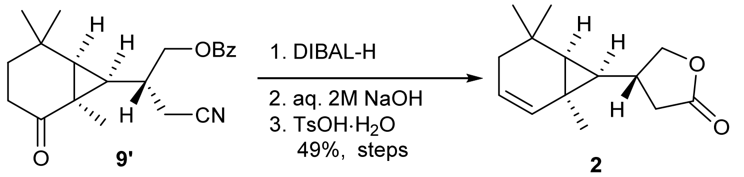

Compound 2: To a solution of nitrile **9'** (120 mg, 0.354 mmol) in THF (3.5 mL) was added DIBAL-H (1.0 M in THF, 1.1 mL) at −20 °C. The solution was stirred at −20 °C for 25 min. Additional DIBAL-H (1.0 M in THF, 400μL) was then added. After 20 min, the reaction was quenched with MeOH and satd. aq. Rochelle salt, and the mixture was diluted with Et_2O. After 1h, the resulting mixture was extracted with Et_2O three times. The combined organic layers were washed with brine, dried over Na_2SO_4, filtered and concentrated under reduced pressure to give a mixture of alcohol and diol (crude).

A solution of the above crude mixture in 2 M aq. NaOH (3.5 mL) was heated to 100 °C. After 5 h, the mixture was cooled to room temperature, and neutralized with 1 M aq. HCl. The mixture was extracted with $CHCl_3$ three times. The combined organic layers were dried over Na_2SO_4, filtered and concentrated under reduced pressure to give a crude lactone **25** (99 mg).

To a solution of the above crude lactone **25** (99 mg) in benzene (7 mL) was added TsOH·H_2O (13 mg, 0.0683 mmol) at room temperature. The solution was stirred at 100 °C with a Dean-Stark trap for 30 min. The reaction was quenched with satd. aq. $NaHCO_3$ at room temperature. The resultant mixture was extracted with EtOAc three times. The combined organic layers were washed with brine, dried over Na_2SO_4, filtered and concentrated under reduced pressure. The residue was purified by silica gel column chromatography (5-40% EtOAc/hexane) to afford **2** as a colorless oil (38 mg, 49%, 3 steps): ^{1}H NMR (500 MHz, $CDCl_3$) δ 5.72 (dd, 1H, *J* = 10.0, 2.6 Hz), 5.57-5.51 (m, 1H), 4.46 (t, 1H, *J* = 8.0 Hz), 4.12 (t, 1H, *J* = 7.9 Hz), 2.55 (dd, 1H, *J* = 17.2, 8.0 Hz), 2.30-2.21 (m, 1H), 2.15 (dd, 1H, *J* = 17.3, 7.6 Hz), 1.85 (dd, 1H, *J* = 18.0, 5.4), 1.53 (d, 1H, *J* =18.0 Hz), 1.27-1.21 (m, 1H), 1.23 (s, 3H), 1.05 (s, 3H), 0.96 (s, 3H), 0.82-0.73 (m, 2H); ^{13}C NMR (125 MHz, $CDCl_3$) δ 177.3, 129.3, 123.7, 73.2, 38.3, 37.7, 36.8, 36.1, 32.6, 31.5, 30.7, 27.6, 25.7, 19.5; IR (ATR) ν = 2974, 2919, 1469, 1236, 1089, 890 cm^{-1}; HRMS (ESI) *m/z* calcd for $C_{14}H_{20}O_2Na$ [$(M+Na)^+$] 243.1356, found

243.1365.

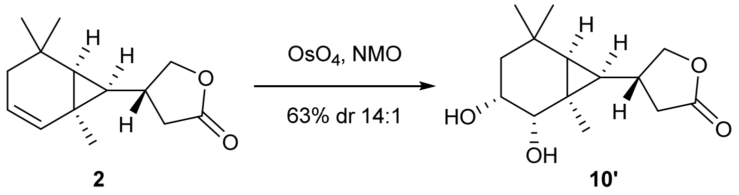

Compound 10’: To a solution of alkene **2** (38 mg, 0.174 mmol) in acetone-H_2O (20:1, 3.5 mL) was added NMO (24 mg, 0.205 mmol) and OsO_4 (*t*-BuOH solution, 0.0393 M, 443 μL). The reaction mixture was stirred at 50 °C for 8 h. Additional OsO_4 (*t*-BuOH solution, 0.0393 M, 222μL) was added, and the mixture was stirred for 2.5 h. After cooling to room temperature, the reaction was quenched with 10% aq. $Na_2S_2O_3$. The mixture was extracted with EtOAc three times. The combined organic layers were washed with brine, dried over Na_2SO_4, filtered and concentrated under reduced pressure. The residue was purified by silica gel column chromatography (20-90% EtOAc/hexane) to afford **10’** as a colorless oil (27 mg, 63%, dr 14:1): ^{1}H NMR (500 MHz, $CDCl_3$) δ 4.49 (dd, 1H, *J* = 8.6, 6.9 Hz), 4.16 (dd, 1H, *J* = 9.0, 5.0 Hz), 3.86 (s, 1H), 3.47 (s, 1H), 2.76 (dd, 1H, *J* = 19.3, 10.2 Hz), 2.38 (d, 1H, *J* = 5.4 Hz), 2.36-2.48 (m, 2H), 2.09 (d, 1H, *J* = 4.9 Hz), 1.72 (dd, 1H, *J* = 14.7, 7.0 Hz), 1.29 (s, 3H), 1.22 (s, 3H), 1.10 (d, 1H, *J* = 16.0 Hz), 0.93 (s, 3H), 0.75 (d, 1H, *J* = 9.5 Hz), 0.68 (t, 1H, *J* = 10.7 Hz); ^{13}C NMR (125 MHz, $CDCl_3$) δ 177.0, 74.2, 70.0, 68.5, 41.3, 36.1, 35.1, 34.0, 32.7, 32.2, 29.8, 28.6, 24.8, 20.3; IR (ATR) ν = 3439, 2977, 2919, 1775, 1469, 1382, 1089, 891 cm^{-1}; HRMS (ESI) *m/z* calcd for $C_{14}H_{22}O_4Na$ [$(M+Na)^+$] 277.1410, found 277.1420.

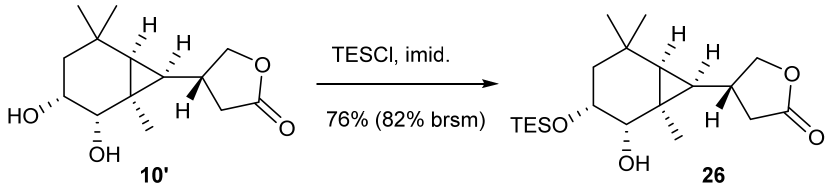

Compound 26: To a solution of diol **10’** (16 mg, 0.0630 mmol) in DMF (1 mL) were added imidazole (11 mg, 0.162 mmol) and TESCl (14 μL, 0.0995 mmol) at room temperature. The solution was stirred at room temperature for 40 min. The reaction was quenched with satd. aq. $NaHCO_3$, and the resultant mixture was extracted with ether three times. The combined organic layers were washed with water and brine, dried over Na_2SO_4, filtered and concentrated under reduced pressure. The residue was purified by silica gel column chromatography (15-90% EtOAc/hexane) to afford **26** as a colorless oil (17 mg, 76%) and **10’** (1.1 mg, 7%) was recovered (brsm 82%): ^{1}H NMR (500 MHz, $CDCl_3$) δ 4.48 (t, 1H, *J* = 7.9 Hz), 4.13 (dd, 1H, *J* = 8.9, 5.2 Hz), 3.82 (t, 1H, *J* = 3.6 Hz), 3.37 (dd, 1H, *J* = 7.0, 2.7 Hz), 2.72 (dd, 1H, *J* = 17.0, 7.9 Hz), 2.51 (d, 1H, *J* = 7.2 Hz), 2.35-2.28 (m, 2H), 1.65 (dd, 1H, *J* = 15.6, 8.4 Hz), 1.25 (s, 3H), 1.19 (s, 3H), 0.98 (t, 9H, *J* = 7.7 Hz),

1.04-0.93 (m, 1H), 0.89 (s, 3H), 0.70 (d, 1H, J = 9.2 Hz), 0.67-0.59 (m, 7H); ^{13}C NMR (125 MHz, $CDCl_3$) δ 176.7, 74.1, 70.8, 68.6, 41.8, 36.1, 35.0, 33.7, 33.0, 32.3, 29.5, 28.9, 24.8, 21.1; IR (ATR) ν = 3537, 2953, 2917, 1779, 1693, 1467, 1379, 1236, 1162, 1089 cm^{-1}; HRMS (FAB) *m/z* calcd for $C_{20}H_{36}O_4SiNa$ [$(M+Na)^+$] 391.2281, found 391.2287.

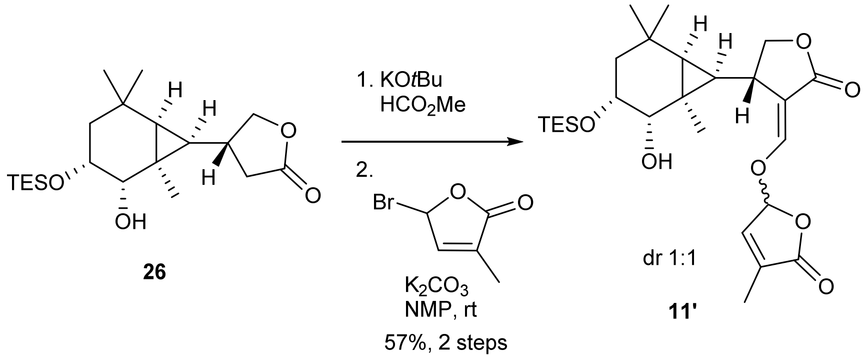

Compound 11': To a solution of alcohol **26** (5.6 mg, 0.0152 mmol) in THF (1 mL) was added HCO_2Me (17 μL, 0.152 mmol) at room temperature. After cooling to −78 °C, KO*t*Bu (1.0 M in THF, 17 μL, 0.017 mmol) was added. The solution was stirred at room temperature for 15 min. The reaction was quenched with satd. aq. NH_4Cl, and the mixture was extracted with EtOAc three times. The combined organic layers were washed with brine, dried over Na_2SO_4, filtered and concentrated under reduced pressure to afford a crude formylated product (5.3 mg) as a colorless oil.

To a solution of the above crude product (5.3 mg) in NMP (500 μL) were added K_2CO_3 (6.1 mg, 0.0499 mmol) and a solution of 5-Bromo-3-methyl-2(5*H*)-furanone (8.5 mg, 0.0480 mmol) in NMP (200 μL) at room temperature. The solution was stirred at room temperature for 15 h. The reaction was quenched with satd. aq. NH_4Cl, and the resultant mixture was extracted with ether six times. The combined organic layers were washed with water twice and brine, dried over Na_2SO_4, filtered and concentrated under reduced pressure. The residue was purified by silica gel column chromatography (15-35% EtOAc/hexane) to afford **11'** as a colorless oil (4.3 mg, 57%, dr 1:1) and **26** (2.0 mg, 36%) was recovered: ^{1}H NMR (500 MHz, C_6D_6) δ 7.53 (s, 1H), 7.51 (s, 1H), 6.41 (s, 1H), 6.09 (s, 1H), 5.37 (s, 1H), 5.20 (s, 1H), 3.94 (t, 1H, J = 8.3 Hz), 3.88 (t, 1H, J = 8.3 Hz), 3.78 (dd, J = 8.6, 3.4 Hz), 3.75-3.71 (m, 2H), 3.67 (t, 1H, J = 2.6 Hz), 3.59 (dd, 1H, J = 8.3, 3.2 Hz), 3.51 (dd, 1H, J =9.2 Hz, 2.9 Hz), 2.35 (s, 2H), 2.20 (d, 1H, J = 8.6 Hz), 2.12 (d, 1H, J = 9.2 Hz), 1.58 (m, 2H), 1.52 (s, 5H), 1.48-1.41 (m, 3H), 1.19 (s, 6H), 1.24-1.17 (m, 1H), 1,14 (s, 3H), 1.10 (s, 3H), 1.01-0.92 (m, 18H), 0.63-0.43 (m, 17H), 0.35 (t, 1H, J = 10.6 Hz), 0.27 (d, 2H, J = 11.5 Hz), 0.22 (m, 2H); ^{13}C NMR (125 MHz, C_6D_6) δ 171.3, 170.6, 170.0, 170.0, 150.8, 142.0, 141.3, 140.5, 135.5, 134.7, 133.9, 100.9, 100.7, 98.3, 98.3, 72.4, 72.2, 71.9, 71.8, 68.2, 42.8, 42.5, 36.2, 33.8, 33.8, 33.7, 33.3, 32.9, 32.9, 31.9, 30.8, 30.5, 28.6, 28.5, 27.8, 25.2, 24.9, 21.5, 21.1, 10.3, 10.3, 10.2, 7.1, 7.1, 5.3; IR (ATR) ν = 3546, 3094, 2953, 2875, 1777, 1677 cm^{-1}; HRMS (FAB) *m/z* calcd for

$C_{26}H_{40}O_7SiNa$ [(M+Na)$^+$] 515.2441, found 515.2444.

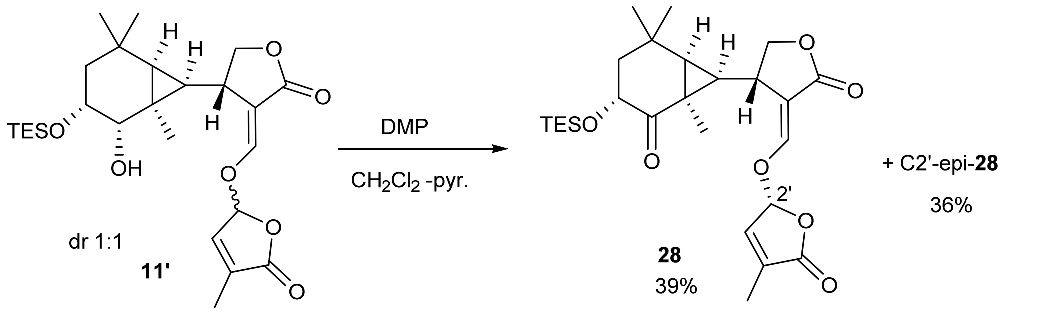

Compound 28 and C2' epi-**28**: To a solution of alcohol **11'** (4.2 mg, 8.53 μmol) in CH_2Cl_2-pyridine (8:1, 900 μL) was added DMP (4.5 mg, 0.0106 mmol) at room temperature. The solution was stirred for 1h. The reaction was quenched with satd. aq. $NaHCO_3$ and 10% aq. $Na_2S_2O_3$, and the resultant mixture was extracted with EtOAc three times. The combined organic layers were washed with water and brine, dried over Na_2SO_4, filtered and concentrated under reduced pressure. The residue was purified by silica gel column chromatography (20-30% EtOAc/hexane) to afford **28** (1.6 mg, 39%) and C2' epi-**28** (1.5 mg, 36%) as a colorless oil: ^{1}H NMR (500 MHz, C_6D_6) δ 7.34 (s, 1H), 6.10 (s, 1H), 5.13 (s, 1H), 3.83 (t, 1H, *J* = 8.7 Hz), 3.66 (dd, 1H, *J* = 7.6, 2.4 Hz), 3.58 (dd, 1H, *J* = 8.6, 5.2 Hz), 2.59 (s,1H), 1.63 (s, 3H), 1.59-1.47 (m, 2H), 1.11 (t, 9H), 1.03 (s, 3H), 0.99-0.88 (m, 1H), 0.97 (s, 3H), 0.84-0.69 (m, 6H), 0.61 (s, 3H), 0.63-0.54 (m, 2H); ^{13}C NMR (125 MHz, C_6D_6) δ 207.2, 170.3, 170.0, 151.6, 142.0, 140.6, 135.4, 110.8, 100.4, 98.3, 71.2, 71.0, 46.1, 43.4, 39.0, 34.8, 33.7, 31.5, 29.9, 29.6. 22.3, 10.4, 10.2, 7.3, 5.6; IR (ATR) ν = 2956, 3873, 1779, 1688, 1456, 1373, 1186, 1011 cm^{-1}; HRMS (FAB) *m/z* calcd for $C_{26}H_{38}O_7SiNa$ [(M+Na)$^+$] 513.2285, found 513.2288;
C2' epi-**28**: ^{1}H NMR (500 MHz, C_6D_6) δ 7.36 (s, 1H), 6.39 (s, 1H), 5.02 (s, 1H), 3.79 (t, 1H, *J* = 8.6 Hz), 3.66 (dd, 1H, *J* = 8.7, 3.9 Hz), 3.60 (dd, 1H, *J* = 7.0, 2.1 Hz), 2.53 (s, 1H), 1.65 (s, 3H), 1.59-1.45 (m, 3H), 1.12-1.04 (m, 9H), 1.00 (s, 3H), 0.78-0.55 (m, 7H), 0.64 (s, 3H), 0.41 (s, 3H) ^{13}C NMR (100 MHz, C_6D_6) δ 207.2, 170.1, 169.8, 151.9, 141.2, 134.7, 110.9, 100.5,71.3, 70.8, 46.0, 43.5, 39.8, 34.4, 33.7, 31.4, 29.9, 29.7, 22.8, 10.4, 7.2, 5.5; IR (ATR) ν = 2974, 2919, 2022, 1782, 1747, 1688, 1236, 1089 cm^{-1}; HRMS (FAB) *m/z* calcd for $C_{26}H_{38}O_7SiNa$ [(M+Na)$^+$] 513.2285, found 513.2270.

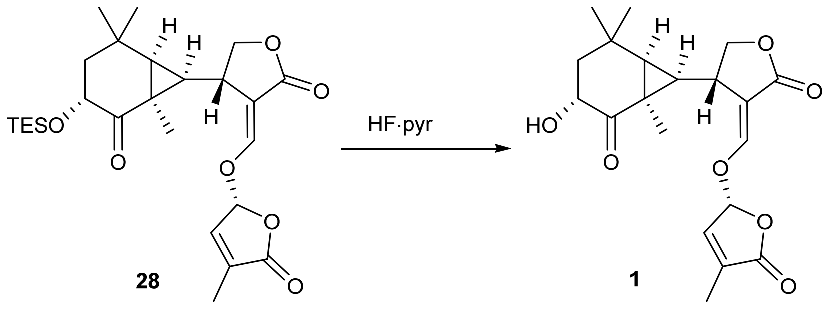

Compound 1: To a solution of ketone **28** (1.9 mg, 3.88 μmol) in THF (400 μL) was added HF·pyridine-pyridine-THF solution (1:3:16, 25 μL) at room temperature. The solution was stirred at 50 °C for 45 min. An additional HF·pyridine-pyridine-THF (1:3:16, 50 μL) was then added. After 1 h, the mixture was cooled to 0 °C. The reaction was quenched with satd. aq. $NaHCO_3$, and the resultant mixture was extracted with EtOAc three times. The combined organic layers were washed with brine, dried over Na_2SO_4, filtered and concentrated under reduced pressure. The residue was purified by silica gel column chromatography (45-65% EtOAc/hexane) to afford avenaol (**1**) as a colorless oil (1.3 mg, 97%): ^{1}H NMR (500 MHz, C_6D_6) δ 7.30 (s, 1H), 5.77 (s, 1H), 4.90 (s, 1H), 3.89 (s, 1H), 3.78 (s, 1H), 3.78 (t, 1H, *J* = 8.6 Hz), 3.62 (t, 1H, *J* = 6.4 Hz), 3.53 (dd, 1H, *J* = 8.4, 3.9 Hz), 2.66 (t, 1H, *J* = 9.3 Hz), 1.66 (s, 3H), 1.60 (d. 2H, *J* = 6.6 Hz), 1.01 (s, 3H), 0.75 (s, 3H), 0.68-0.58 (m, 2H); ^{13}C NMR (125 MHz, C_6D_6) δ 210.4, 169.9, 169.7, 151.9, 139.4, 135.5, 110.4, 100.3, 70.8, 69.9, 46.3, 46.2, 40.1, 33.8, 33.4, 31.7, 29.6, 28.6, 21.3, 10.2 ; IR (ATR) ν = 3461, 2959, 2855, 1780, 1751, 1682, 1341, 1186 cm^{-1}; HRMS (FAB) *m/z* calcd for $C_{20}H_{24}O_7Na$ [$(M+Na)^+$] 399.1420, found 399.1423.

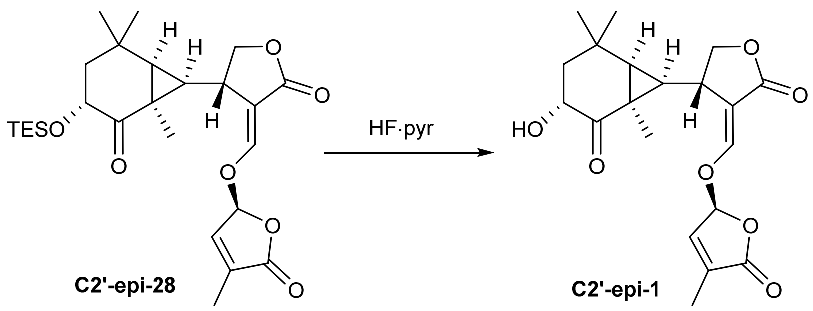

Compound C2' epi-1: To a solution of ketone **C2'-epi-28** (5.3 mg, 10.8 μmol) in THF (500 μL) was HF·pyridine-pyridine-THF solution (1:3:16, 76 μL) at room temperature. The solution was stirred at 50 °C for 45 min. An additional HF·pyridine-pyridine-THF (1:3:16, 25 μL) was then added.. After 1 h, the mixture was cooled to 0 °C. The reaction was quenched with satd. aq. $NaHCO_3$, and the resultant solution was extracted with EtOAc three times. The combined organic layers were washed with brine, dried over Na_2SO_4, filtered and concentrated under reduced pressure. The residue was purified by silica gel column chromatography (45-65% EtOAc/hexane) to afford **C2' epi-1** as a

colorless oil (0.6 mg, 11%). For X-ray crystallography, C2' epi-1 was recrystallized from ethyl acetate: ^{1}H NMR (500 MHz, C_6D_6) δ 7.27 (d, *J* = 1.7 Hz), 6.17 (s, 1H), 4.89 (s, 1H), 3.75 (s, 1H), 3.71 (t, 1H, *J* = 8.6 Hz), 3.62 (t, 1H, *J* = 6.9 Hz), 3.56 (dd, 1H, *J* = 8.9, 3.2 Hz), 2.56 (t, 1H, *J* = 9.7 Hz), 1.55 (d, 2H, *J* = 6.9 Hz), 1.53 (s, 3H), 1.09 (s, 3H), 0.78 (s, 3H), 0.76-0.68 (m, 2H), 0.49 (s, 3H); ^{13}C NMR (125 MHz, C_6D_6) δ 211.4, 170.2, 170.0, 152.0, 140.9, 135.0, 110.4, 100.6, 71.4, 70.3, 46.8, 46.8, 41.1, 34.0, 33.6, 32.4, 30.3, 29.9, 28.8, 22.1, 10.4 ; IR (ATR) ν = 3473, 2961, 2922, 2026, 1781, 1752, 1088, 890 cm^{-1}; HRMS (FAB) *m/z* calcd for $C_{20}H_{24}O_7Na$ $[(M+Na)^+]$ 399.1420, found 399.1433.

Experimental details of X-ray crystallography of C2'-epi avenaol

A. Crystal Data

Empirical Formula C20H24O7

Formula Weight 376.41

Crystal Color, Habit colorless, nONE

Crystal Dimensions not described

Crystal System monoclinic

Lattice Type Primitive

No. of Reflections Used for Unit
Cell Determination (2q range) 5633 (8.6 - 145.0o)

Omega Scan Peak Width
at Half-height 0.00o

Lattice Parameters a = 7.8120(3) Å
b = 19.6845(5) Å
c = 12.1028(3) Å
b = 99.228(3) o
V = 1837.03(10) Å3

Space Group P21/c (#14)

Z value 4

Dcalc 1.361 g/cm3

F000 800.00

m(CuKa) 8.608 cm-1

B. Intensity Measurements

Diffractometer

Radiation CuKa (l = 1.54187 Å)
graphite monochromated

Take-off Angle 2.8o

Detector Aperture 2.0 - 2.5 mm horizontal
2.0 mm vertical

Crystal to Detector Distance 21 mm

Temperature 23.0oC

Scan Type w-2q

Scan Rate 0.0o/min (in w) (up to 0 scans)

Scan Width (0.00 + 0.00 tan q)o

2qmax 146.6o

No. of Reflections Measured Total: 17343
Unique: 3478 (Rint = 0.0641)

Corrections Lorentz-polarization
Absorption
(trans. factors: 0.598 - 1.000)

C. Structure Solution and Refinement

Structure Solution Direct Methods (SIR2011)

Refinement Full-matrix least-squares on F2

Function Minimized S w (Fo2 - Fc2)2

Least Squares Weights w = 1/ [s2(Fo2) + (0.0596 . P)2
+ 1.1269 . P]
where P = (Max(Fo2,0) + 2Fc2)/3

2qmax cutoff 146.6o

Anomalous Dispersion All non-hydrogen atoms

No. Observations (All reflections) 3478

No. Variables 244

Reflection/Parameter Ratio 14.25

Residuals: R1 (I>2.00s(I)) 0.0507

Residuals: R (All reflections) 0.0718

Residuals: wR2 (All reflections) 0.1494

Goodness of Fit Indicator 1.083

Max Shift/Error in Final Cycle 0.000

Maximum peak in Final Diff. Map 0.24 e-/Å3

Minimum peak in Final Diff. Map -0.23 e-/Å3

Table 1. Atomic coordinates and Biso/Beq

atom	x	y	z	Beq
O1	0.7595(2)	0.07108(8)	0.70816(12)	2.71(3)
O2	0.8045(2)	-0.02489(8)	0.81978(12)	2.72(3)
O3	0.6906(3)	-0.07375(9)	0.95861(14)	3.37(4)
O4	0.2222(2)	0.12131(9)	0.81549(14)	3.32(4)
O5	0.5332(2)	0.17897(10)	0.85148(14)	3.33(4)
O6	0.5744(3)	0.07304(9)	0.32591(13)	3.33(4)
O7	0.8317(3)	0.02342(10)	0.37385(14)	3.84(4)
C1	0.6782(3)	0.07141(11)	0.51435(18)	2.49(4)
C2	0.7537(3)	-0.02432(12)	0.92304(18)	2.59(4)
C3	0.3115(3)	0.12711(13)	0.72281(19)	2.71(4)
C4	0.8622(4)	0.08039(13)	0.90263(19)	2.78(4)
C5	0.8712(3)	0.04072(12)	0.79916(18)	2.61(4)
C6	0.7896(4)	0.05303(12)	0.60421(18)	2.63(4)
C7	0.4703(3)	0.17257(12)	0.75281(19)	2.61(4)
C8	0.7099(4)	0.05289(12)	0.40210(19)	2.95(5)
C9	0.7914(3)	0.04331(12)	0.97463(18)	2.62(4)
C10	0.5351(3)	0.18227(12)	0.54564(19)	2.59(4)
C11	0.5405(3)	0.21045(12)	0.6641(2)	2.55(4)
C12	0.1821(4)	0.14986(13)	0.6212(2)	2.94(5)
C13	0.2124(3)	0.22044(13)	0.5746(2)	2.77(5)
C14	0.7499(4)	0.06013(14)	1.0873(2)	3.29(5)
C15	0.4020(3)	0.23351(12)	0.5669(2)	2.69(4)
C16	0.5102(3)	0.10935(12)	0.50556(18)	2.52(4)
C17	0.4459(4)	0.10740(14)	0.37896(19)	3.06(5)
C18	0.1019(4)	0.22780(15)	0.4597(2)	3.57(5)
C19	0.1599(4)	0.27491(14)	0.6536(2)	3.38(5)
C20	0.6882(4)	0.25732(14)	0.7077(2)	3.38(5)

Beq = 8/3 p2(U11(aa*)2 + U22(bb*)2 + U33(cc*)2 + 2U12(aa*bb*)cos g + 2U13(aa*cc*)cos b + 2U23(bb*cc*)cos a)

Table 2. Atomic coordinates and Biso involving hydrogen atoms

atom	x	y	z	Biso
H4	0.29158	0.11260	0.87200	3.983
H3	0.35254	0.08170	0.70669	3.252
H4A	0.90071	0.12487	0.91498	3.337
H5	0.99050	0.03779	0.78400	3.127
H6	0.88769	0.02794	0.59620	3.156
H10	0.62431	0.20430	0.50969	3.112
H12A	0.06684	0.14898	0.64138	3.533
H12B	0.18390	0.11686	0.56189	3.533
H14A	0.76715	0.02067	1.13437	3.945
H14B	0.63126	0.07453	1.08022	3.945
H14C	0.82456	0.09603	1.11992	3.945
H15	0.42329	0.27902	0.53922	3.225
H16	0.42923	0.08548	0.54608	3.026
H17A	0.33616	0.08340	0.36353	3.669
H17B	0.42846	0.15325	0.34999	3.669
H18A	0.13346	0.19328	0.41070	4.284
H18B	0.12088	0.27179	0.42964	4.284
H18C	-0.01830	0.22299	0.46637	4.284
H19A	0.21889	0.26673	0.72833	4.058
H19B	0.03686	0.27308	0.65273	4.058
H19C	0.19109	0.31898	0.62931	4.058
H20A	0.71869	0.28370	0.64705	4.058
H20B	0.78656	0.23101	0.74103	4.058
H20C	0.65364	0.28714	0.76290	4.058

Table 3. Anisotropic displacement parameters

atom	U11	U22	U33	U12	U13	U23
O1	0.0405(11)	0.0397(9)	0.0237(7)	0.0026(8)	0.0083(7)	0.0012(6)
O2	0.0438(11)	0.0329(9)	0.0278(8)	-0.0025(8)	0.0098(7)	-0.0005(6)
O3	0.0497(13)	0.0406(10)	0.0392(9)	-0.0082(8)	0.0115(8)	0.0064(8)
O4	0.0430(12)	0.0544(11)	0.0320(8)	-0.0051(9)	0.0161(8)	0.0084(8)
O5	0.0408(12)	0.0547(11)	0.0312(8)	0.0007(9)	0.0066(8)	-0.0069(8)
O6	0.0525(13)	0.0476(11)	0.0272(8)	0.0107(9)	0.0091(8)	-0.0001(7)
O7	0.0606(14)	0.0520(12)	0.0359(9)	0.0183(10)	0.0152(9)	-0.0008(8)
C1	0.0422(16)	0.0269(11)	0.0267(11)	-0.0009(10)	0.0095(10)	0.0005(8)
C2	0.0350(15)	0.0366(13)	0.0273(11)	-0.0009(11)	0.0069(10)	0.0034(9)
C3	0.0369(15)	0.0379(13)	0.0310(11)	-0.0060(11)	0.0140(10)	0.0038(10)
C4	0.0382(16)	0.0355(13)	0.0314(11)	-0.0039(11)	0.0036(10)	-0.0016(9)
C5	0.0321(15)	0.0360(13)	0.0312(11)	-0.0044(10)	0.0057(10)	0.0051(9)
C6	0.0455(16)	0.0295(12)	0.0270(11)	0.0004(11)	0.0124(10)	0.0001(9)
C7	0.0328(15)	0.0349(13)	0.0335(11)	0.0055(10)	0.0109(10)	-0.0028(9)
C8	0.0511(18)	0.0337(13)	0.0287(11)	0.0061(12)	0.0104(11)	0.0015(9)
C9	0.0343(15)	0.0361(13)	0.0291(11)	0.0028(10)	0.0053(10)	-0.0006(9)

C10	0.0331(15)	0.0330(12)	0.0354(12)	-0.0030(10)	0.0143(10)	0.0030(9)
C11	0.0293(14)	0.0313(12)	0.0385(12)	-0.0041(10)	0.0118(10)	-0.0040(10)
C12	0.0352(16)	0.0433(14)	0.0353(12)	-0.0082(11)	0.0117(11)	0.0042(10)
C13	0.0319(15)	0.0385(14)	0.0367(12)	-0.0004(11)	0.0115(11)	0.0048(10)
C14	0.0501(18)	0.0455(15)	0.0306(12)	0.0068(13)	0.0102(11)	-0.0010(10)
C15	0.0340(15)	0.0322(12)	0.0391(12)	-0.0015(10)	0.0153(11)	0.0039(10)
C16	0.0388(15)	0.0310(12)	0.0280(11)	-0.0037(10)	0.0114(10)	0.0012(9)
C17	0.0430(17)	0.0425(14)	0.0323(12)	0.0018(12)	0.0109(11)	-0.0006(10)
C18	0.0406(17)	0.0560(17)	0.0404(14)	0.0016(13)	0.0102(12)	0.0110(12)
C19	0.0372(16)	0.0451(15)	0.0496(14)	0.0047(12)	0.0174(12)	0.0027(12)
C20	0.0354(16)	0.0401(14)	0.0561(16)	-0.0046(12)	0.0167(13)	-0.0126(12)

The general temperature factor expression: exp(-2p2(a*2U11h2 + b*2U22k2 + c*2U33l2 + 2a*b*U12hk + 2a*c*U13hl + 2b*c*U23kl))

Table 4. Bond lengths (Å)

atom	atom	distance	atom	atom	distance
O1	C5	1.423(3)	O1	C6	1.363(3)
O2	C2	1.371(3)	O2	C5	1.430(3)
O3	C2	1.201(3)	O4	C3	1.417(3)
O5	C7	1.223(3)	O6	C8	1.347(3)
O6	C17	1.444(3)	O7	C8	1.210(4)
C1	C6	1.329(3)	C1	C8	1.465(3)
C1	C16	1.499(4)	C2	C9	1.480(3)
C3	C7	1.526(4)	C3	C12	1.528(3)
C4	C5	1.487(3)	C4	C9	1.324(4)
C7	C11	1.483(4)	C9	C14	1.489(4)
C10	C11	1.531(3)	C10	C15	1.500(4)
C10	C16	1.518(3)	C11	C15	1.533(3)
C11	C20	1.505(4)	C12	C13	1.532(4)
C13	C15	1.521(4)	C13	C18	1.521(3)
C13	C19	1.536(4)	C16	C17	1.534(3)

Table 5. Bond lengths involving hydrogens (Å)

atom	atom	distance	atom	atom	distance
O4	H4	0.820	C3	H3	0.980
C4	H4A	0.930	C5	H5	0.980
C6	H6	0.930	C10	H10	0.980
C12	H12A	0.970	C12	H12B	0.970
C14	H14A	0.960	C14	H14B	0.960
C14	H14C	0.960	C15	H15	0.980
C16	H16	0.980	C17	H17A	0.970
C17	H17B	0.970	C18	H18A	0.960
C18	H18B	0.960	C18	H18C	0.960
C19	H19A	0.960	C19	H19B	0.960
C19	H19C	0.960	C20	H20A	0.960
C20	H20B	0.960	C20	H20C	0.960

Table 6. Bond angles (o)

atom	atom	atom	angle	atom	atom	atom	angle
C5	O1	C6	115.45(19)	C2	O2	C5	108.59(17)
C8	O6	C17	111.07(18)	C6	C1	C8	120.4(2)
C6	C1	C16	130.1(2)	C8	C1	C16	109.51(19)
O2	C2	O3	121.0(2)	O2	C2	C9	109.0(2)
O3	C2	C9	130.0(2)	O4	C3	C7	110.10(19)
O4	C3	C12	108.3(2)	C7	C3	C12	115.3(2)
C5	C4	C9	110.2(2)	O1	C5	O2	108.61(18)
O1	C5	C4	109.0(2)	O2	C5	C4	105.05(19)
O1	C6	C1	119.8(2)	O5	C7	C3	118.5(2)
O5	C7	C11	121.2(2)	C3	C7	C11	120.24(19)
O6	C8	O7	121.0(2)	O6	C8	C1	109.3(2)
O7	C8	C1	129.7(2)	C2	C9	C4	107.1(2)
C2	C9	C14	121.9(2)	C4	C9	C14	131.0(2)
C11	C10	C15	60.75(16)	C11	C10	C16	128.7(2)
C15	C10	C16	129.3(2)	C7	C11	C10	122.5(2)
C7	C11	C15	114.0(2)	C7	C11	C20	113.9(2)
C10	C11	C15	58.61(16)	C10	C11	C20	117.1(2)
C15	C11	C20	119.6(2)	C3	C12	C13	116.1(2)
C12	C13	C15	112.7(2)	C12	C13	C18	108.9(2)
C12	C13	C19	109.3(2)	C15	C13	C18	110.1(2)
C15	C13	C19	106.3(2)	C18	C13	C19	109.5(2)
C10	C15	C11	60.63(16)	C10	C15	C13	127.2(2)
C11	C15	C13	118.5(2)	C1	C16	C10	112.5(2)
C1	C16	C17	101.6(2)	C10	C16	C17	110.33(19)
O6	C17	C16	108.4(2)				

Table 7. Bond angles involving hydrogens (o)

atom	atom	atom	angle	atom	atom	atom	angle
C3	O4	H4	109.5	O4	C3	H3	107.6
C7	C3	H3	107.6	C12	C3	H3	107.6
C5	C4	H4A	124.9	C9	C4	H4A	124.9
O1	C5	H5	111.3	O2	C5	H5	111.3
C4	C5	H5	111.3	O1	C6	H6	120.1
C1	C6	H6	120.1	C11	C10	H10	109.9
C15	C10	H10	109.9	C16	C10	H10	109.9
C3	C12	H12A	108.2	C3	C12	H12B	108.2
C13	C12	H12A	108.3	C13	C12	H12B	108.3
H12A	C12	H12B	107.4	C9	C14	H14A	109.5
C9	C14	H14B	109.5	C9	C14	H14C	109.5
H14A	C14	H14B	109.5	H14A	C14	H14C	109.5
H14B	C14	H14C	109.5	C10	C15	H15	113.3
C11	C15	H15	113.3	C13	C15	H15	113.3
C1	C16	H16	110.7	C10	C16	H16	110.7
C17	C16	H16	110.7	O6	C17	H17A	110.0
O6	C17	H17B	110.0	C16	C17	H17A	110.0
C16	C17	H17B	110.0	H17A	C17	H17B	108.4
C13	C18	H18A	109.5	C13	C18	H18B	109.5
C13	C18	H18C	109.5	H18A	C18	H18B	109.5
H18A	C18	H18C	109.5	H18B	C18	H18C	109.5
C13	C19	H19A	109.5	C13	C19	H19B	109.5
C13	C19	H19C	109.5	H19A	C19	H19B	109.5
H19A	C19	H19C	109.5	H19B	C19	H19C	109.5
C11	C20	H20A	109.5	C11	C20	H20B	109.5
C11	C20	H20C	109.5	H20A	C20	H20B	109.5
H20A	C20	H20C	109.5	H20B	C20	H20C	109.5

Table 8. Torsion Angles(o)

(Those having bond angles > 160 or < 20 degrees are excluded.)

atom1	atom2	atom3	atom4	angle	atom1	atom2	atom3	atom4	angle
C5	O1	C6	C1	171.04(19)	C6	O1	C5	O2	-84.0(2)
C6	O1	C5	C4	162.10(18)	C2	O2	C5	O1	-114.28(18)
C2	O2	C5	C4	2.2(2)	C5	O2	C2	O3	178.9(2)
C5	O2	C2	C9	-1.4(2)	C8	O6	C17	C16	0.4(3)
C17	O6	C8	O7	-179.6(2)	C17	O6	C8	C1	1.4(3)
C6	C1	C8	O6	175.9(2)	C6	C1	C8	O7	-2.9(4)
C8	C1	C6	O1	179.4(2)	C6	C1	C16	C10	66.3(3)
C6	C1	C16	C17	-175.7(2)	C16	C1	C6	O1	-2.3(4)
C8	C1	C16	C10	-115.27(19)	C8	C1	C16	C17	2.7(2)
C16	C1	C8	O6	-2.7(3)	C16	C1	C8	O7	178.5(2)
O2	C2	C9	C4	-0.1(3)	O2	C2	C9	C14	179.61(18)
O3	C2	C9	C4	179.6(2)	O3	C2	C9	C14	-0.8(4)
O4	C3	C7	O5	23.5(3)	O4	C3	C7	C11	-153.59(18)
O4	C3	C12	C13	114.3(2)	C7	C3	C12	C13	-9.6(3)
C12	C3	C7	O5	146.4(2)	C12	C3	C7	C11	-30.7(3)

C5	C4	C9	C2	1.5(3)	C5	C4	C9	C14
	-178.2(2)							
C9	C4	C5	O1	113.9(2)	C9	C4	C5	O2
	-2.3(3)							
O5	C7	C11	C10	149.2(2)	O5	C7	C11	C15
	-143.9(2)							
O5	C7	C11	C20	-1.7(3)	C3	C7	C11	C10
	-33.8(3)							
C3	C7	C11	C15	33.1(3)	C3	C7	C11	C20
	175.34(19)							
C11	C10	C15	C11	0.00(11)	C11	C10	C15	C13
	-105.1(2)							
C15	C10	C11	C7	100.2(2)	C15	C10	C11	C15
	-0.00(11)							
C15	C10	C11	C20	-109.8(2)		C11	C10	C16
	C1	-88.6(3)						
C11	C10	C16	C17	158.7(2)	C16	C10	C11	C7
	-18.7(4)							
C16	C10	C11	C15	-118.8(3)		C16	C10	C11
	C20	131.4(2)						
C15	C10	C16	C1	-169.8(2)		C15	C10	C16
	C17	77.5(3)						
C16	C10	C15	C11	117.8(3)	C16	C10	C15	C13
	12.7(4)							
C7	C11	C15	C10	-114.7(2)		C7	C11	C15
	C13	4.3(3)						
C10	C11	C15	C10	-0.00(11)		C10	C11	C15
	C13	119.0(2)						
C20	C11	C15	C10	105.4(2)	C20	C11	C15	C13
	-135.6(2)							
C3	C12	C13	C15	44.0(3)	C3	C12	C13	C18
	166.4(2)							
C3	C12	C13	C19	-73.9(3)	C12	C13	C15	C10
	31.3(3)							
C12	C13	C15	C11	-41.9(3)	C18	C13	C15	C10
	-90.5(3)							

C18	C13	C15	C11	-163.74(19)	C19	C13	C15	C10	151.0(2)
C19	C13	C15	C11	77.8(2)	C1	C16	C17	O6	-1.9(2)
C10	C16	C17	O6	117.6(2)					

Table 9. Possible hydrogen bonds

Donor	H	Acceptor	D...A	D-H	H...A	D-H...A	
O4	H4	O3[1]	2.867(2)	0.82	2.17	142.65	
O4	H4	O5	2.654(3)	0.82	2.34	103.38	intramol.

Symmetry Operators:

(1) -X+1,-Y,-Z+2

Table 10. Intramolecular contacts less than 3.60 Å

atom	atom	distance	atom	atom	distance
O1	O5	3.410(3)	O1	C2	3.214(3)
O1	C7	3.127(3)	O1	C9	3.241(3)
O1	C10	3.260(3)	O1	C11	3.232(3)
O1	C16	2.973(3)	O2	C6	3.013(3)
O3	C4	3.428(3)	O3	C5	3.416(3)
O3	C14	3.058(3)	O4	O5	2.654(3)
O4	C13	3.499(3)	O4	C19	3.593(3)
O5	C4	3.201(3)	O5	C9	3.530(3)
O5	C15	3.596(3)	O5	C20	2.747(3)
O6	C6	3.537(3)	O6	C10	3.472(3)
O7	C6	2.918(3)	O7	C17	3.447(4)
C1	C5	3.585(3)	C1	C11	3.544(3)
C3	C10	3.167(4)	C3	C15	2.979(4)
C3	C16	3.279(4)	C3	C19	3.201(4)
C6	C10	3.236(3)	C7	C13	2.865(3)
C7	C16	3.301(3)	C7	C19	3.232(4)
C8	C10	3.483(4)	C10	C12	3.108(4)
C10	C18	3.494(4)	C11	C12	3.011(4)
C11	C19	3.217(4)	C12	C16	3.212(4)
C13	C16	3.393(4)	C15	C17	3.421(4)

Table 11. Intramolecular contacts less than 3.60 Å involving hydrogens

atom	atom	distance	atom	atom	distance
O1	H3	3.183	O1	H4A	2.778
O1	H10	3.598	O1	H16	2.993
O1	H20B	3.176	O2	H4A	3.210
O2	H6	3.064	O3	H14A	2.816
O3	H14B	3.335	O4	H12A	2.321
O4	H12B	3.037	O4	H19A	3.049
O5	H4	2.341	O5	H3	2.817
O5	H4A	3.043	O5	H14B	3.435
O5	H19A	3.172	O5	H20B	2.757
O5	H20C	2.625	O6	H10	3.391
O6	H16	3.069	O7	H6	2.658
C1	H10	2.649	C1	H17A	2.994
C1	H17B	3.019	C2	H4A	3.161
C2	H5	2.958	C2	H14A	2.693
C2	H14B	2.984	C2	H14C	3.343
C3	H16	2.593	C3	H19A	2.845
C4	H14A	3.235	C4	H14B	3.022
C4	H14C	2.710	C4	H20B	3.549
C5	H6	2.493	C6	H5	2.489
C6	H10	3.372	C6	H16	2.862
C7	H4	2.464	C7	H10	3.410
C7	H12A	3.257	C7	H12B	3.145
C7	H15	3.302	C7	H16	3.007
C7	H19A	2.682	C7	H20A	3.314
C7	H20B	2.749	C7	H20C	2.664
C8	H6	2.577	C8	H10	3.362
C8	H16	3.079	C8	H17A	2.943
C8	H17B	2.948	C9	H5	2.984
C10	H3	3.262	C10	H12B	3.065
C10	H17A	3.156	C10	H17B	2.448
C10	H18A	3.304	C10	H20A	2.643
C10	H20B	2.980	C10	H20C	3.354

C11	H3	3.015	C11	H12B	3.403
C11	H16	2.907	C11	H19A	2.962
C11	H19C	3.439	C12	H4	3.106
C12	H15	3.406	C12	H16	2.593
C12	H18A	2.656	C12	H18B	3.318

Table 11. Intramolecular contacts less than 3.60 Å involving hydrogens (continued)

atom	atom	distance	atom	atom	distance
C12	H18C	2.663	C12	H19A	2.634
C12	H19B	2.730	C12	H19C	3.331
C13	H3	3.264	C13	H10	3.447
C13	H16	3.199	C14	H4A	2.855
C15	H3	3.486	C15	H12A	3.346
C15	H12B	2.854	C15	H16	2.935
C15	H17B	3.099	C15	H18A	2.708
C15	H18B	2.642	C15	H18C	3.320
C15	H19A	2.681	C15	H19B	3.282
C15	H19C	2.552	C15	H20A	2.696
C15	H20B	3.382	C15	H20C	3.018
C16	H3	2.949	C16	H6	3.381
C16	H12B	2.746	C16	H15	3.445
C16	H18A	3.409	C17	H10	2.716
C17	H12B	3.252	C17	H18A	3.043
C18	H12A	2.740	C18	H12B	2.540
C18	H15	2.733	C18	H17B	3.393
C18	H19A	3.322	C18	H19B	2.625
C18	H19C	2.731	C19	H12A	2.581
C19	H12B	3.319	C19	H15	2.659
C19	H18A	3.328	C19	H18B	2.680
C19	H18C	2.666	C20	H10	2.588
C20	H15	2.697	H4	H3	2.215
H4	H12A	3.133	H4	H14B	3.436
H4	H19A	3.498	H3	H12A	2.607
H3	H12B	2.132	H3	H16	2.125
H4A	H5	2.510	H4A	H14B	3.281
H4A	H14C	2.702	H4A	H20B	2.998
H5	H6	2.294	H10	H15	2.222
H10	H16	2.864	H10	H17A	3.547
H10	H17B	2.477	H10	H20A	2.315
H10	H20B	2.930	H10	H20C	3.446

H12A	H16	3.458	H12A	H18A	3.048
H12A	H18B	3.597	H12A	H18C	2.569
H12A	H19A	2.737	H12A	H19B	2.460
H12A	H19C	3.494	H12B	H16	2.052
H12B	H17A	2.920	H12B	H17B	3.509

Table 11. Intramolecular contacts less than 3.60 Å involving hydrogens (continued)

atom	atom	distance	atom	atom	distance
H12B	H18A	2.353	H12B	H18B	3.443
H12B	H18C	2.759	H12B	H19A	3.558
H12B	H19B	3.520	H15	H17B	3.377
H15	H18A	3.044	H15	H18B	2.521
H15	H18C	3.594	H15	H19A	3.003
H15	H19B	3.513	H15	H19C	2.394
H15	H20A	2.464	H15	H20B	3.562
H15	H20C	3.005	H16	H17A	2.213
H16	H17B	2.722	H16	H18A	3.363
H17A	H18A	2.793	H17B	H18A	2.649
H17B	H18B	3.590	H18A	H19B	3.511
H18A	H19C	3.598	H18B	H19A	3.574
H18B	H19B	2.879	H18B	H19C	2.565
H18C	H19A	3.513	H18C	H19B	2.436
H18C	H19C	3.017	H19A	H20C	3.379

Table 12. Intermolecular contacts less than 3.60 Å

atom	atom	distance	atom	atom	distance
O2	O6^{1}	3.331(2)	O2	C14^{2}	3.551(3)
O2	C17^{1}	3.277(3)	O3	O4^{3}	2.867(2)
O3	C14^{3}	3.407(4)	O3	C19^{4}	3.549(3)
O4	O3^{3}	2.867(2)	O4	C4^{5}	3.259(3)
O4	C5^{5}	3.146(3)	O5	C15^{6}	3.417(3)
O6	O2^{1}	3.331(2)	O6	C14^{7}	3.399(3)
O7	C3^{1}	3.313(3)	O7	C5^{8}	3.595(3)
O7	C6^{8}	3.292(3)	O7	C12^{1}	3.413(3)
O7	C14^{7}	3.500(3)	C2	C4^{2}	3.556(3)
C2	C9^{2}	3.590(4)	C3	O7^{1}	3.313(3)
C4	O4^{9}	3.259(3)	C4	C2^{2}	3.556(3)
C5	O4^{9}	3.146(3)	C5	O7^{8}	3.595(3)
C6	O7^{8}	3.292(3)	C6	C12^{9}	3.588(4)
C9	C2^{2}	3.590(4)	C12	O7^{1}	3.413(3)
C12	C6^{5}	3.588(4)	C14	O2^{2}	3.551(3)
C14	O3^{3}	3.407(4)	C14	O6^{10}	3.399(3)
C14	O7^{10}	3.500(3)	C15	O5^{11}	3.417(3)
C17	O2^{1}	3.277(3)	C19	O3^{12}	3.549(3)

Symmetry Operators:

(1)	-X+1,-Y,-Z+1	(2)	-X+2,-Y,-Z+2
(3)	-X+1,-Y,-Z+2	(4)	-X+1,Y+1/2-1,-Z+1/2+1
(5)	X-1,Y,Z	(6)	X,-Y+1,Z+1
(7)	X,Y,Z-1	(8)	-X+2,-Y,-Z+1
(9)	X+1,Y,Z	(10)	X,Y,Z+1
(11)	X,-Y+1,Z	(12)	-X+1,Y+1/2,-Z+1/2+1

Table 13. Intermolecular contacts less than 3.60 Å involving hydrogens

atom	atom	distance	atom	atom	distance
O1	H12A[1]	3.065	O1	H17A[2]	3.217
O2	H14A[3]	3.304	O2	H14C[3]	3.196
O2	H17A[2]	2.583	O2	H17B[2]	3.566
O2	H19C[4]	3.134	O3	H4[5]	2.172
O3	H4A[3]	3.461	O3	H14B[5]	2.482
O3	H15[4]	3.033	O3	H18B[4]	3.550
O3	H19C[4]	2.600	O4	H4A[6]	2.955
O4	H5[6]	2.429	O4	H14A[5]	2.858
O4	H15[7]	3.505	O4	H18B[7]	2.704
O5	H10[7]	3.003	O5	H15[7]	2.685
O5	H17B[7]	3.402	O6	H3[2]	3.136
O6	H14A[8]	3.134	O6	H14B[8]	3.076
O6	H14C[8]	3.435	O6	H16[2]	3.486
O6	H20C[9]	2.948	O7	H3[2]	2.618
O7	H5[10]	2.808	O7	H6[10]	2.389
O7	H12A[2]	3.497	O7	H12B[2]	2.877
O7	H14A[8]	2.862	O7	H14C[8]	3.382
O7	H16[2]	3.213	C1	H12A[1]	3.523
C1	H16[2]	3.253	C1	H17A[2]	3.397
C2	H4[5]	3.095	C2	H14B[5]	3.160
C2	H19C[4]	3.192	C3	H5[6]	3.244
C3	H14A[5]	3.488	C4	H4[1]	3.491
C4	H14A[3]	3.600	C4	H18B[11]	3.528
C5	H4[1]	3.555	C5	H12A[1]	3.385
C5	H14A[3]	3.059	C5	H17A[2]	3.383
C6	H12A[1]	2.855	C6	H12B[1]	3.441
C6	H16[2]	3.559	C6	H17A[2]	2.907
C8	H3[2]	2.964	C8	H6[10]	3.521
C8	H12B[2]	3.454	C8	H14A[8]	3.401
C8	H16[2]	3.035	C8	H20C[9]	3.565
C9	H5[3]	3.520	C12	H5[6]	3.455
C12	H6[6]	3.304	C14	H4[5]	3.458

C14	H5[3]	3.040	C14	H20A[7]	3.176
C17	H19A[9]	3.401	C17	H20C[9]	3.106
C18	H4A[12]	3.302	C18	H19A[9]	3.084
C18	H20B[12]	3.412	C19	H17B[7]	3.233
C19	H18A[7]	3.212	C19	H18B[7]	3.524

Table 13. Intermolecular contacts less than 3.60 Å involving hydrogens (continued)

atom	atom	distance	atom	atom	distance
C19	H20A^{6}	3.439	C19	H20B^{6}	3.370
C20	H14C^{9}	3.311	C20	H17B^{7}	3.362
C20	H18C^{11}	3.592	C20	H19B^{1}	2.922
H4	O3^{5}	2.172	H4	C2^{5}	3.095
H4	C4^{6}	3.491	H4	C5^{6}	3.555
H4	C14^{5}	3.458	H4	H4A^{6}	3.188
H4	H5^{6}	2.834	H4	H14A^{5}	2.662
H4	H15^{7}	3.006	H4	H18B^{7}	2.782
H4	H19C^{7}	3.593	H3	O6^{2}	3.136
H3	O7^{2}	2.618	H3	C8^{2}	2.964
H3	H5^{6}	3.236	H3	H14A^{5}	3.035
H4A	O3^{3}	3.461	H4A	O4^{1}	2.955
H4A	C18^{11}	3.302	H4A	H4^{1}	3.188
H4A	H18B^{11}	2.651	H4A	H18C^{11}	3.103
H4A	H19B^{11}	3.531	H4A	H19C^{11}	3.348
H5	O4^{1}	2.429	H5	O7^{10}	2.808
H5	C3^{1}	3.244	H5	C9^{3}	3.520
H5	C12^{1}	3.455	H5	C14^{3}	3.040
H5	H4^{1}	2.834	H5	H3^{1}	3.236
H5	H12A^{1}	2.908	H5	H14A^{3}	2.299
H5	H14C^{3}	3.137	H6	O7^{10}	2.389
H6	C8^{10}	3.521	H6	C12^{1}	3.304
H6	H6^{10}	3.320	H6	H12A^{1}	2.773
H6	H12B^{1}	2.983	H6	H12B^{2}	3.429
H6	H16^{2}	3.565	H6	H17A^{2}	2.893
H10	O5^{9}	3.003	H10	H18C^{1}	2.945
H10	H20C^{9}	3.037	H12A	O1^{6}	3.065
H12A	O7^{2}	3.497	H12A	C1^{6}	3.523
H12A	C5^{6}	3.385	H12A	C6^{6}	2.855
H12A	H5^{6}	2.908	H12A	H6^{6}	2.773
H12A	H20B^{6}	3.115	H12B	O7^{2}	2.877
H12B	C6^{6}	3.441	H12B	C8^{2}	3.454

H12B	$H6^{6}$	2.983	H12B	$H6^{2}$	3.429
H14A	$O2^{3}$	3.304	H14A	$O4^{5}$	2.858
H14A	$O6^{13}$	3.134	H14A	$O7^{13}$	2.862
H14A	$C3^{5}$	3.488	H14A	$C4^{3}$	3.600
H14A	$C5^{3}$	3.059	H14A	$C8^{13}$	3.401

Table 13. Intermolecular contacts less than 3.60 Å involving hydrogens (continued)

atom	atom	distance	atom	atom	distance
H14A	H4^{5}	2.662	H14A	H3^{5}	3.035
H14A	H5^{3}	2.299	H14B	O3^{5}	2.482
H14B	O6^{13}	3.076	H14B	C2^{5}	3.160
H14B	H15^{7}	3.307	H14B	H20A^{7}	2.955
H14B	H20C^{7}	3.494	H14C	O2^{3}	3.196
H14C	O6^{13}	3.435	H14C	O7^{13}	3.382
H14C	C20^{7}	3.311	H14C	H5^{3}	3.137
H14C	H19B^{11}	3.055	H14C	H19C^{11}	3.302
H14C	H20A^{7}	2.546	H14C	H20C^{7}	3.287
H15	O3^{14}	3.033	H15	O4^{9}	3.505
H15	O5^{9}	2.685	H15	H4^{9}	3.006
H15	H14B^{9}	3.307	H16	O6^{2}	3.486
H16	O7^{2}	3.213	H16	C1^{2}	3.253
H16	C6^{2}	3.559	H16	C8^{2}	3.035
H16	H6^{2}	3.565	H17A	O1^{2}	3.217
H17A	O2^{2}	2.583	H17A	C1^{2}	3.397
H17A	C5^{2}	3.383	H17A	C6^{2}	2.907
H17A	H6^{2}	2.893	H17A	H19A^{9}	3.426
H17A	H19C^{9}	3.462	H17B	O2^{2}	3.566
H17B	O5^{9}	3.402	H17B	C19^{9}	3.233
H17B	C20^{9}	3.362	H17B	H19A^{9}	2.561
H17B	H19C^{9}	3.047	H17B	H20C^{9}	2.485
H18A	C19^{9}	3.212	H18A	H19A^{9}	2.532
H18A	H19B^{9}	3.162	H18A	H19C^{9}	3.514
H18A	H20B^{12}	3.464	H18B	O3^{14}	3.550
H18B	O4^{9}	2.704	H18B	C4^{12}	3.528
H18B	C19^{9}	3.524	H18B	H4^{9}	2.782
H18B	H4A^{12}	2.651	H18B	H19A^{9}	2.774
H18B	H19B^{9}	3.426	H18B	H20B^{12}	3.182
H18C	C20^{12}	3.592	H18C	H4A^{12}	3.103
H18C	H10^{6}	2.945	H18C	H20A^{6}	3.446
H18C	H20B^{12}	3.041	H18C	H20C^{12}	3.263

H19A	C17^{7}	3.401	H19A	C18^{7}	3.084
H19A	H17A^{7}	3.426	H19A	H17B^{7}	2.561
H19A	H18A^{7}	2.532	H19A	H18B^{7}	2.774
H19A	H20B^{6}	3.477	H19B	C20^{6}	2.922
H19B	H4A^{12}	3.531	H19B	H14C^{12}	3.055

Table 13. Intermolecular contacts less than 3.60 Å involving hydrogens (continued)

atom	atom	distance	atom	atom	distance
H19B	H18A^{7}	3.162	H19B	H18B^{7}	3.426
H19B	H20A^{6}	2.484	H19B	H20B^{6}	2.514
H19B	H20C^{6}	3.478	H19C	O2^{14}	3.134
H19C	O3^{14}	2.600	H19C	C2^{14}	3.192
H19C	H4^{9}	3.593	H19C	H4A^{12}	3.348
H19C	H14C^{12}	3.302	H19C	H17A^{7}	3.462
H19C	H17B^{7}	3.047	H19C	H18A^{7}	3.514
H20A	C14^{9}	3.176	H20A	C19^{1}	3.439
H20A	H14B^{9}	2.955	H20A	H14C^{9}	2.546
H20A	H18C^{1}	3.446	H20A	H19B^{1}	2.484
H20B	C18^{11}	3.412	H20B	C19^{1}	3.370
H20B	H12A^{1}	3.115	H20B	H18A^{11}	3.464
H20B	H18B^{11}	3.182	H20B	H18C^{11}	3.041
H20B	H19A^{1}	3.477	H20B	H19B^{1}	2.514
H20C	O6^{7}	2.948	H20C	C8^{7}	3.565
H20C	C17^{7}	3.106	H20C	H10^{7}	3.037
H20C	H14B^{9}	3.494	H20C	H14C^{9}	3.287
H20C	H17B^{7}	2.485	H20C	H18C^{11}	3.263
H20C	H19B^{1}	3.478			

Symmetry Operators:

(1) X+1,Y,Z
(2) -X+1,-Y,-Z+1
(3) -X+2,-Y,-Z+2
(4) -X+1,Y+1/2-1,-Z+1/2+1
(5) -X+1,-Y,-Z+2
(6) X-1,Y,Z
(7) X,-Y+1,Z+1
(8) X,Y,Z-1
(9) X,-Y+1,Z
(10) -X+2,-Y,-Z+1
(11) X+1,-Y+1,Z+1
(12) X-1,-Y+1,Z
(13) X,Y,Z+1
(14) -X+1,Y+1/2,-Z+1/2+1

Datablock xcalibur - ellipsoid plot

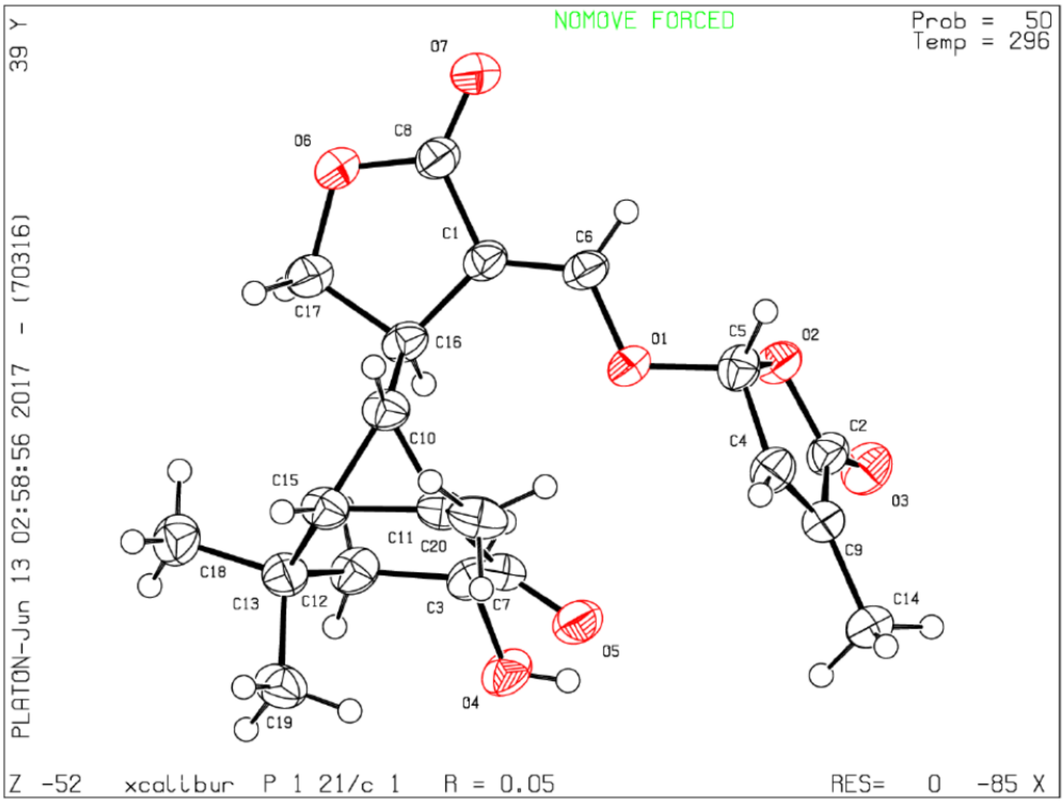

Supplementary Fig. 6. ORTEP of C2'-epi avenaol with probability ellipsoids.

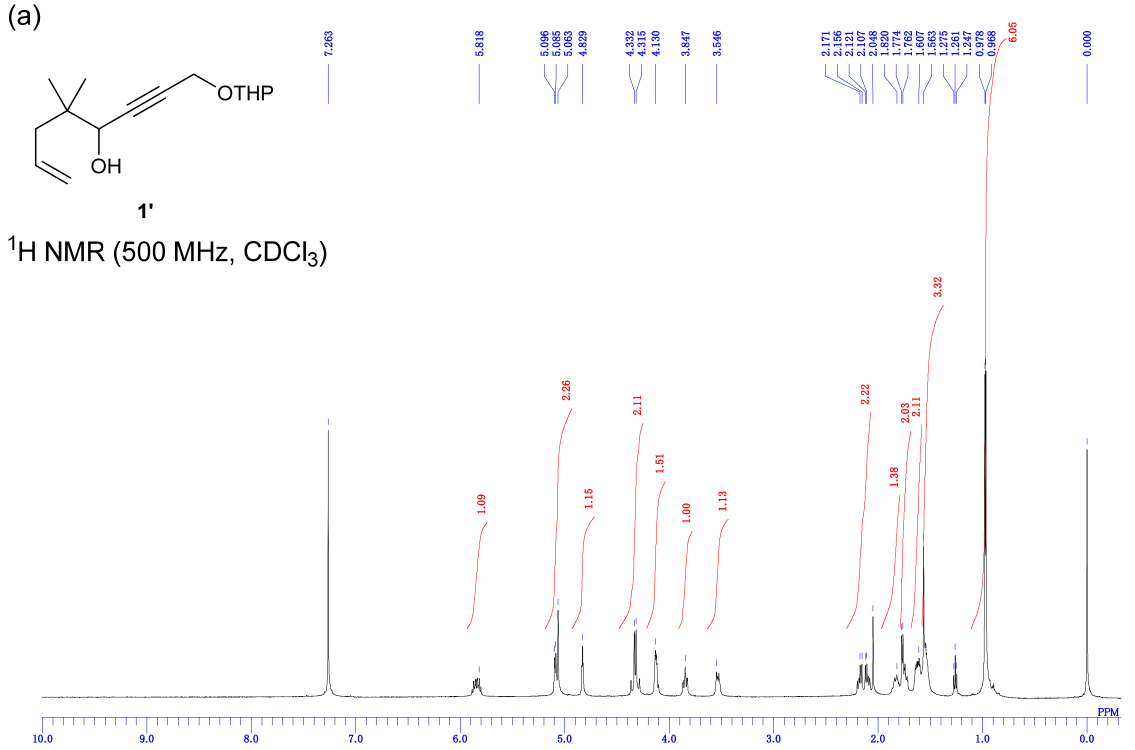

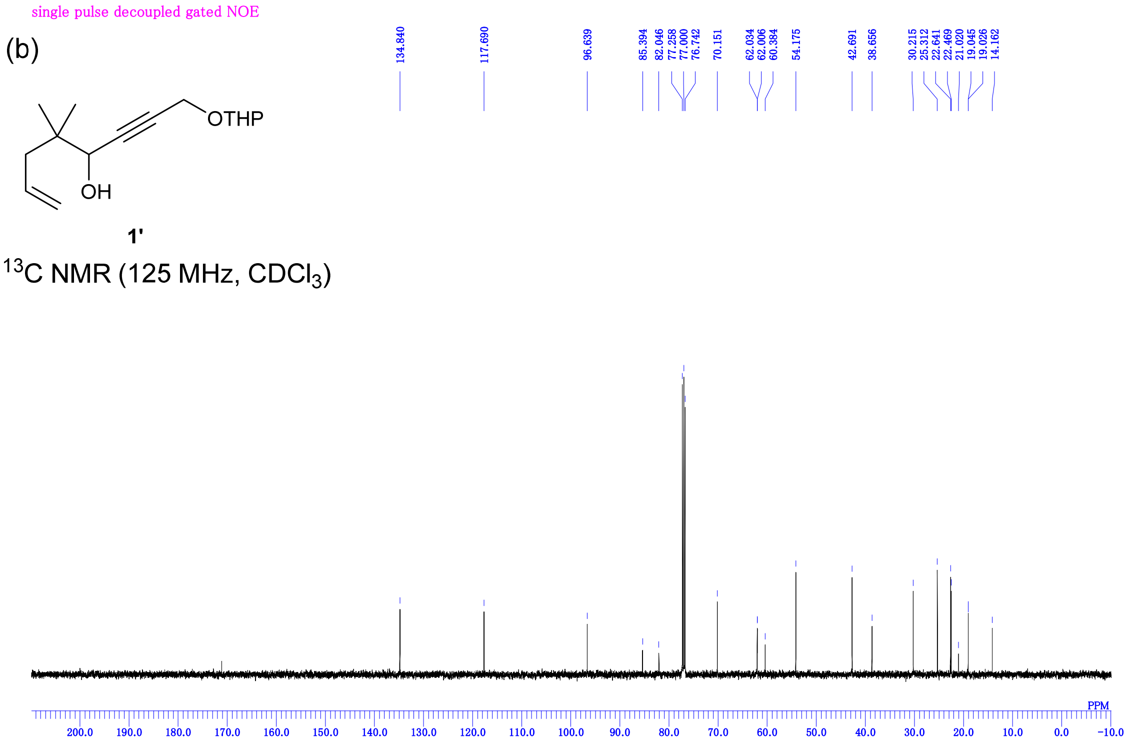

Supplementary Fig. 7. ^{1}H (a) and ^{13}C NMR (b) spectra of 1'

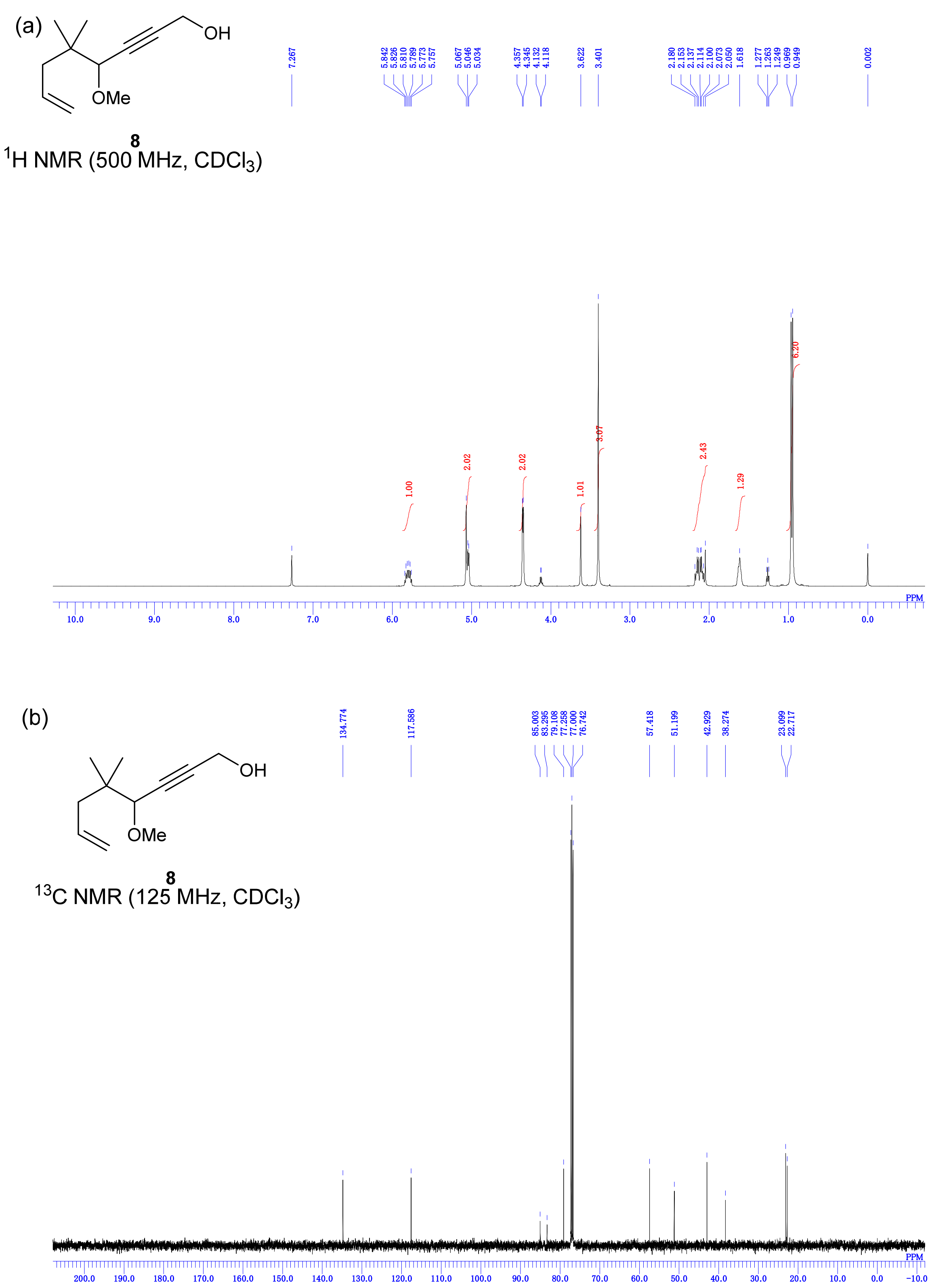

Supplementary Fig. 8. ^{1}H (a) and ^{13}C NMR (b) spectra of 8

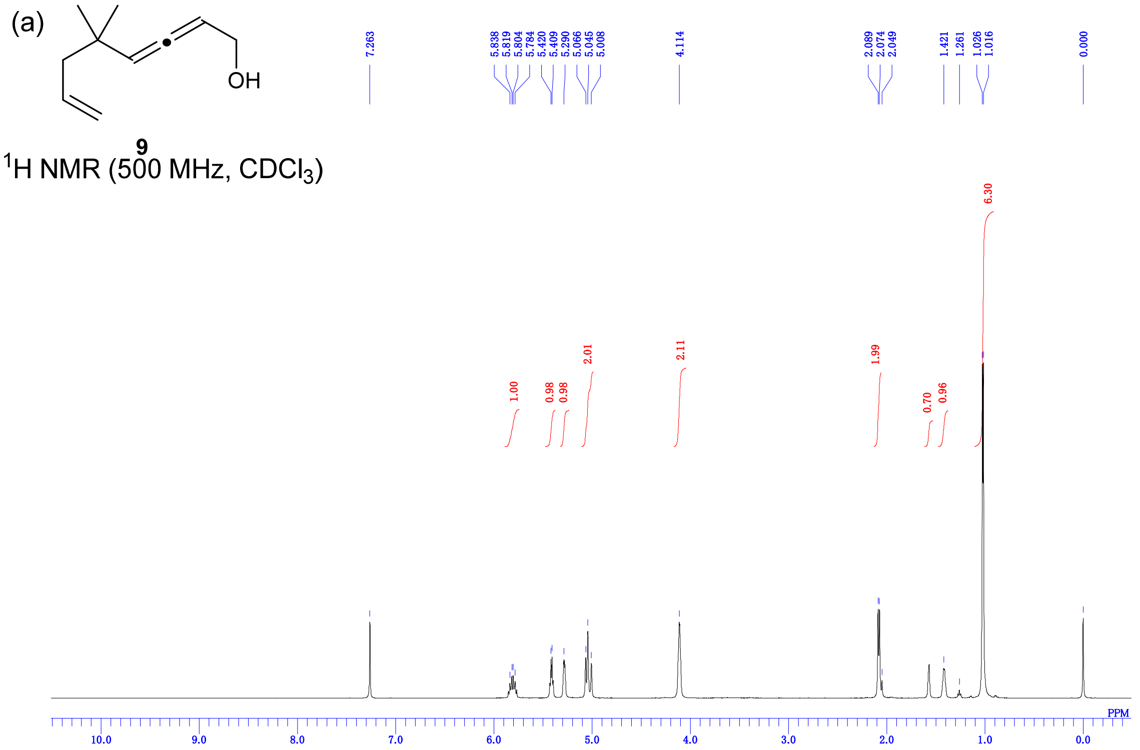

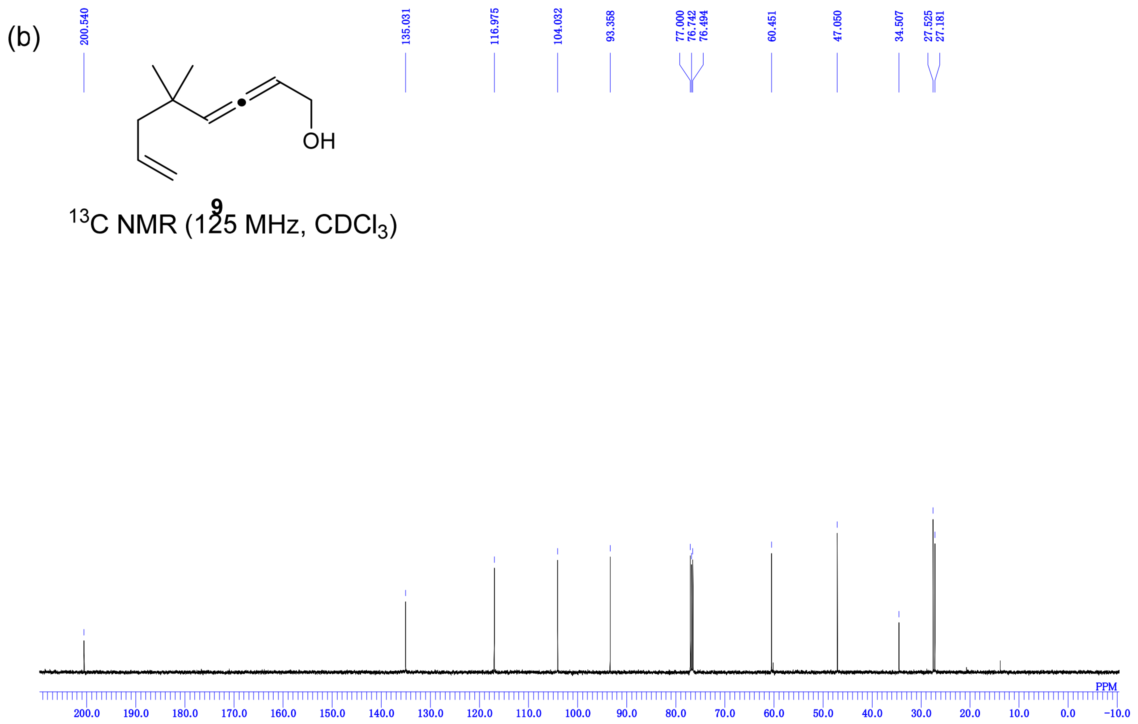

Supplementary Fig. 9. ^{1}H (a) and ^{13}C NMR (b) spectra of 9

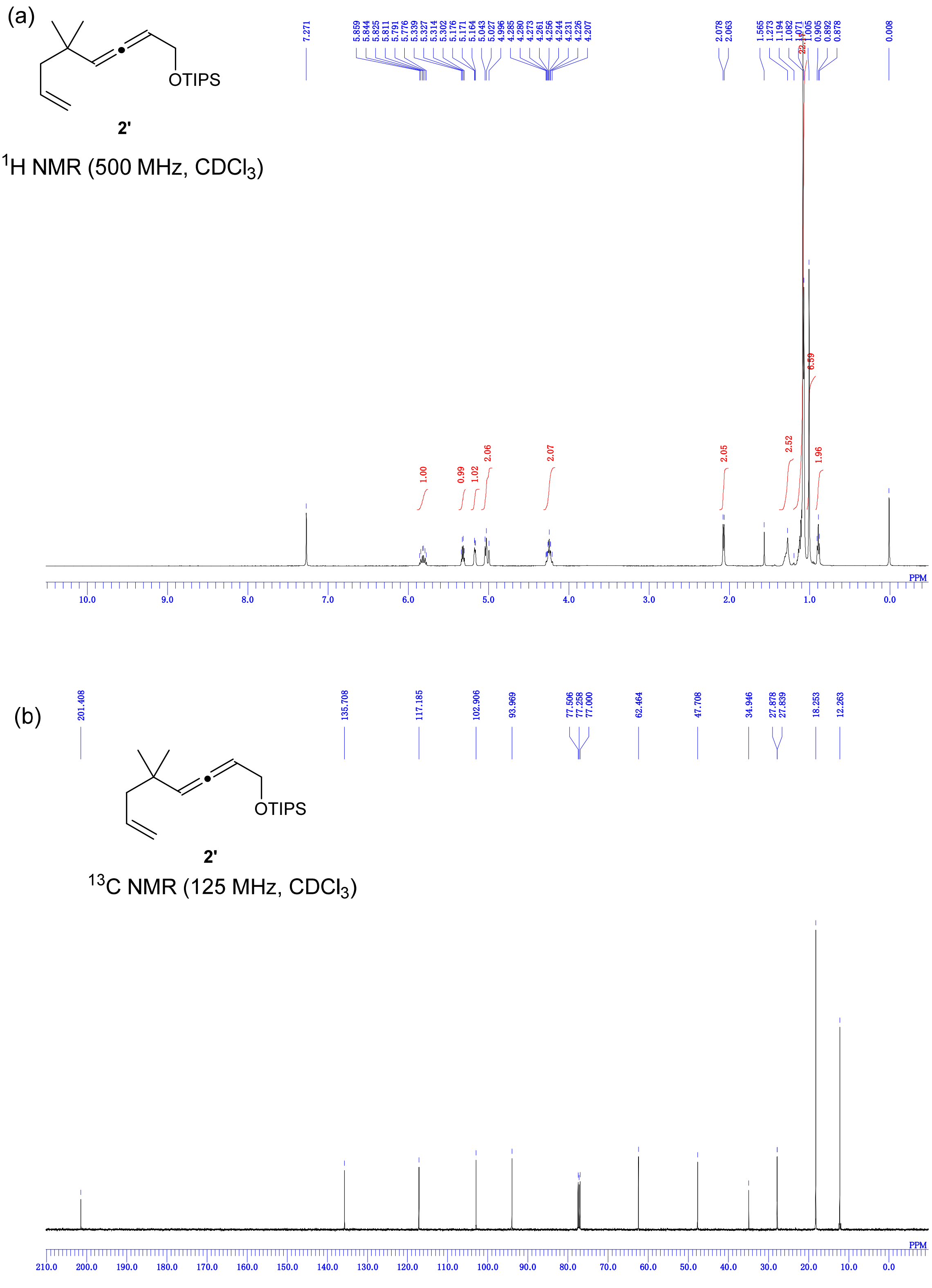

Supplementary Fig. 10. ^{1}**H (a) and** ^{13}**C NMR (b) spectra of 2'**

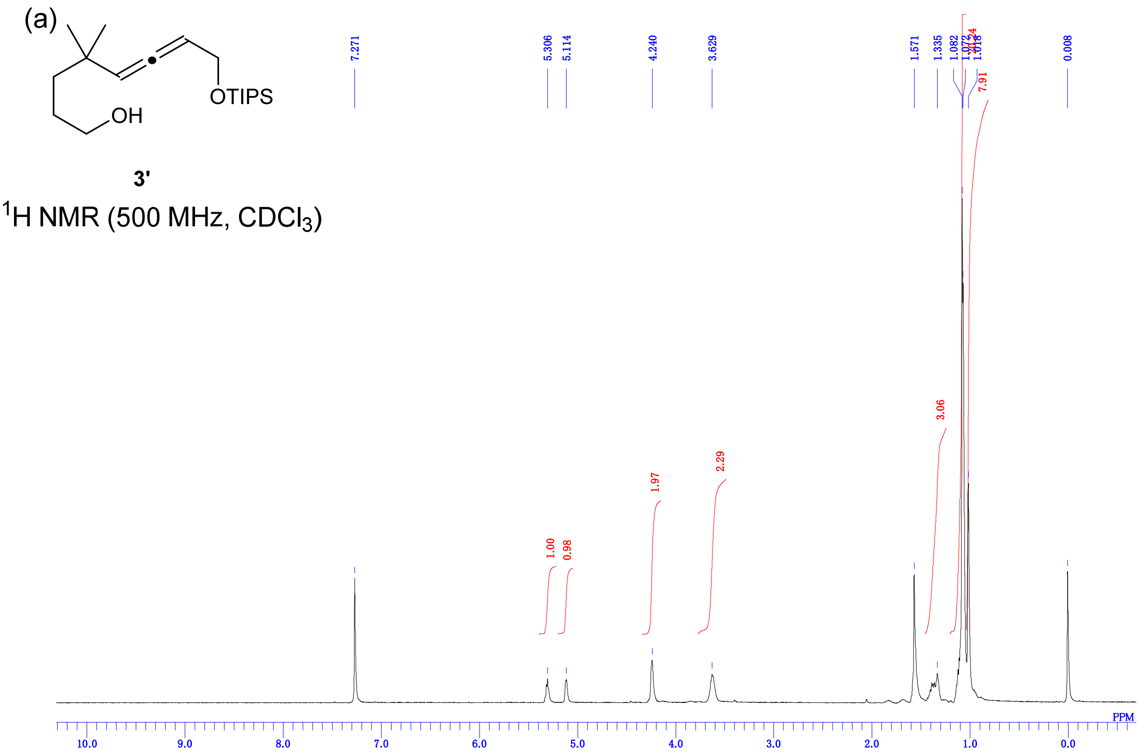

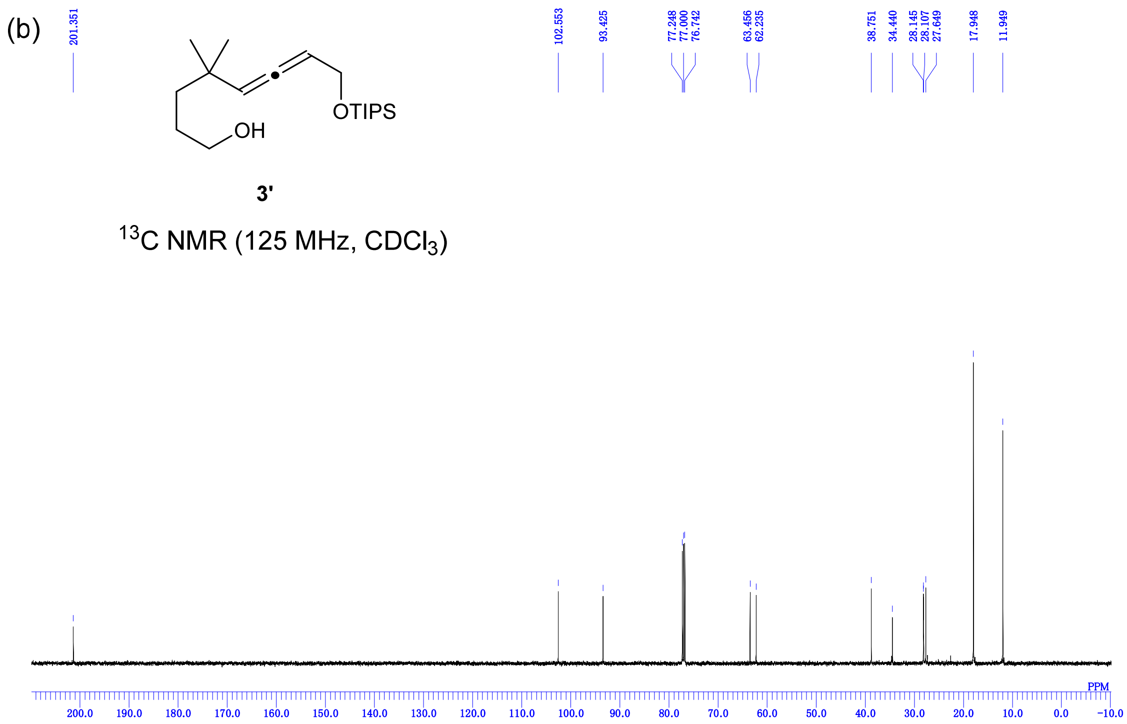

Supplementary Fig. 11. ^{1}**H (a) and** ^{13}**C NMR (b) spectra of 3'**

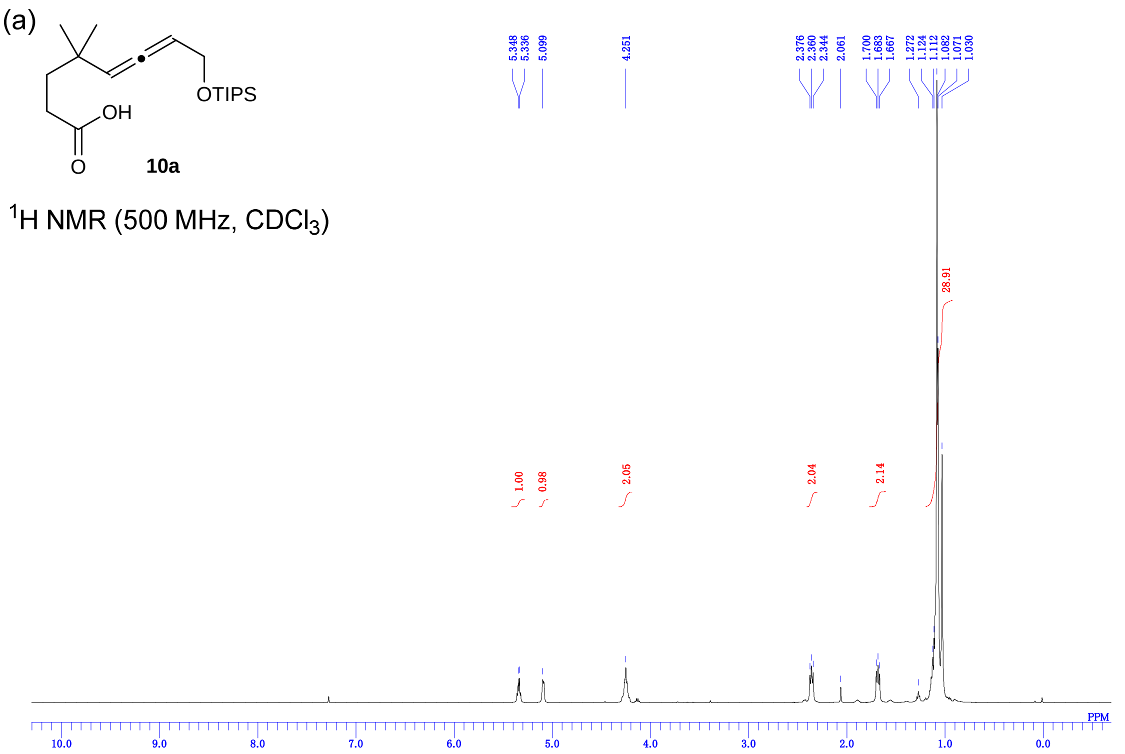

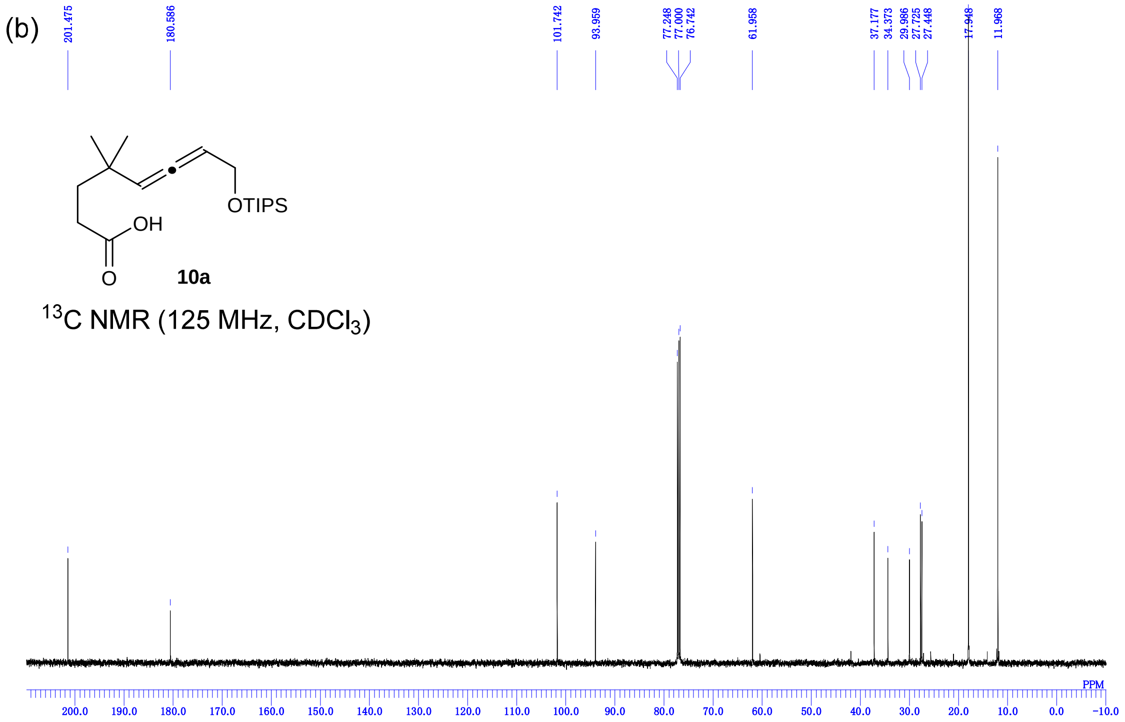

Supplementary Fig. 12. ^{1}H (a) and ^{13}C NMR (b) spectra of 10a

(a)

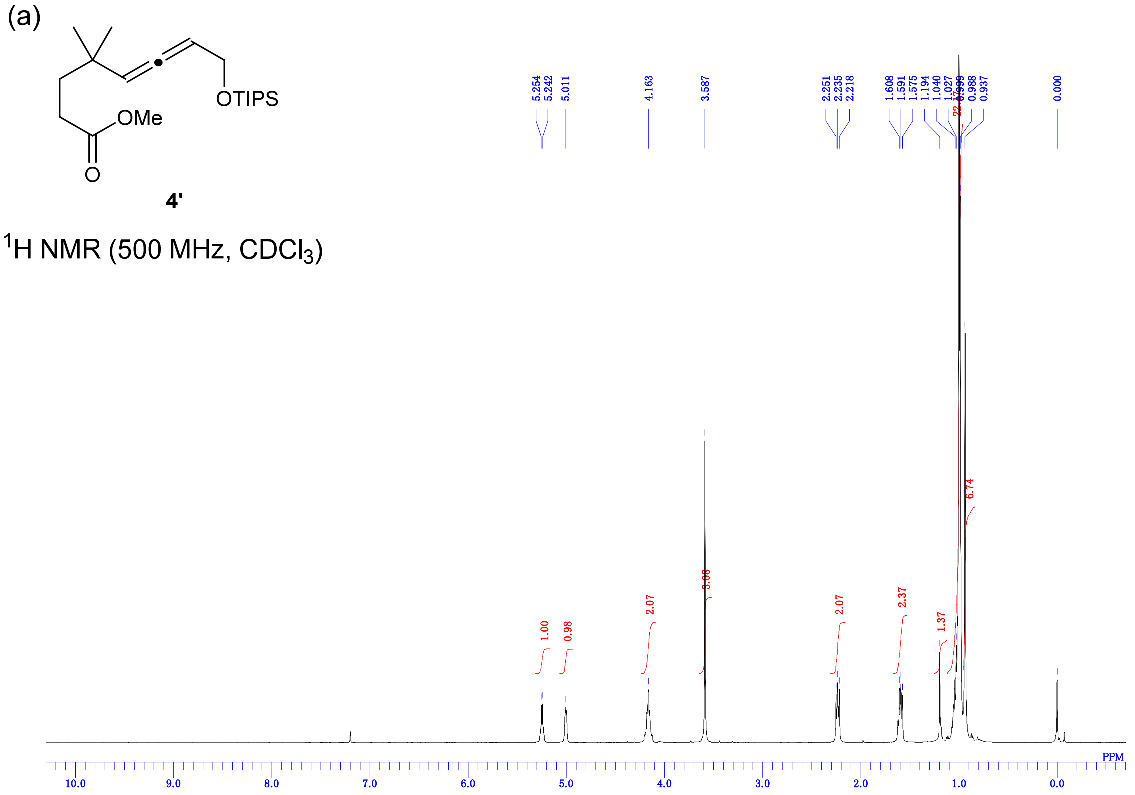

(b)

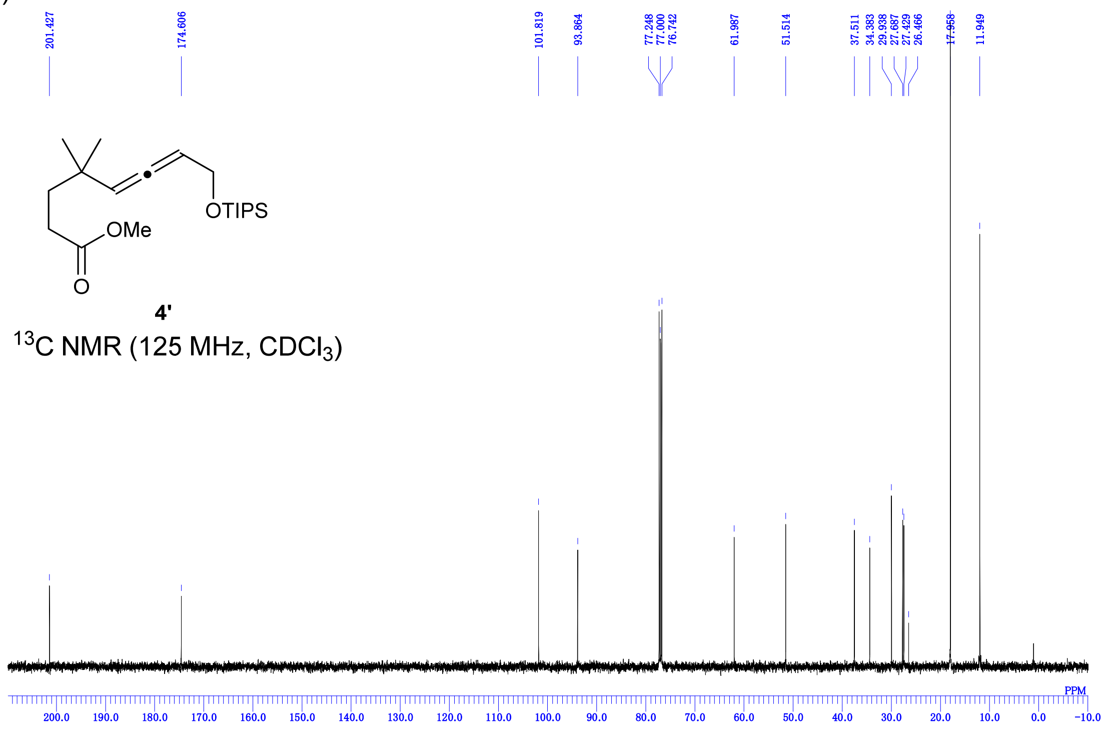

Supplementary Fig. 13. ^{1}H (a) and ^{13}C NMR (b) spectra of 4'

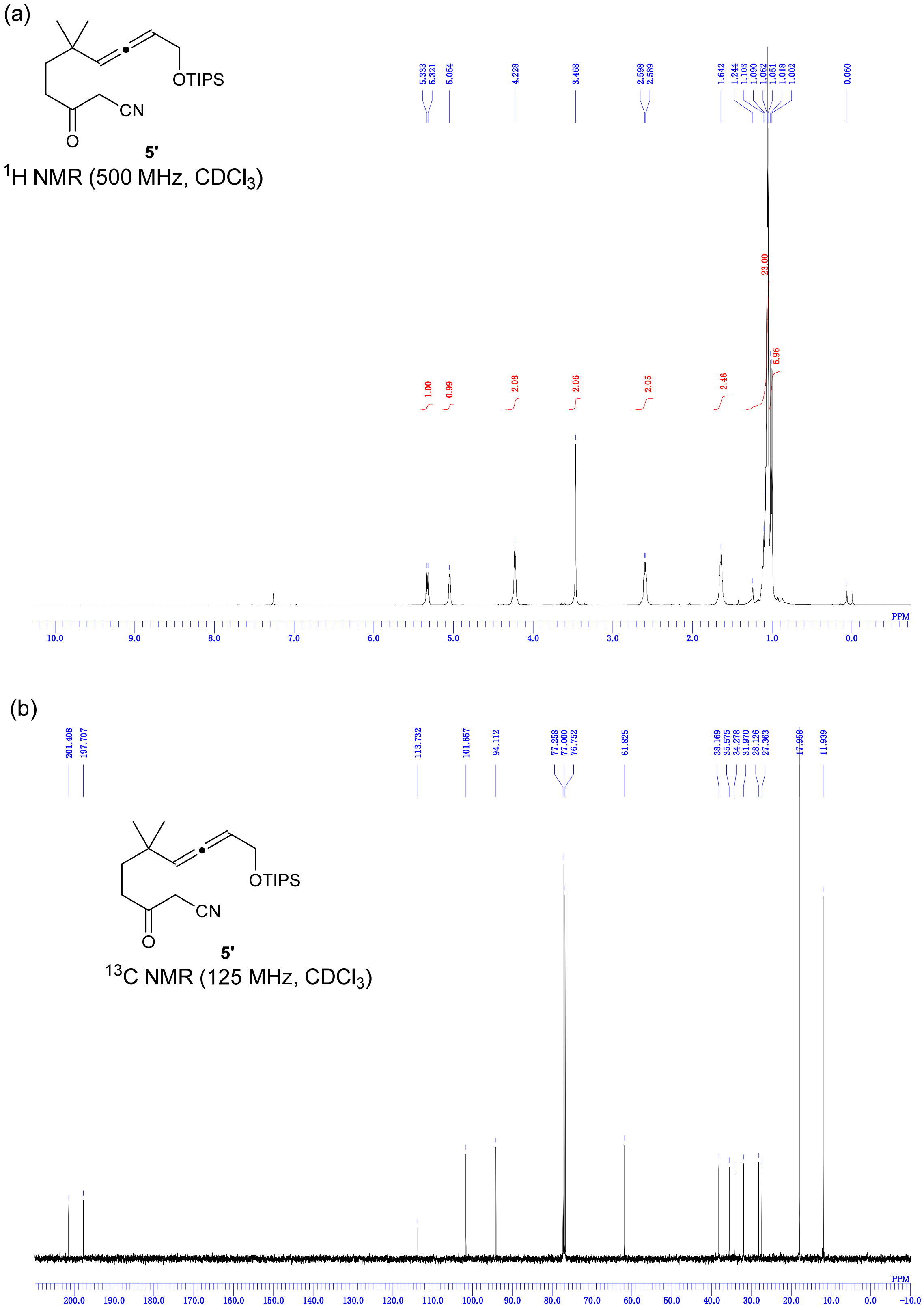

Supplementary Fig. 14. ^{1}H (a) and ^{13}C NMR (b) spectra of 5’

(a)

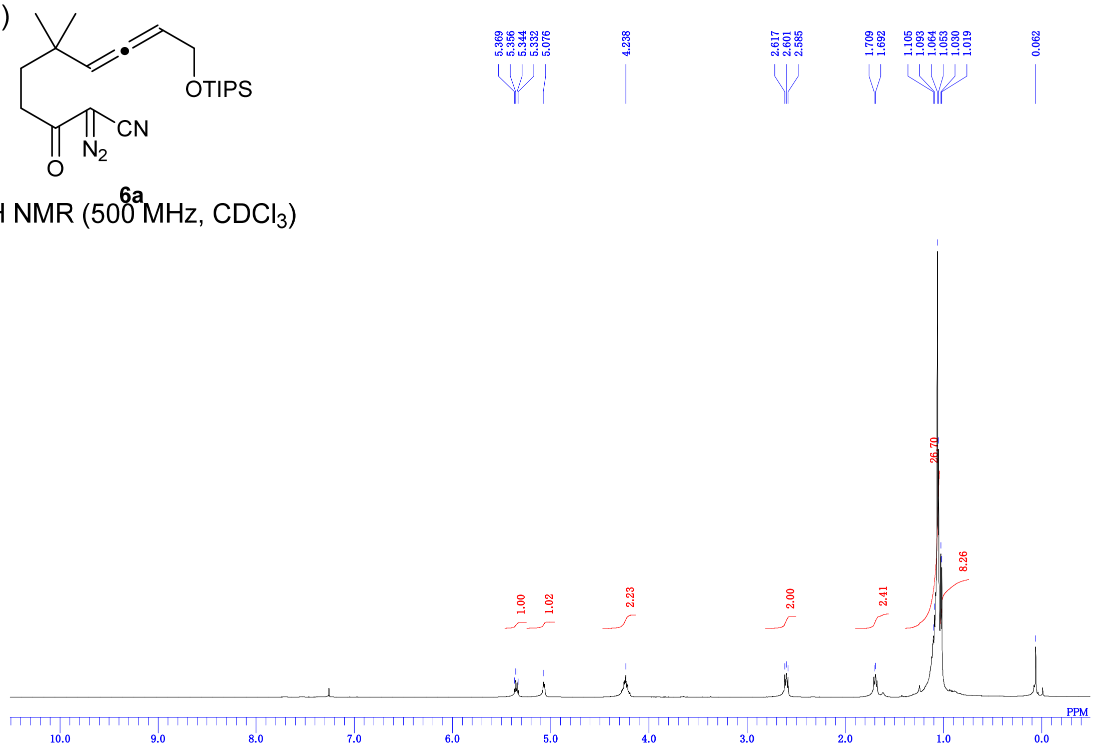

(b)

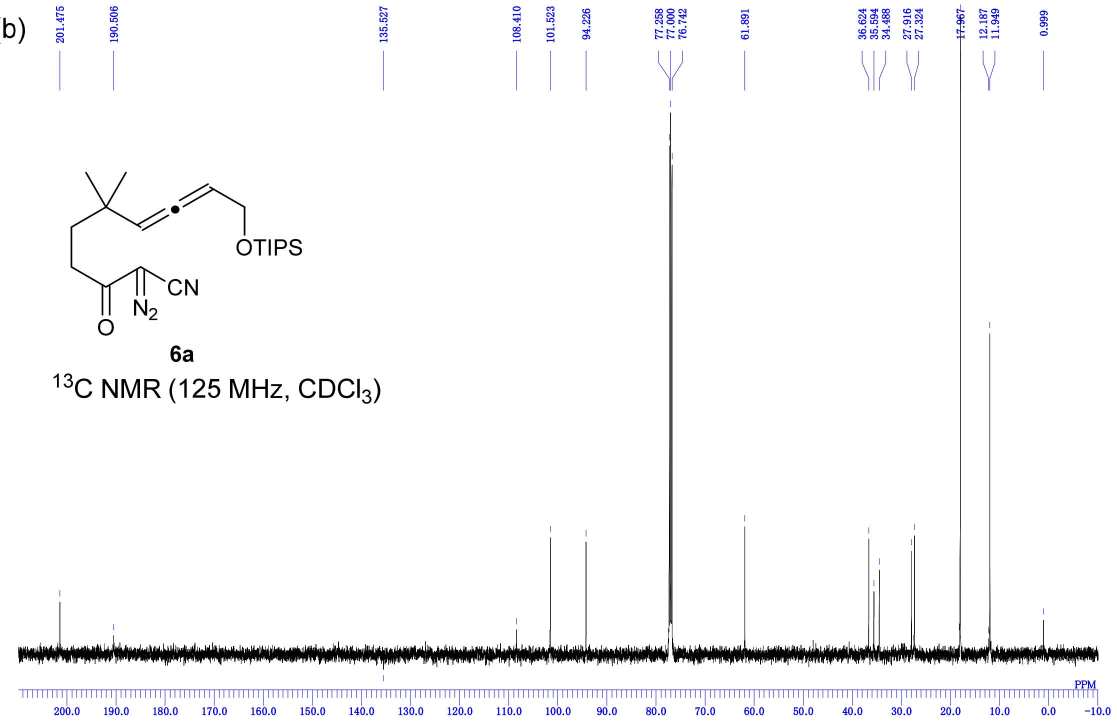

Supplementary Fig. 15. ^{1}H (a) and ^{13}C NMR (b) spectra of 6a

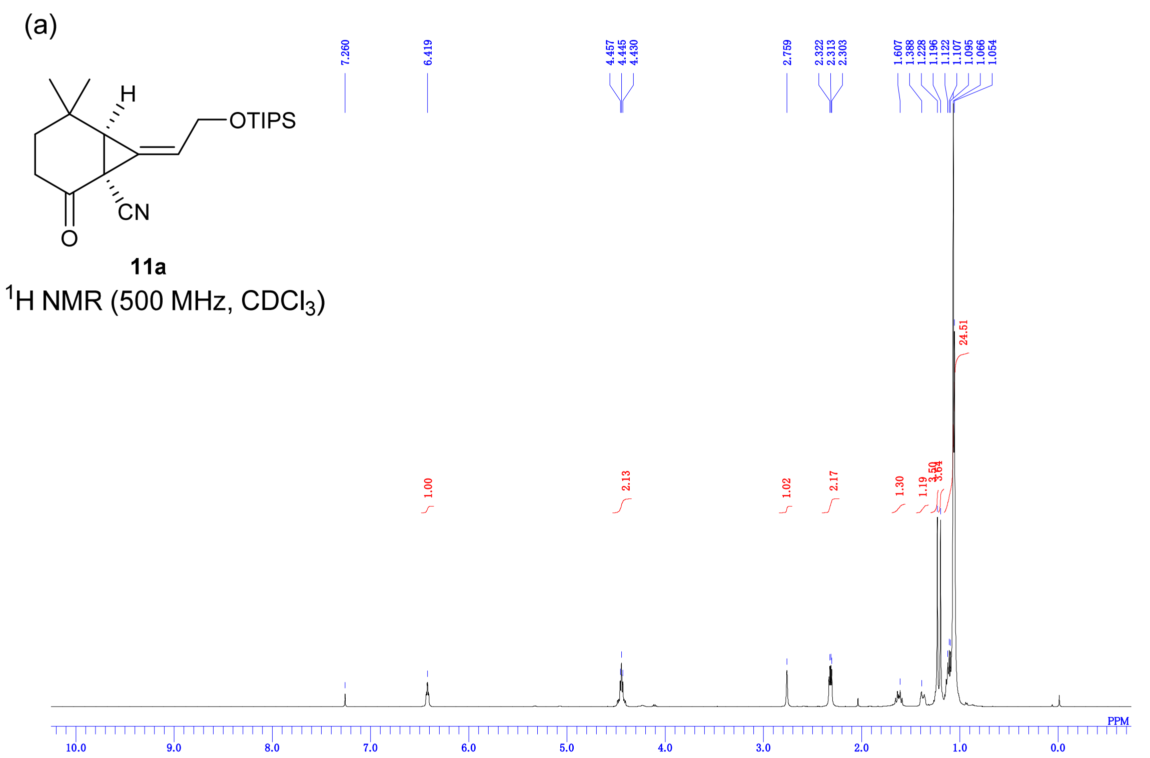

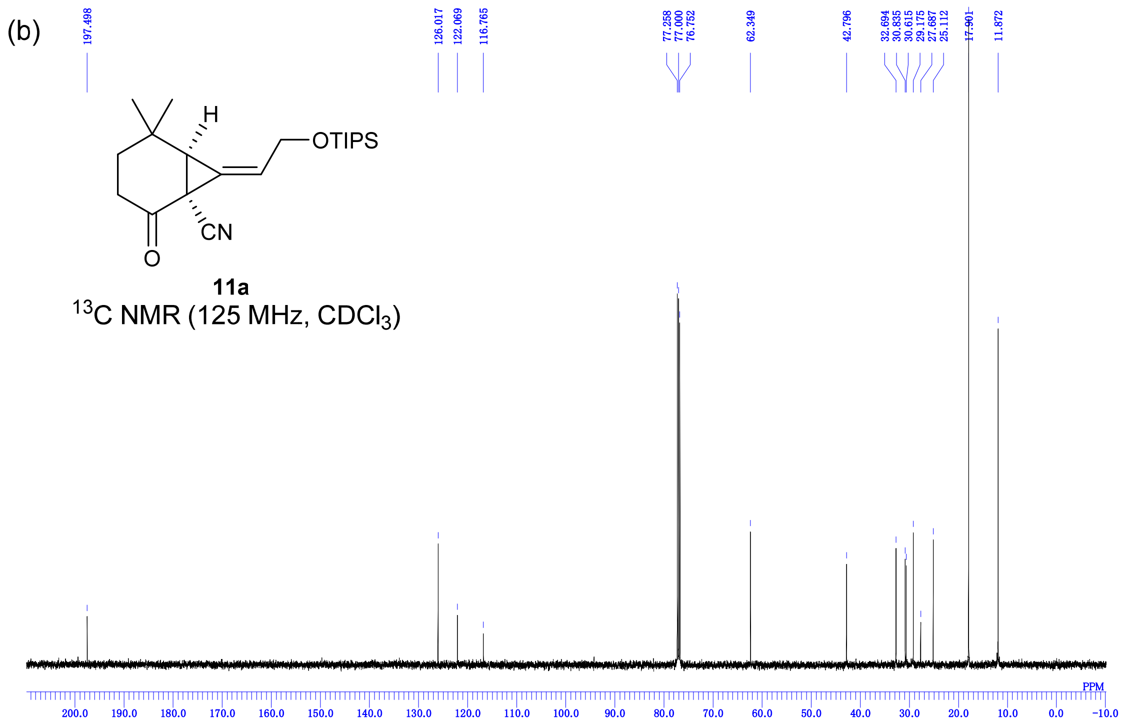

Supplementary Fig. 16. ^{1}H (a) and ^{13}C NMR (b) spectra of 11a

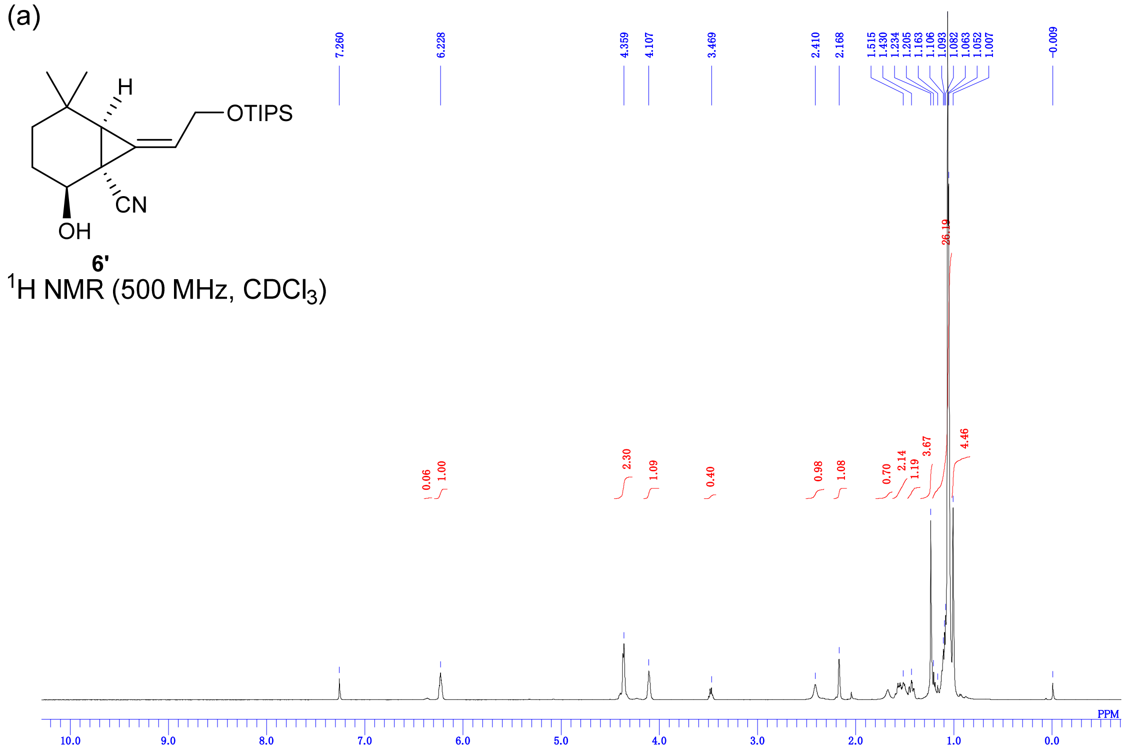

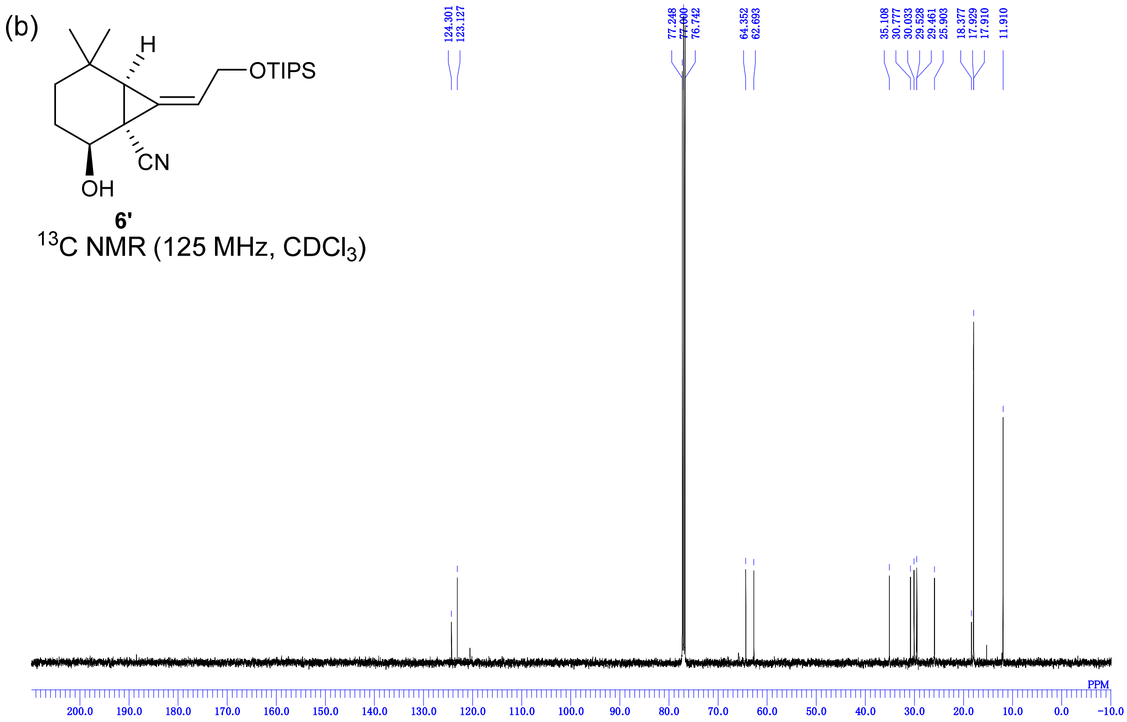

Supplementary Fig. 17. ^{1}H (a) and ^{13}C NMR (b) spectra of 6'

(a)

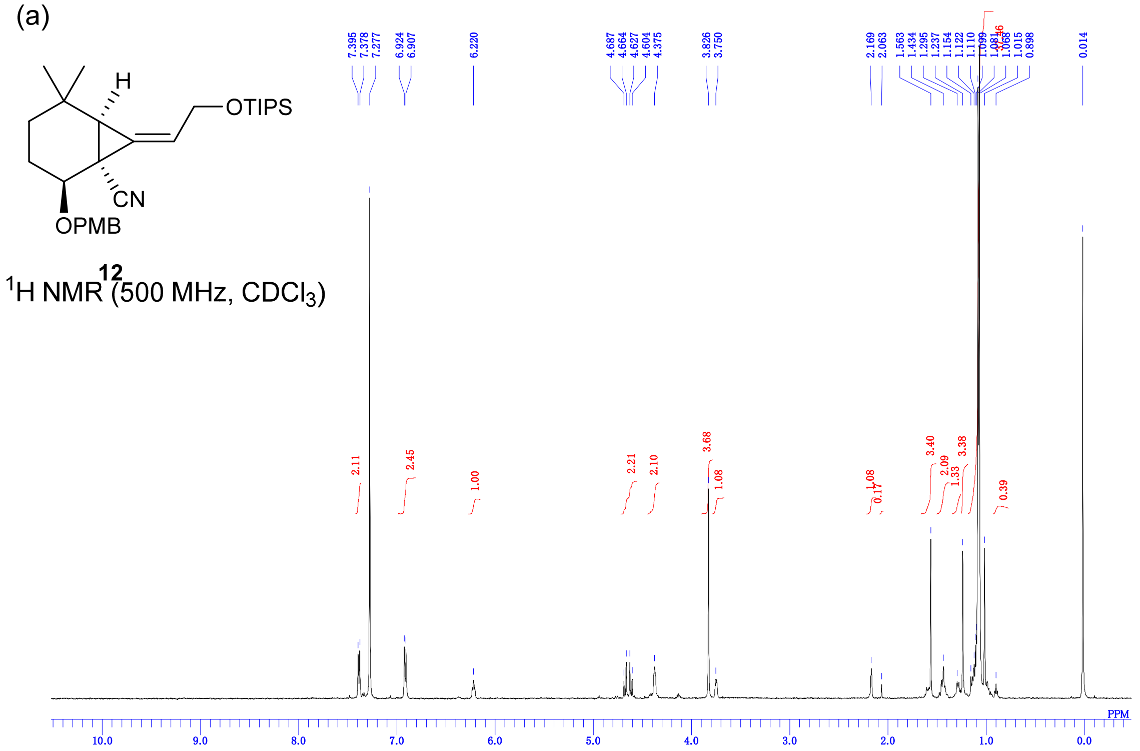

(b)

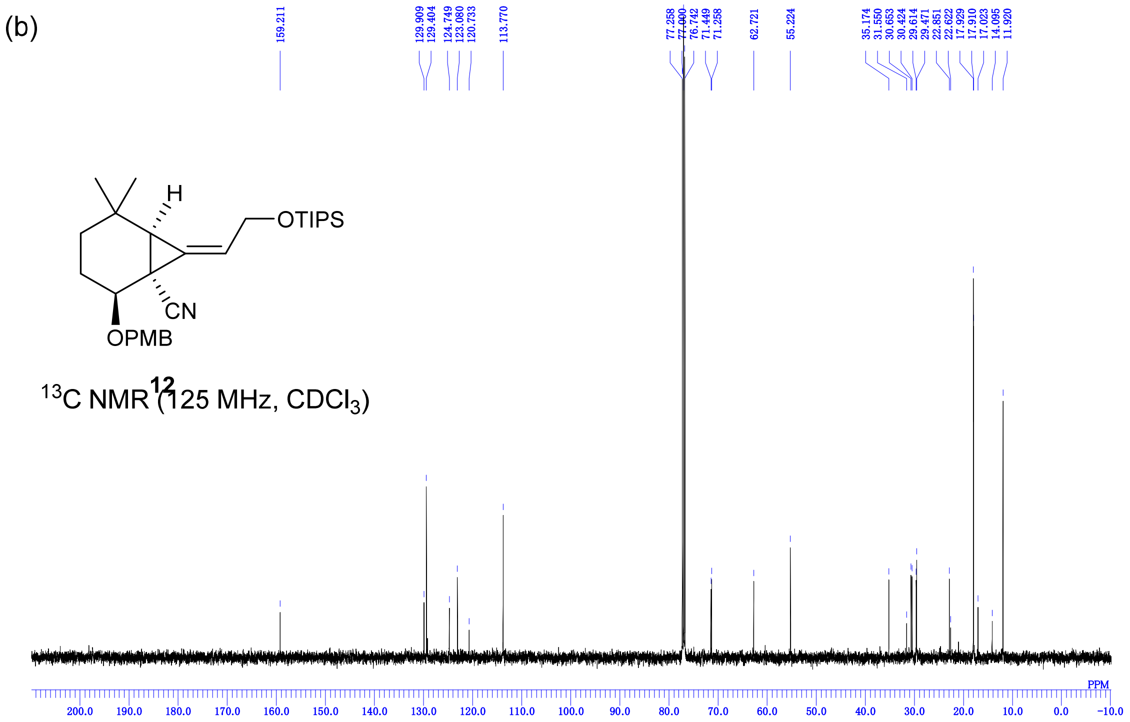

Supplementary Fig. 18. ^{1}H (a) and ^{13}C NMR (b) spectra of 12

(a)

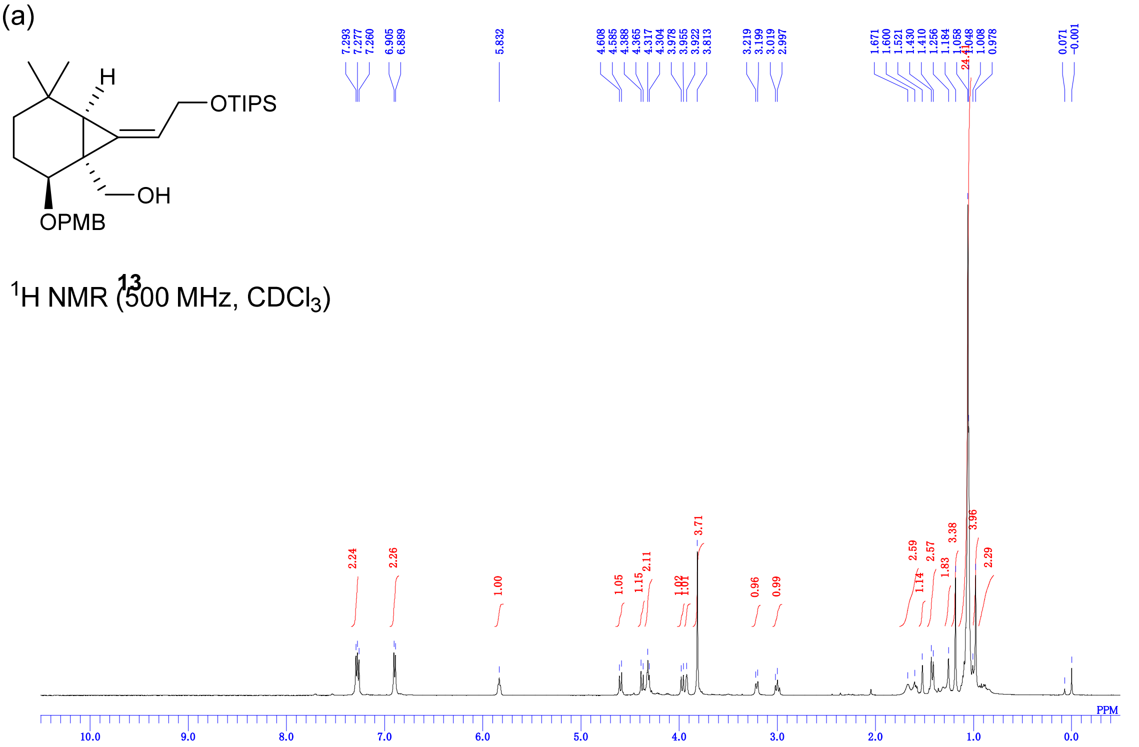

(b)

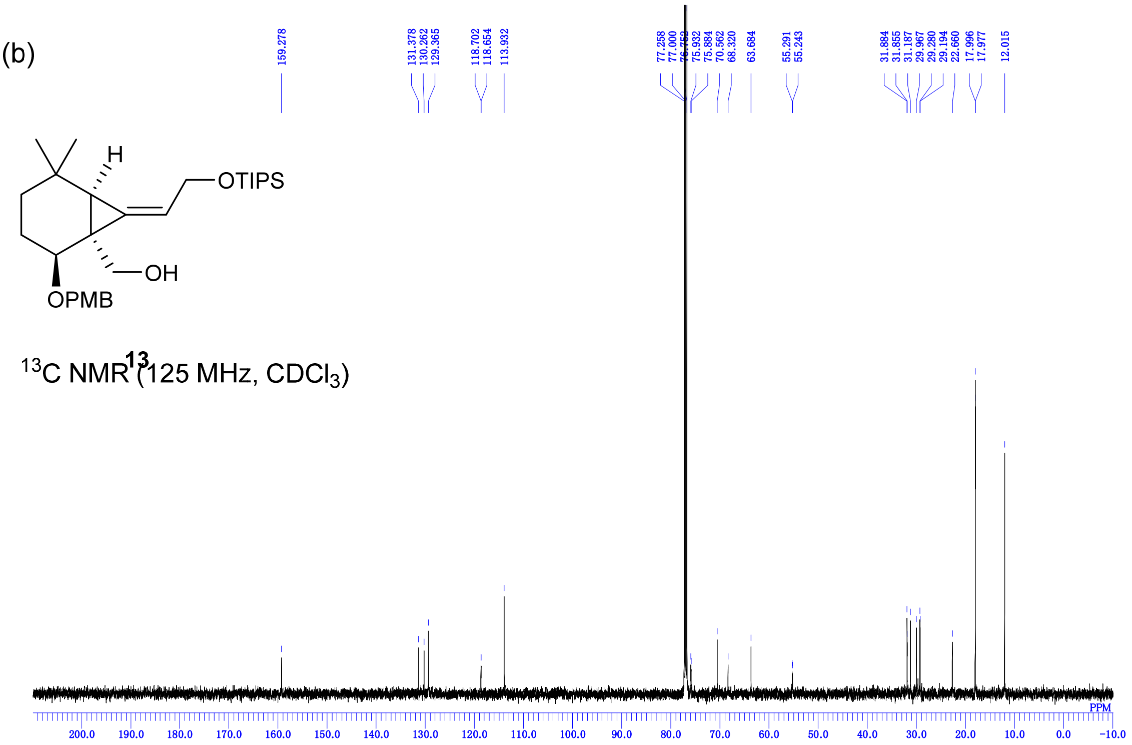

Supplementary Fig. 19. ^{1}H (a) and ^{13}C NMR (b) spectra of 13

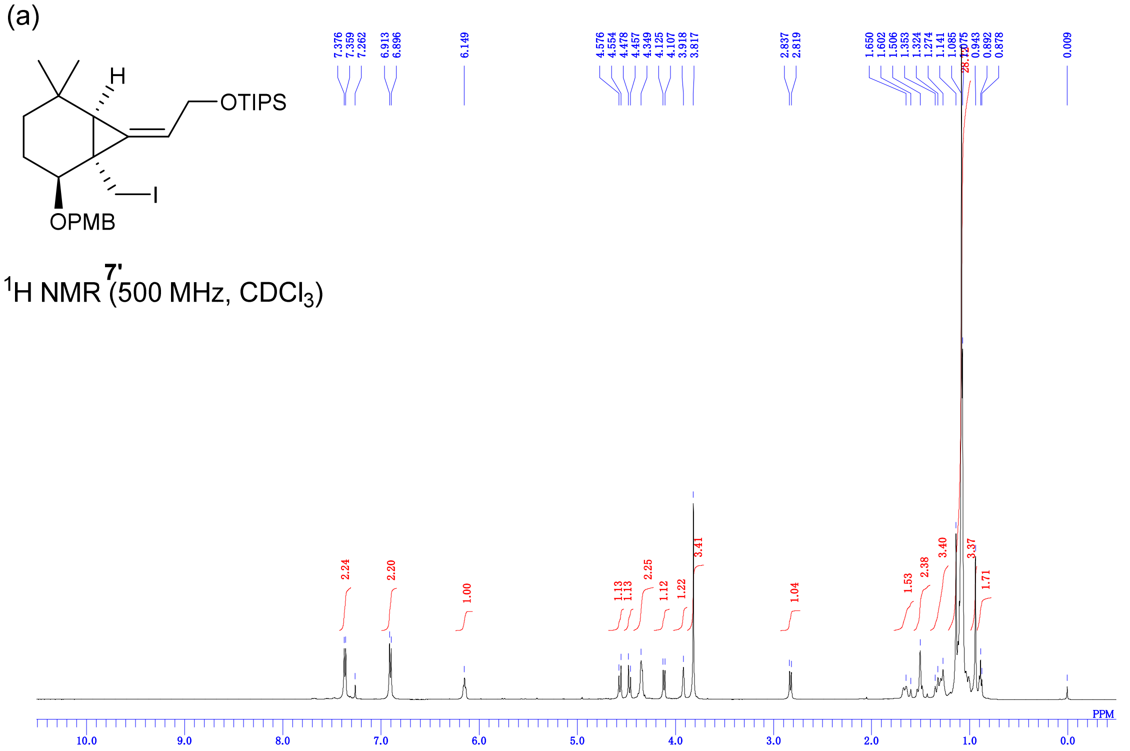

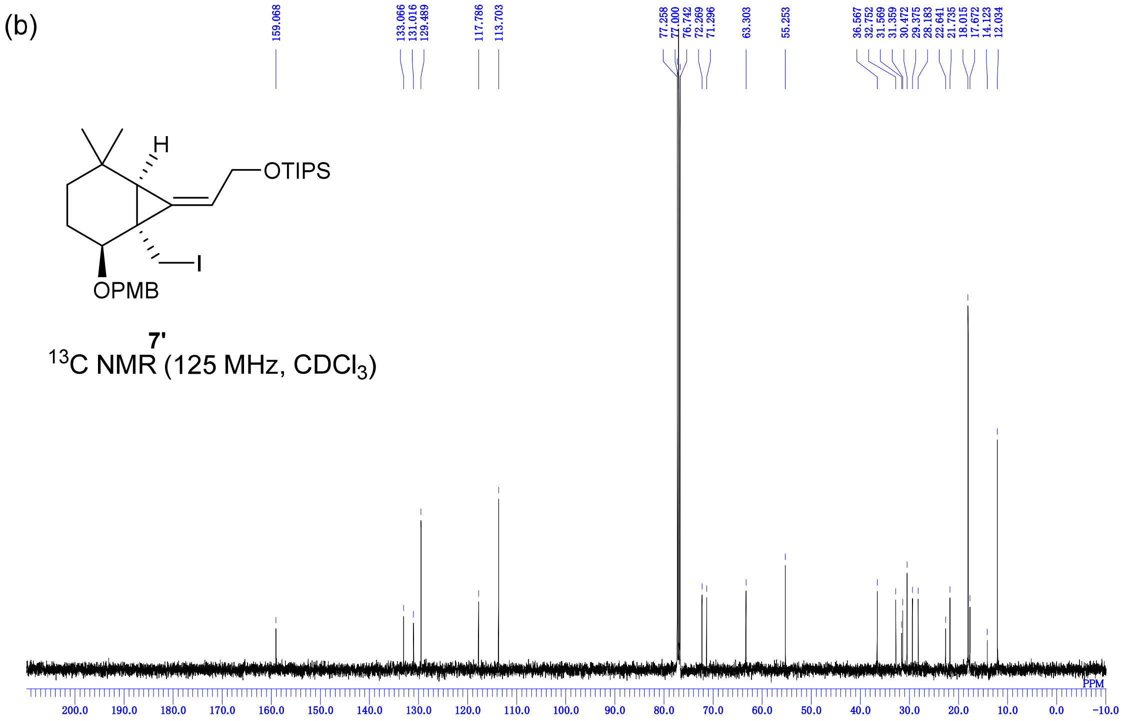

Supplementary Fig. 20. ^{1}H (a) and ^{13}C NMR (b) spectra of 7’

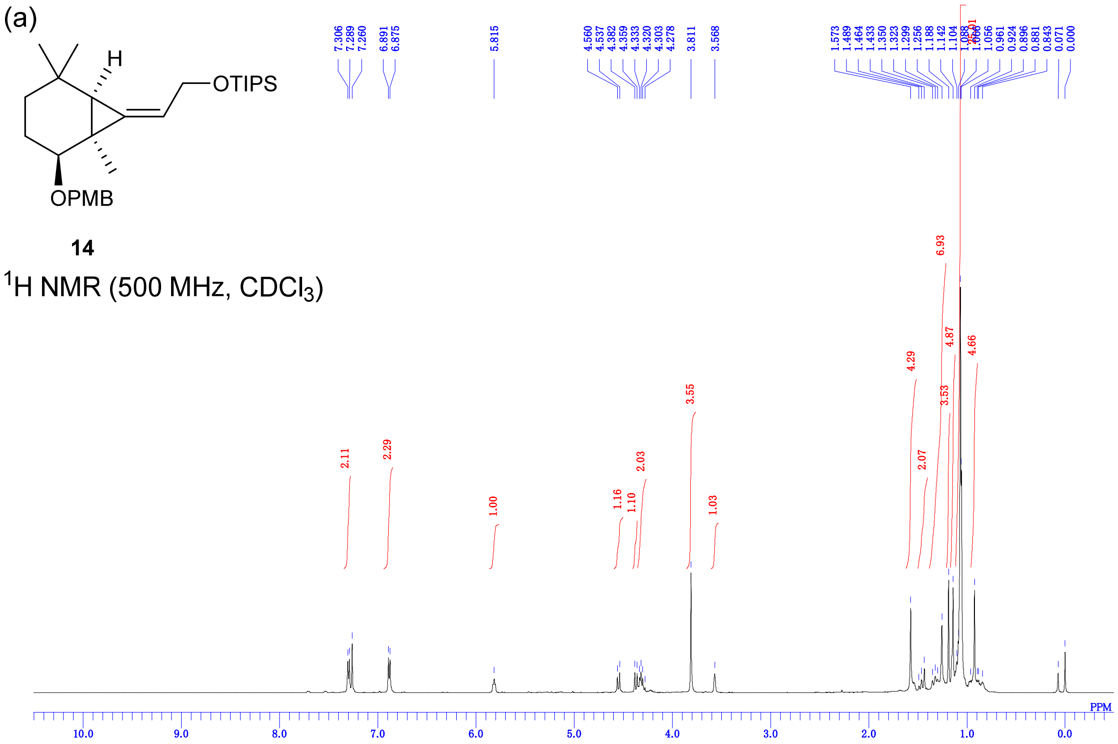

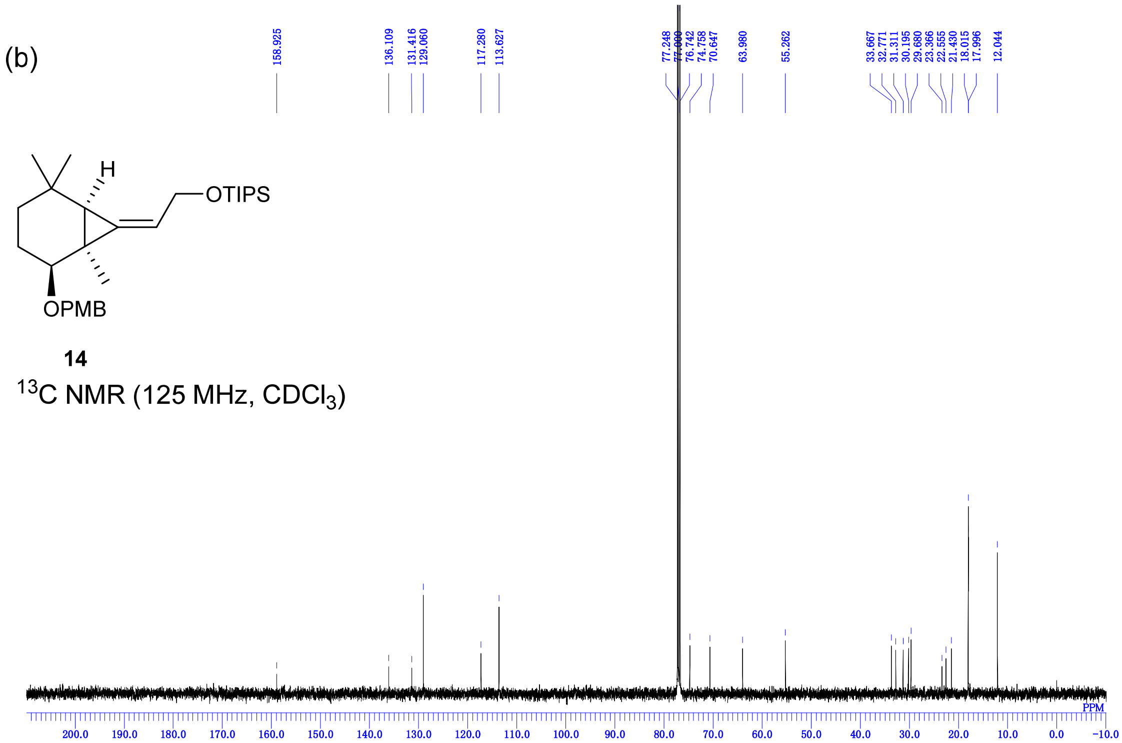

Supplementary Fig. 21. ^{1}H (a) and ^{13}C NMR (b) spectra of 14'

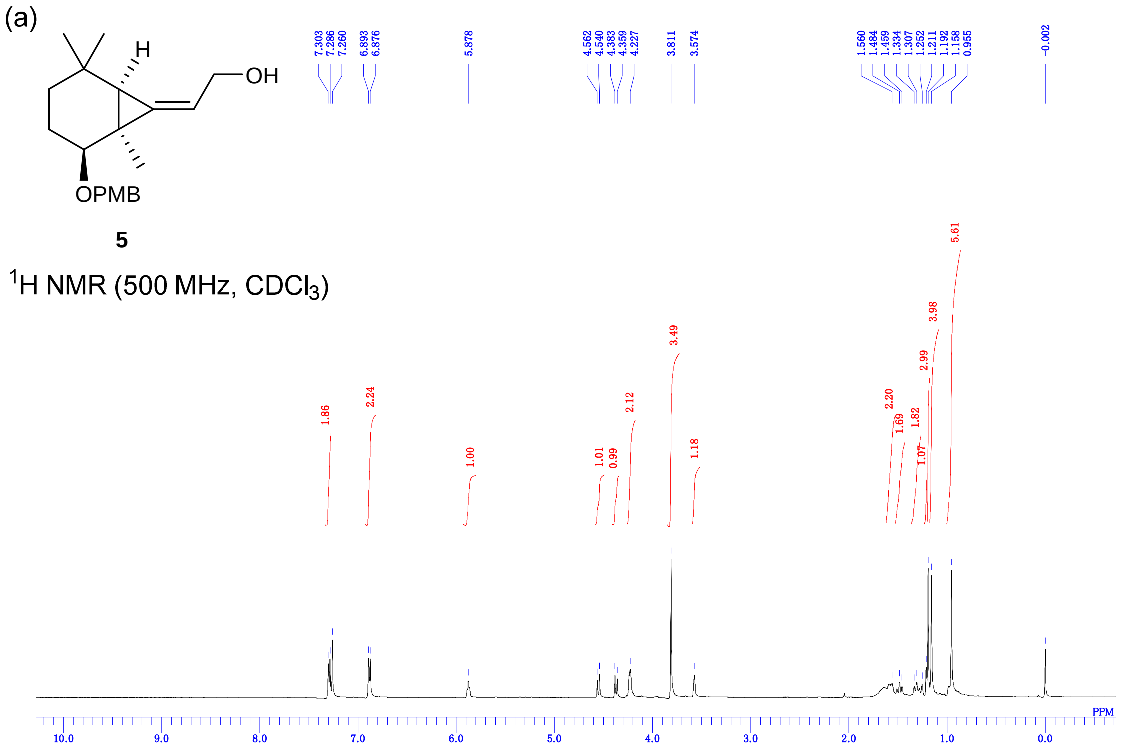

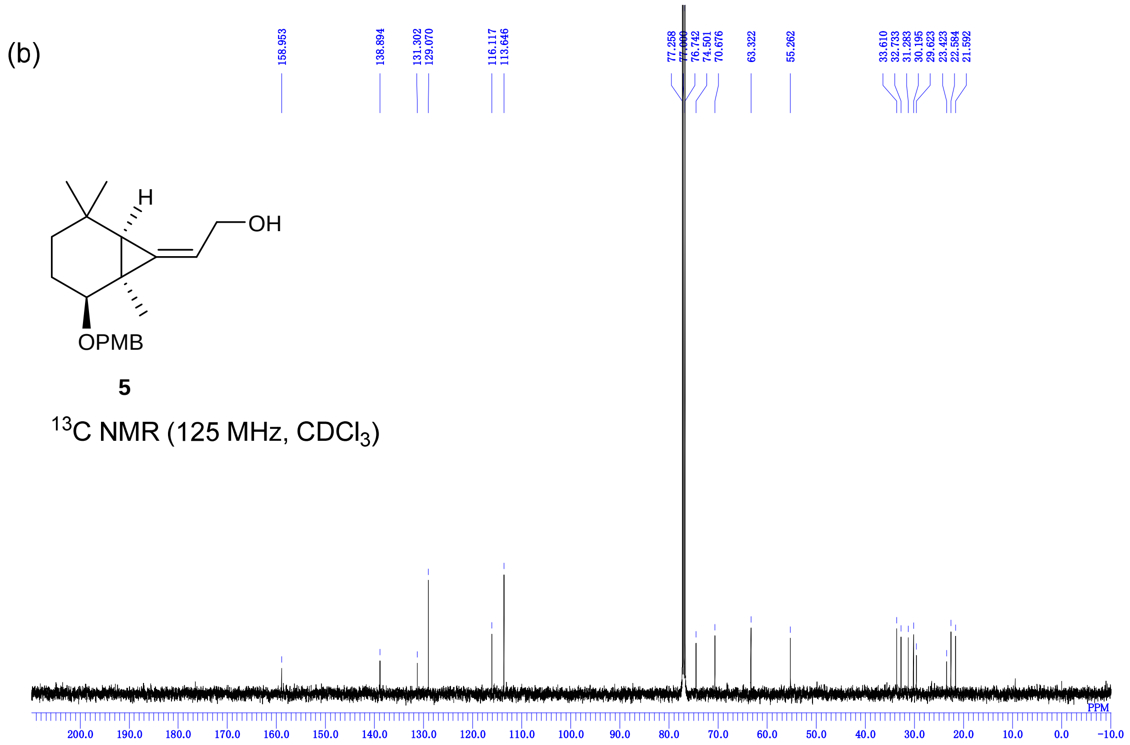

Supplementary Fig. 22. **^{1}H (a) and ^{13}C NMR (b) spectra of 5**

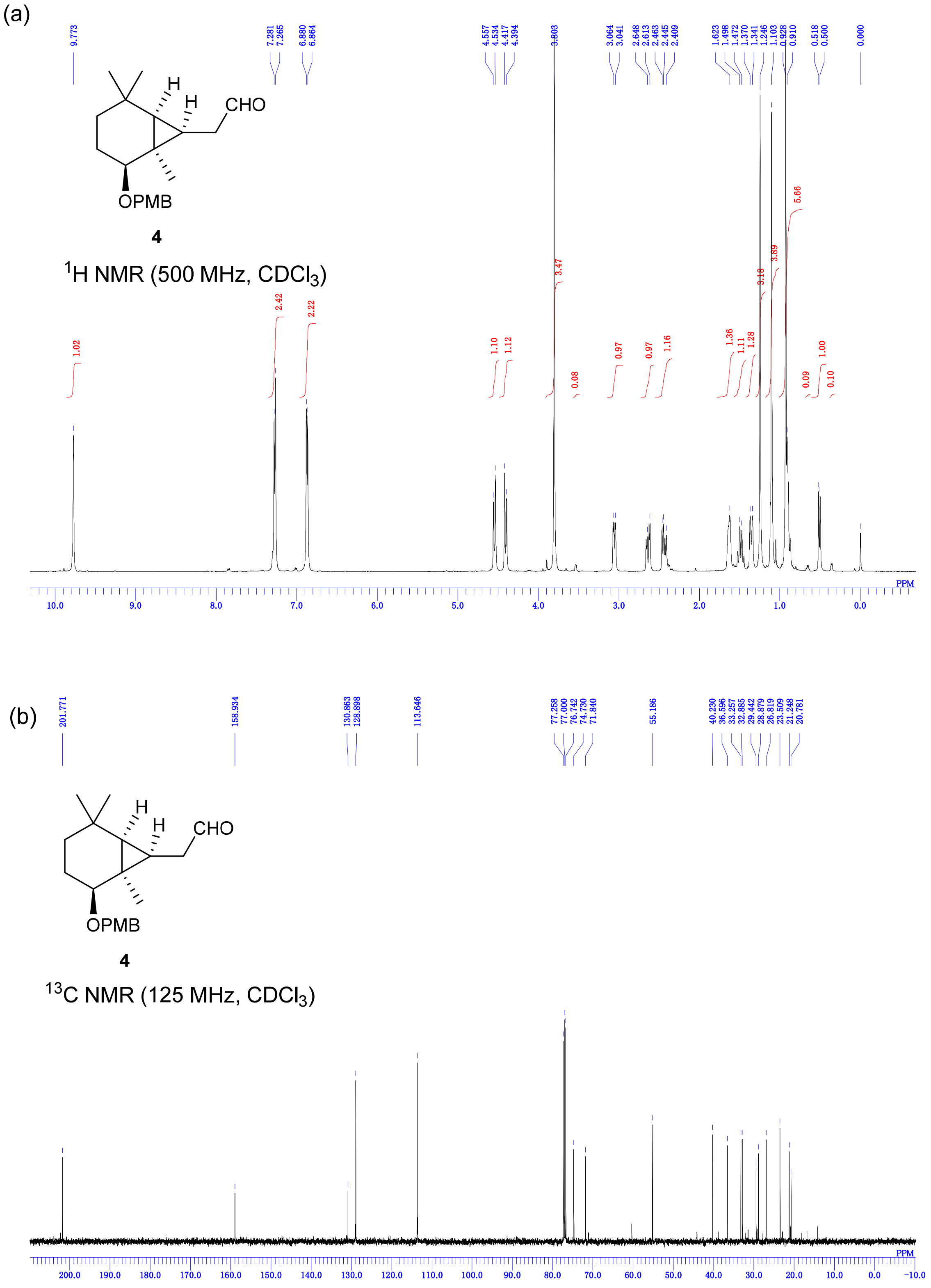

Supplementary Fig. 23. ^{1}**H (a) and** ^{13}**C NMR (b) spectra of 4**

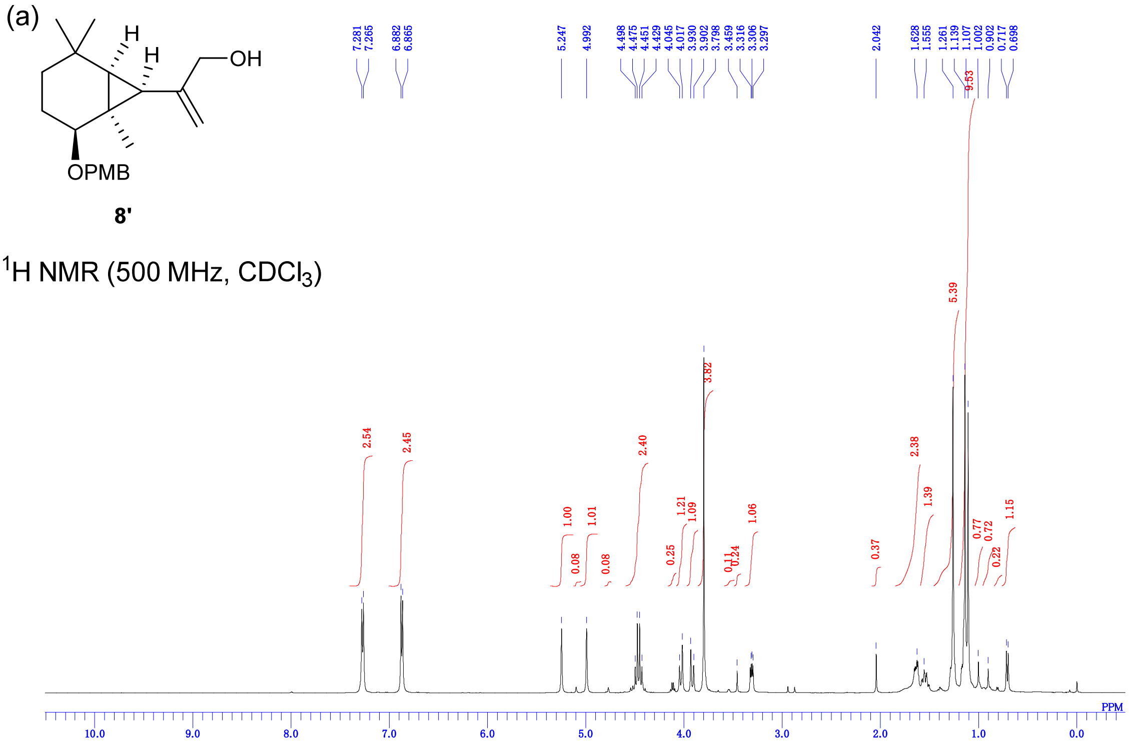

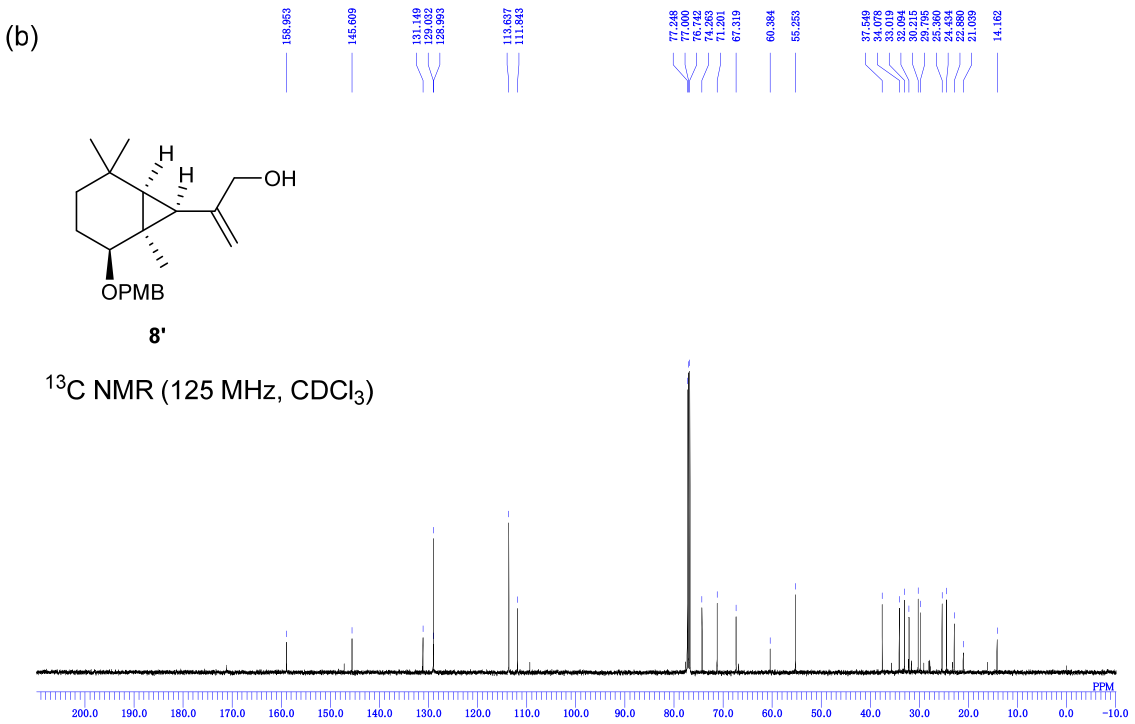

Supplementary Fig. 24. ^{1}H (a) and ^{13}C NMR (b) spectra of 8'

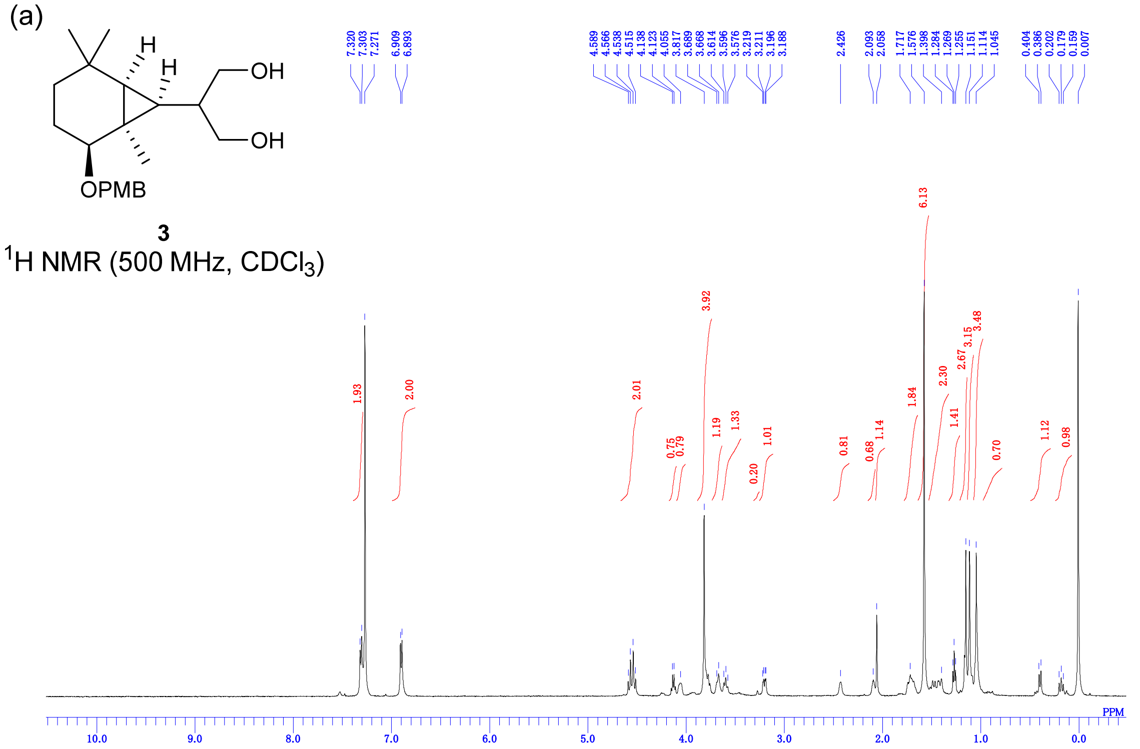

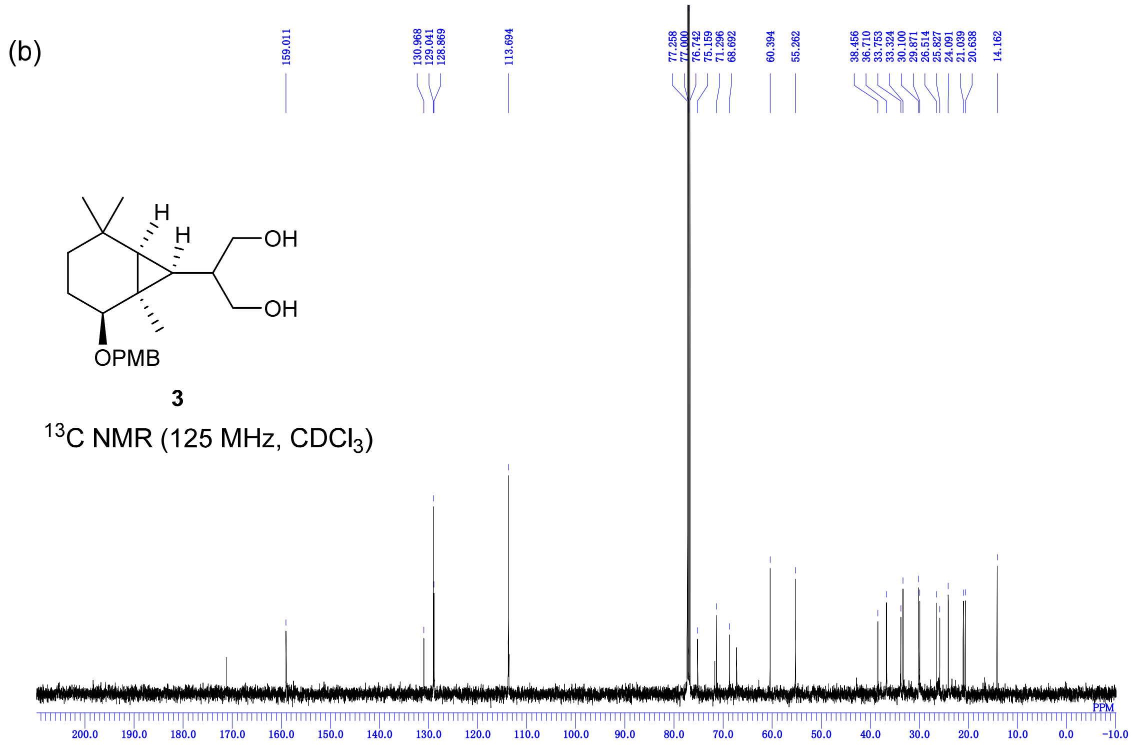

Supplementary Fig. 25. ^{1}H (a) and ^{13}C NMR (b) spectra of 3

(a)

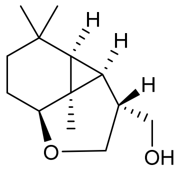

18

^{1}H NMR (500 MHz, $CDCl_3$)

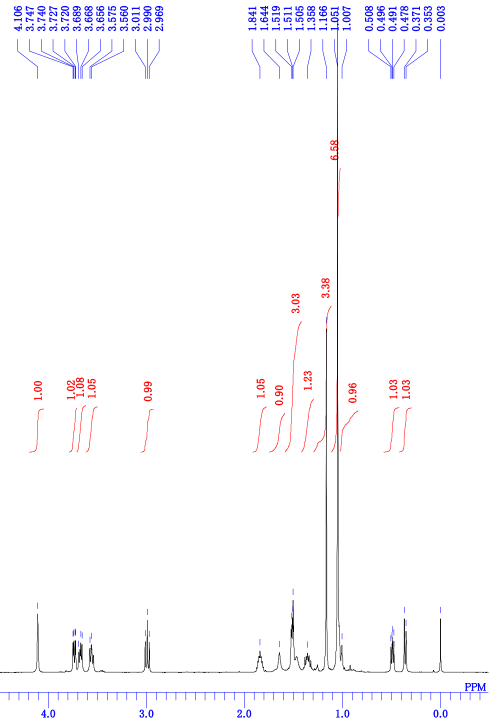

(b)

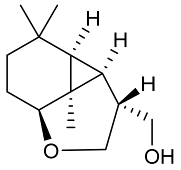

18

^{13}C NMR (125 MHz, $CDCl_3$)

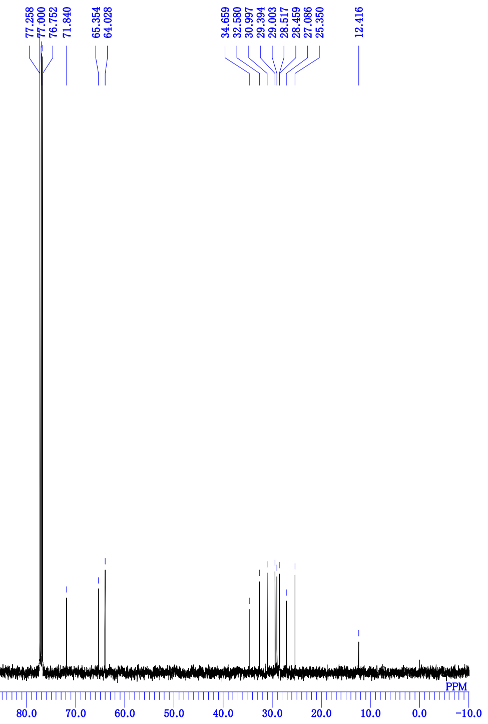

Supplementary Fig. 26. ^{1}H (a) and ^{13}C NMR (b) spectra of 18

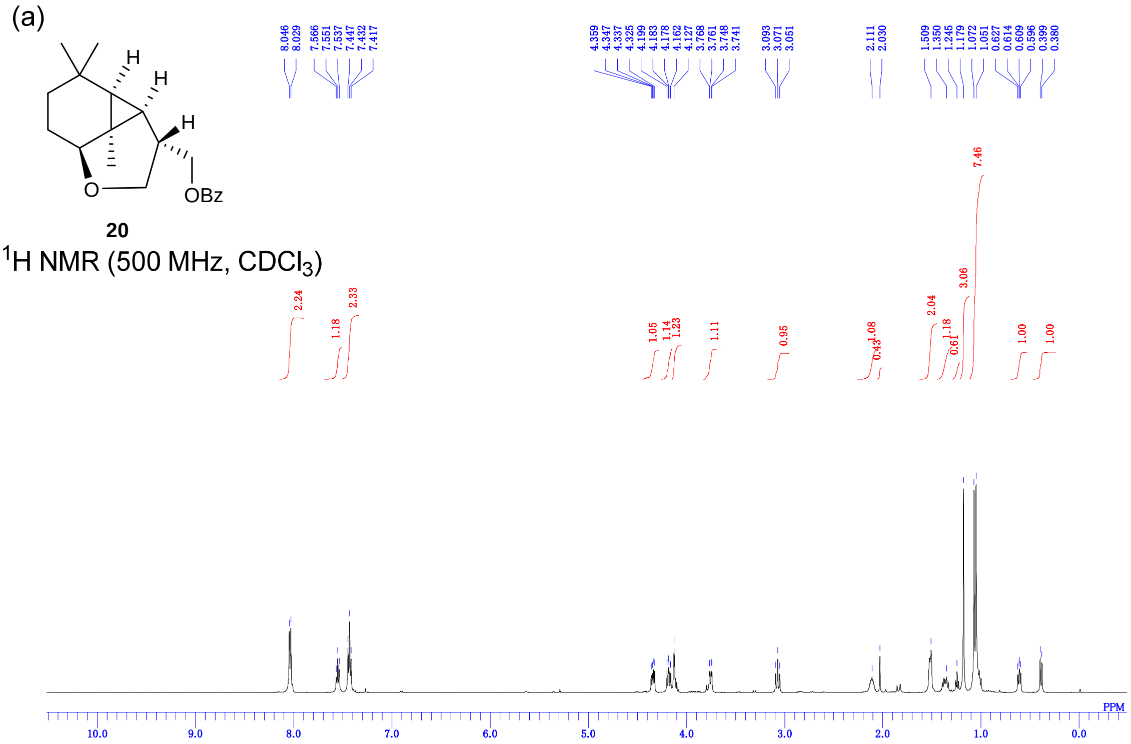

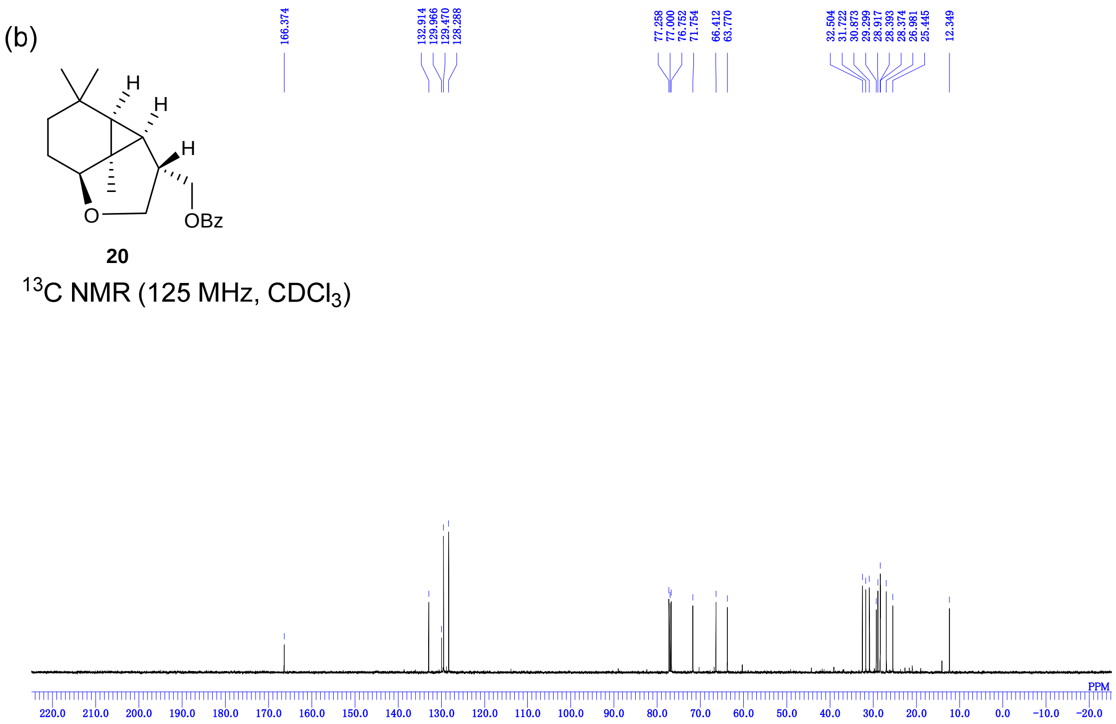

Supplementary Fig. 27. ^{1}H (a) and ^{13}C NMR (b) spectra of 20

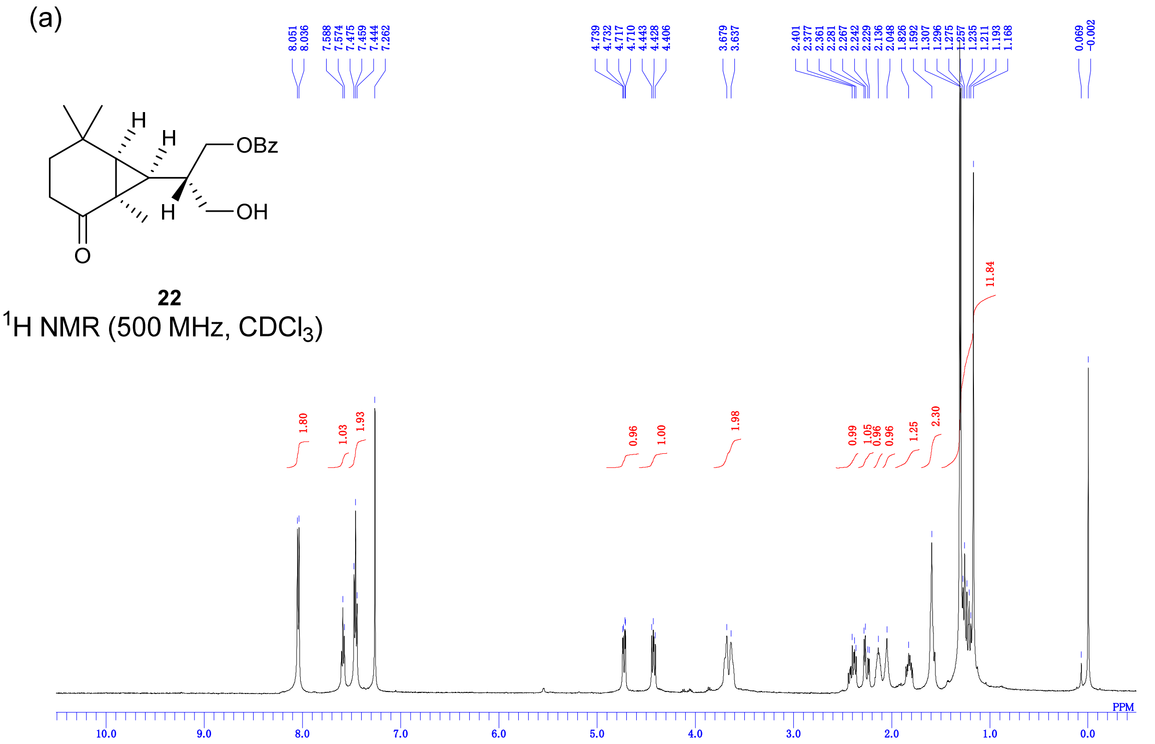

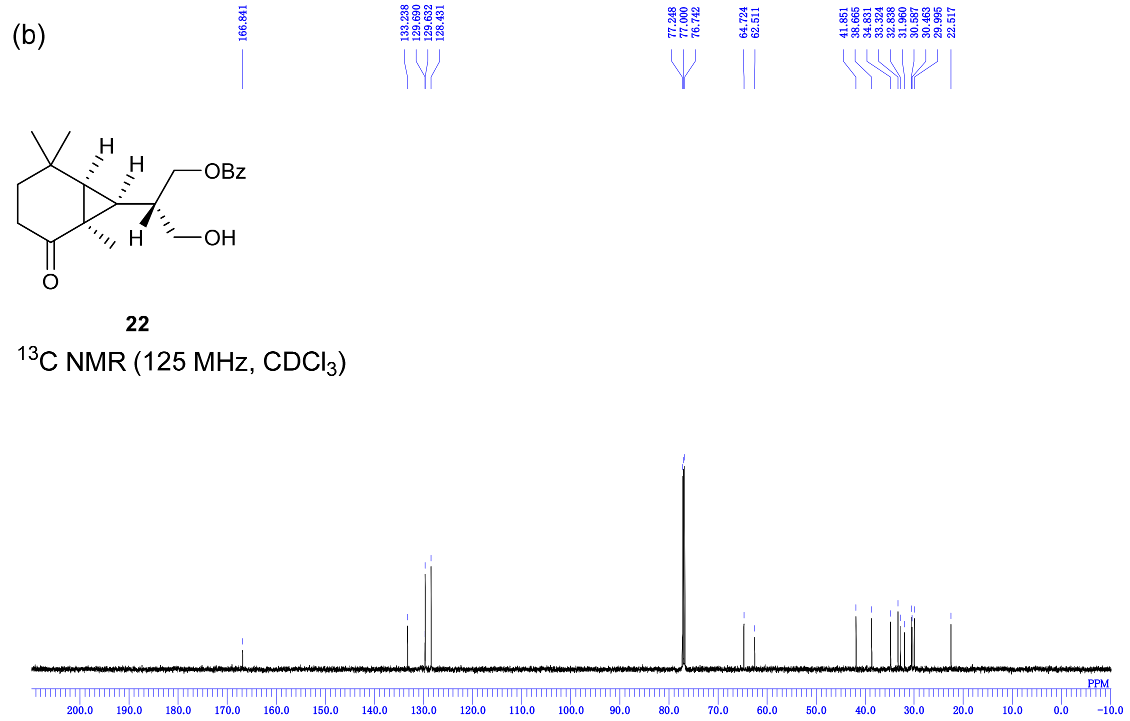

Supplementary Fig. 28. ^{1}H (a) and ^{13}C NMR (b) spectra of 22

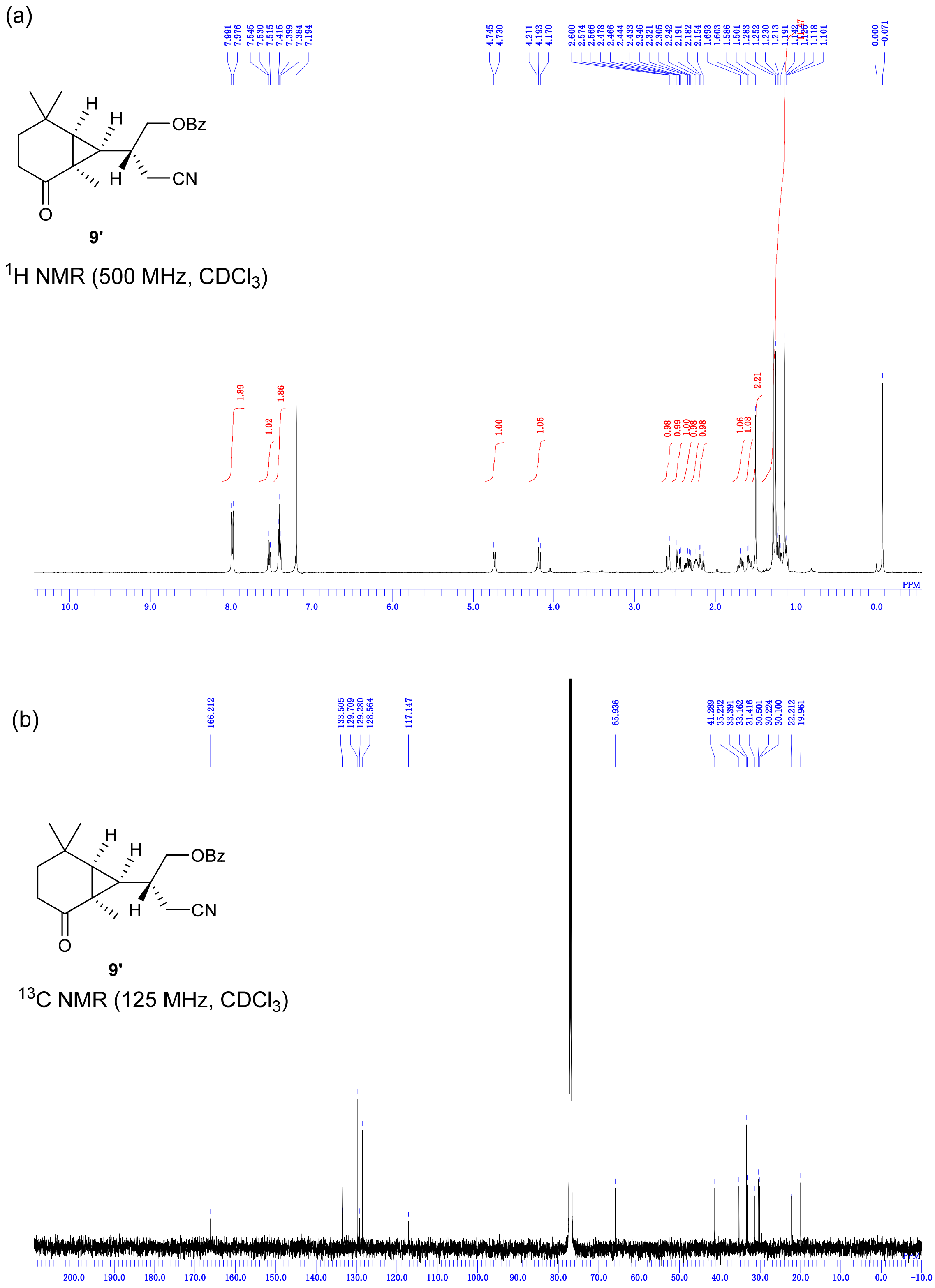

Supplementary Fig. 29. ^{1}H (a) and ^{13}C NMR (b) spectra of 9’

(a)

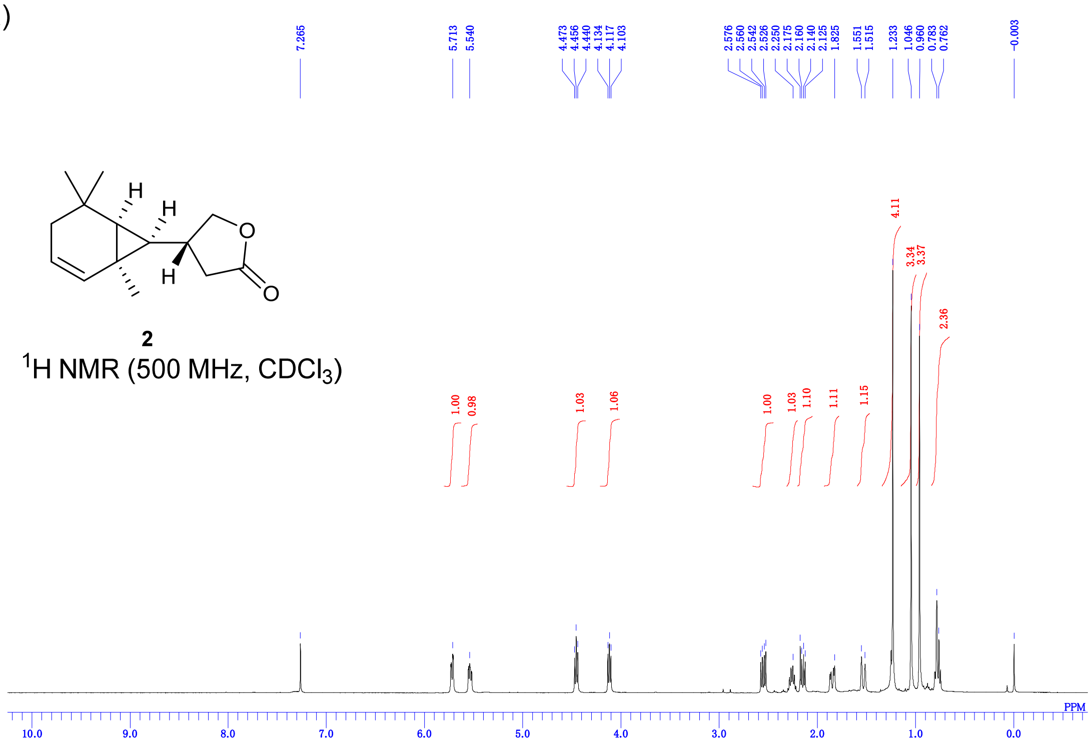

(b)

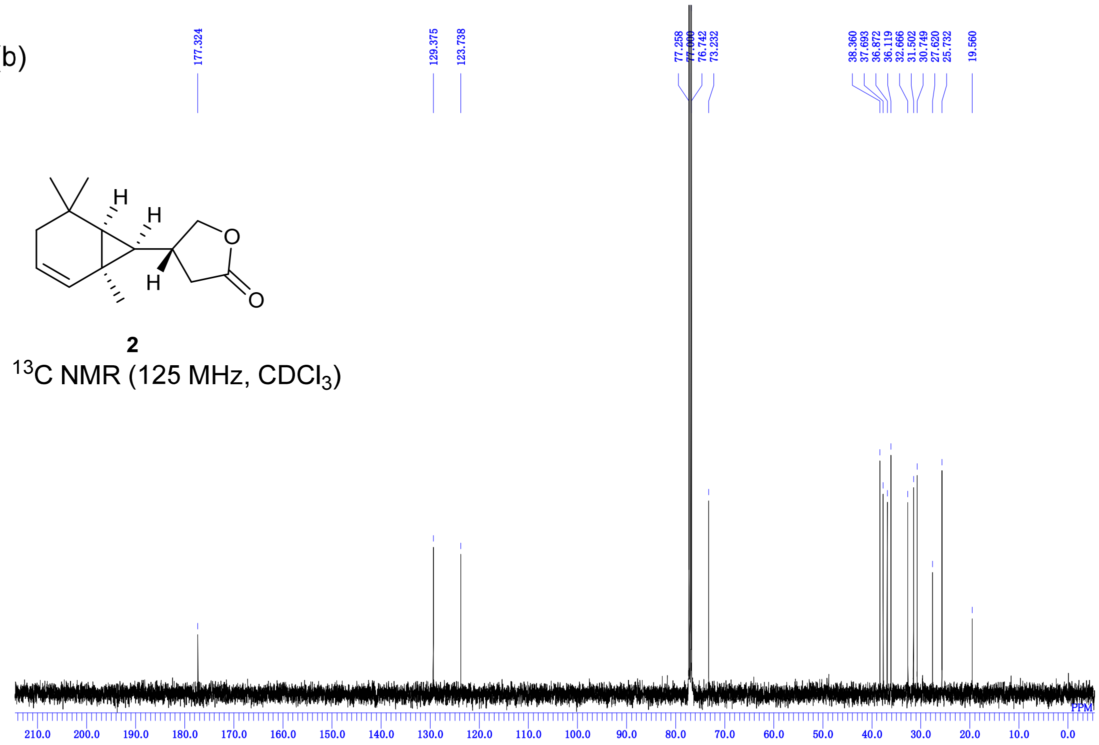

Supplementary Fig. 30. ^{1}H (a) and ^{13}C NMR (b) spectra of 2

(a)

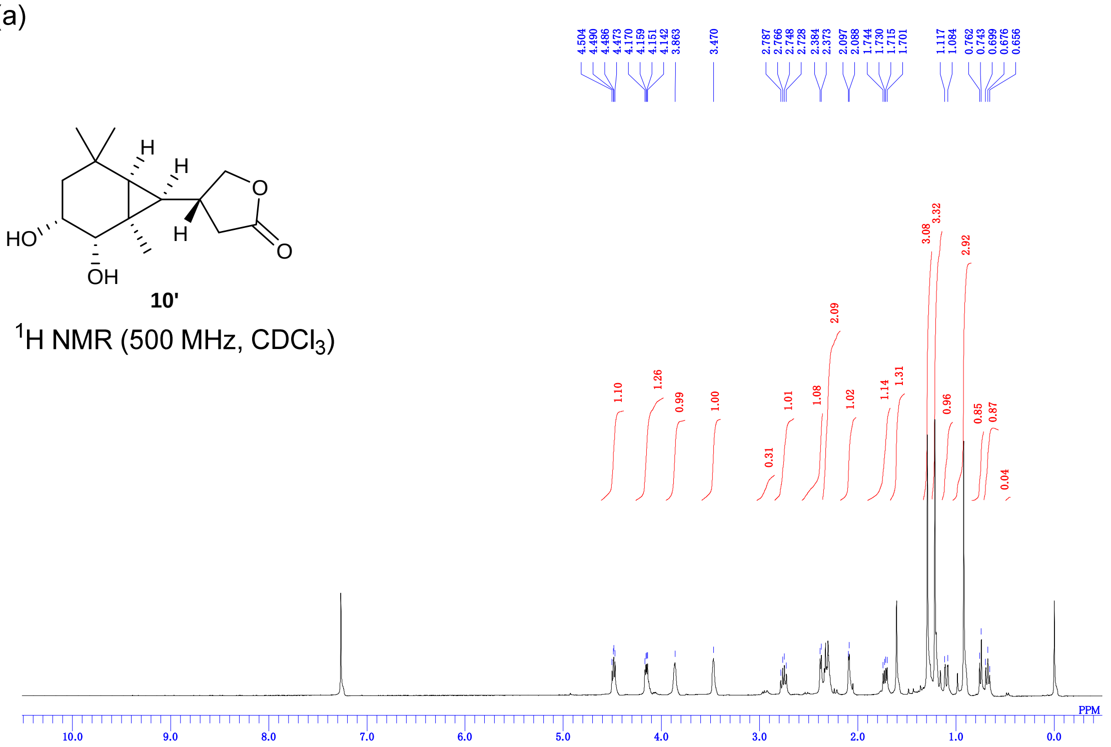

(b)

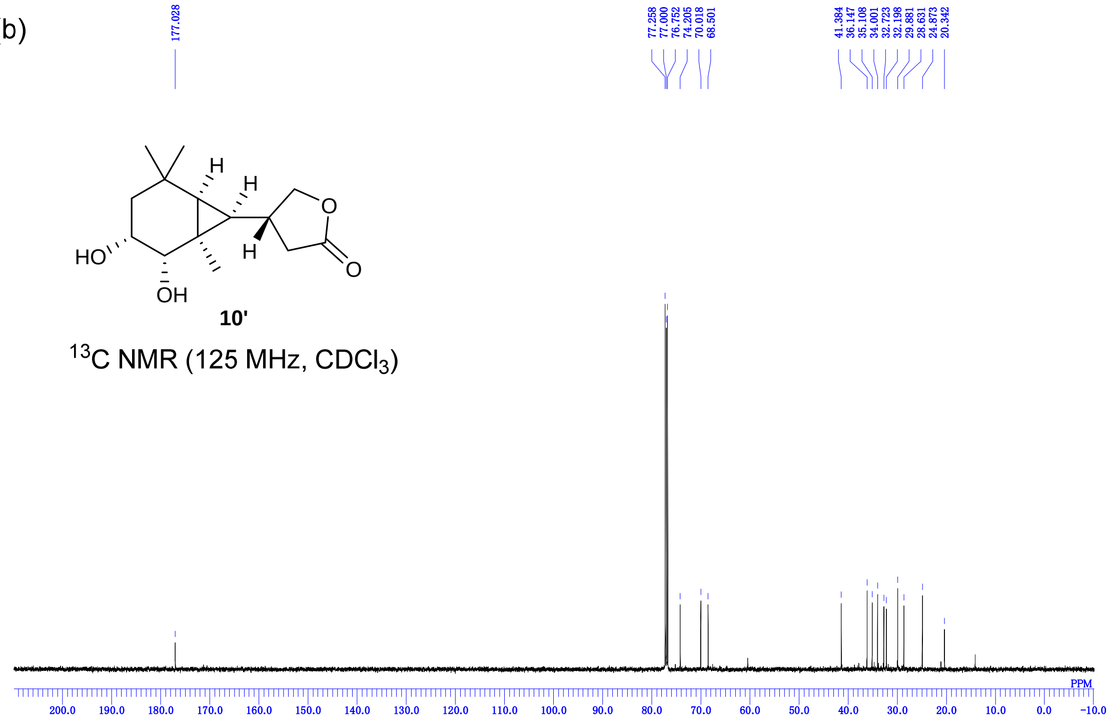

Supplementary Fig. 31. ^{1}H (a) and ^{13}C NMR (b) spectra of 10’

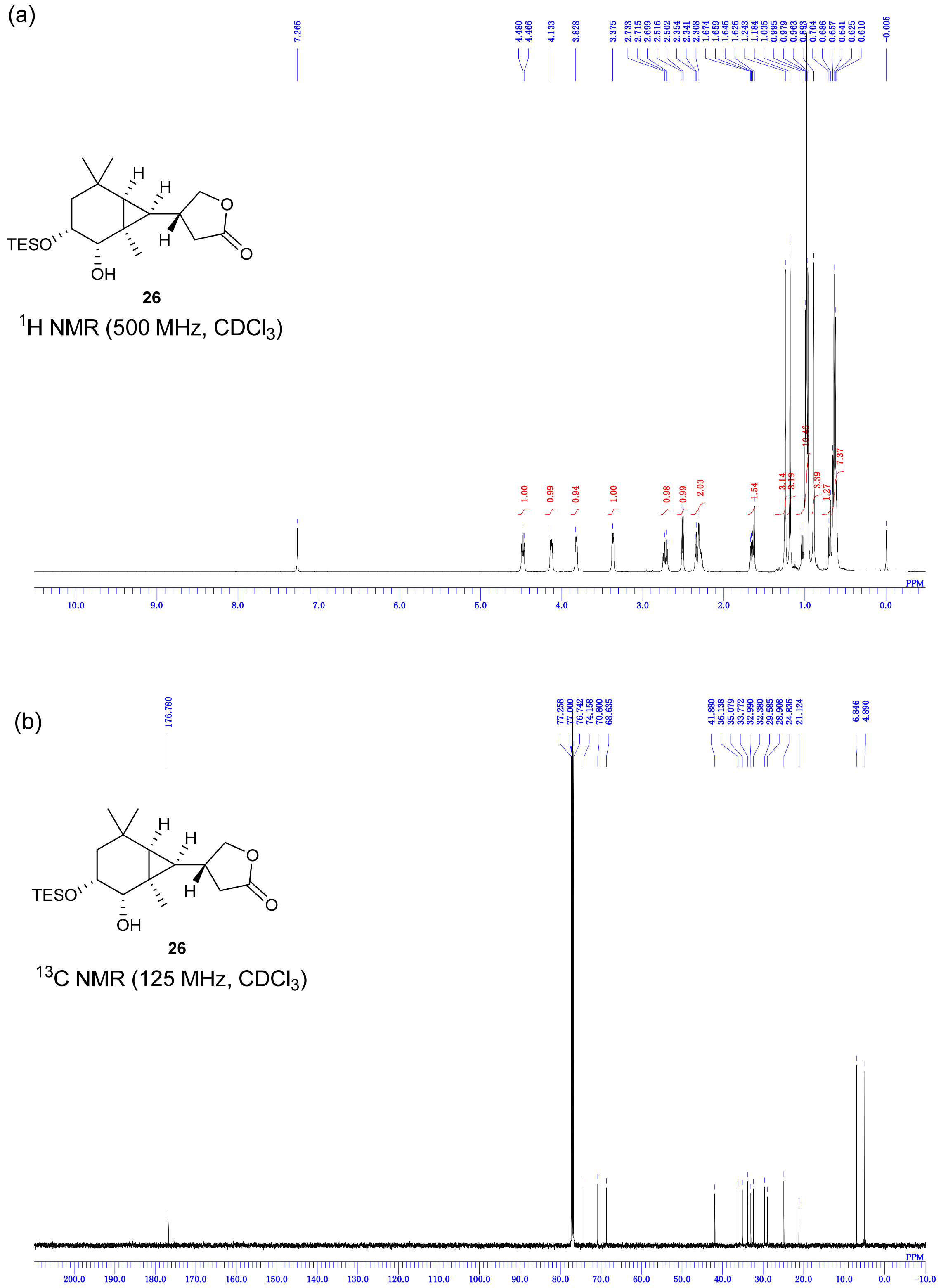

Supplementary Fig. 32. ^{1}H (a) and ^{13}C NMR (b) spectra of 26

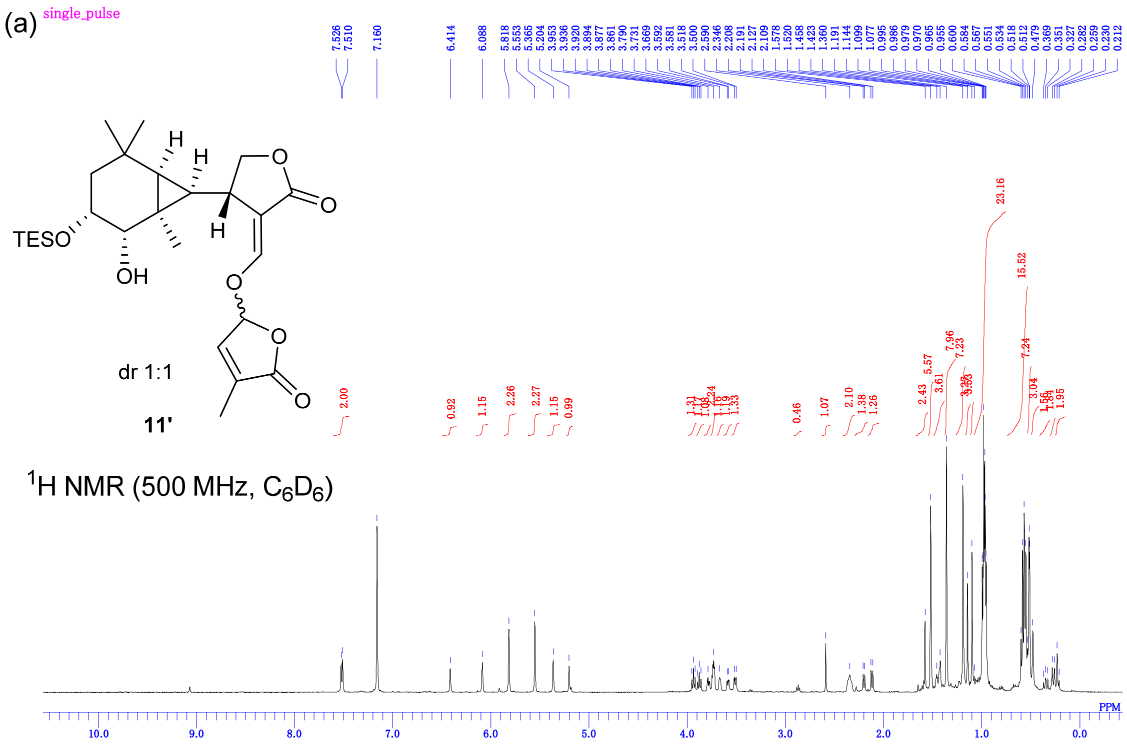

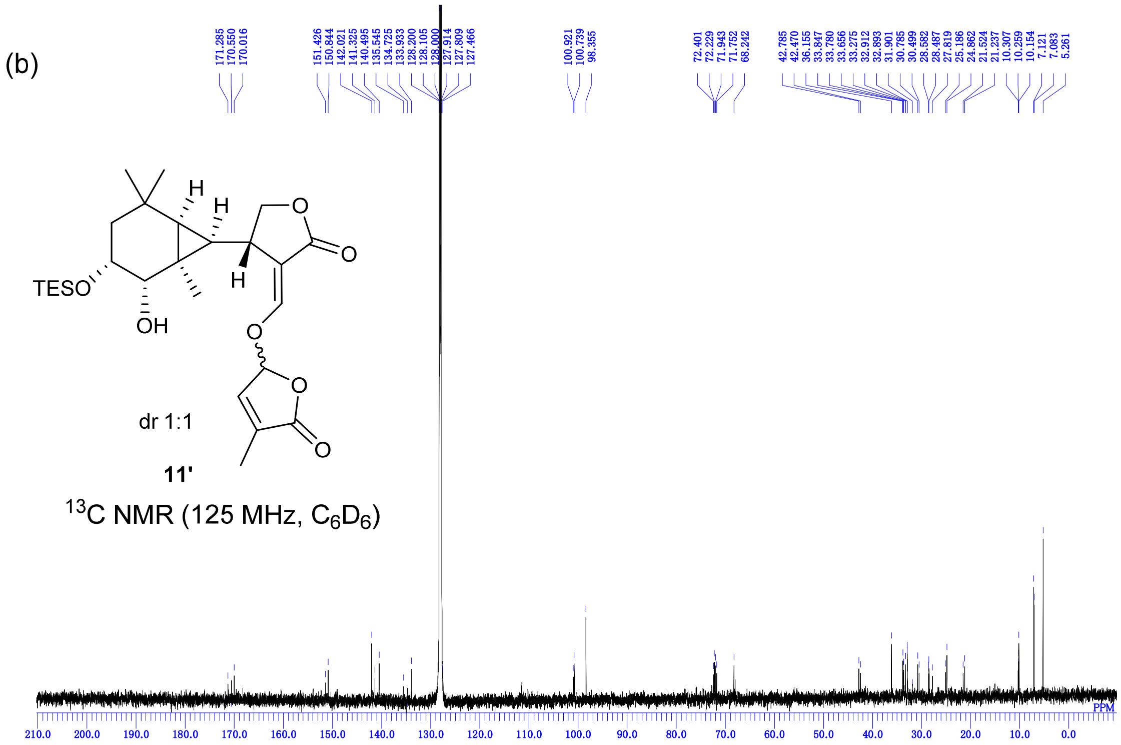

Supplementary Fig. 33. ^{1}H (a) and ^{13}C NMR (b) spectra of 11’

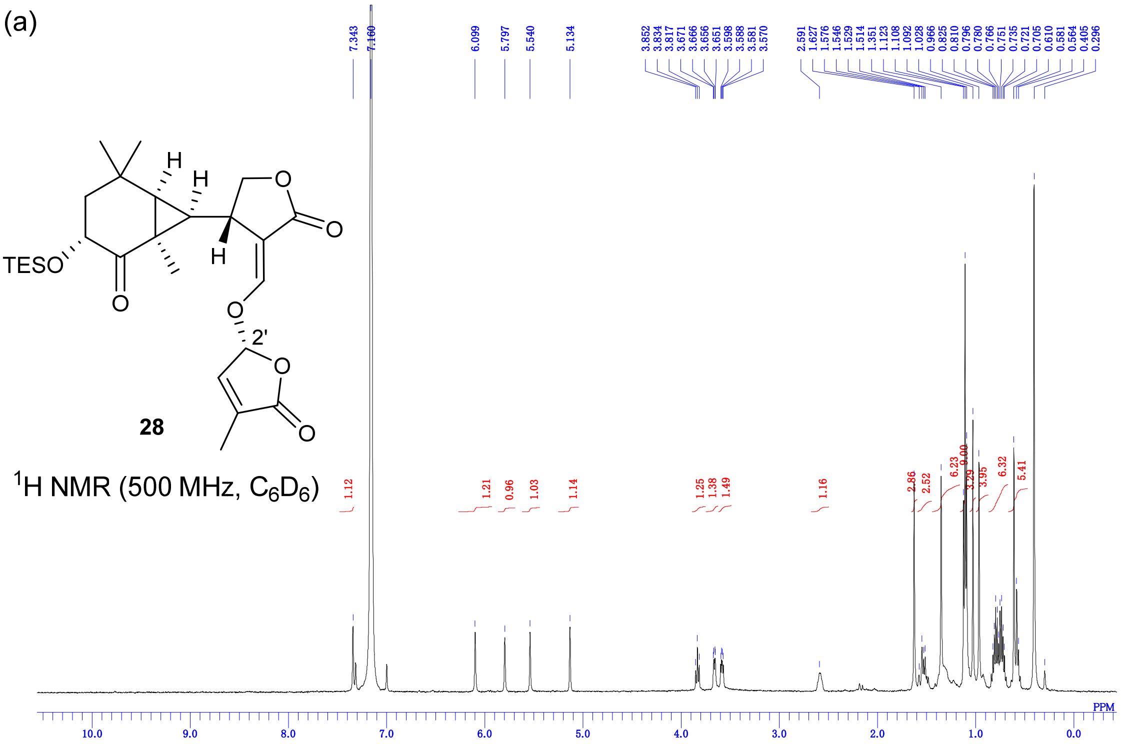

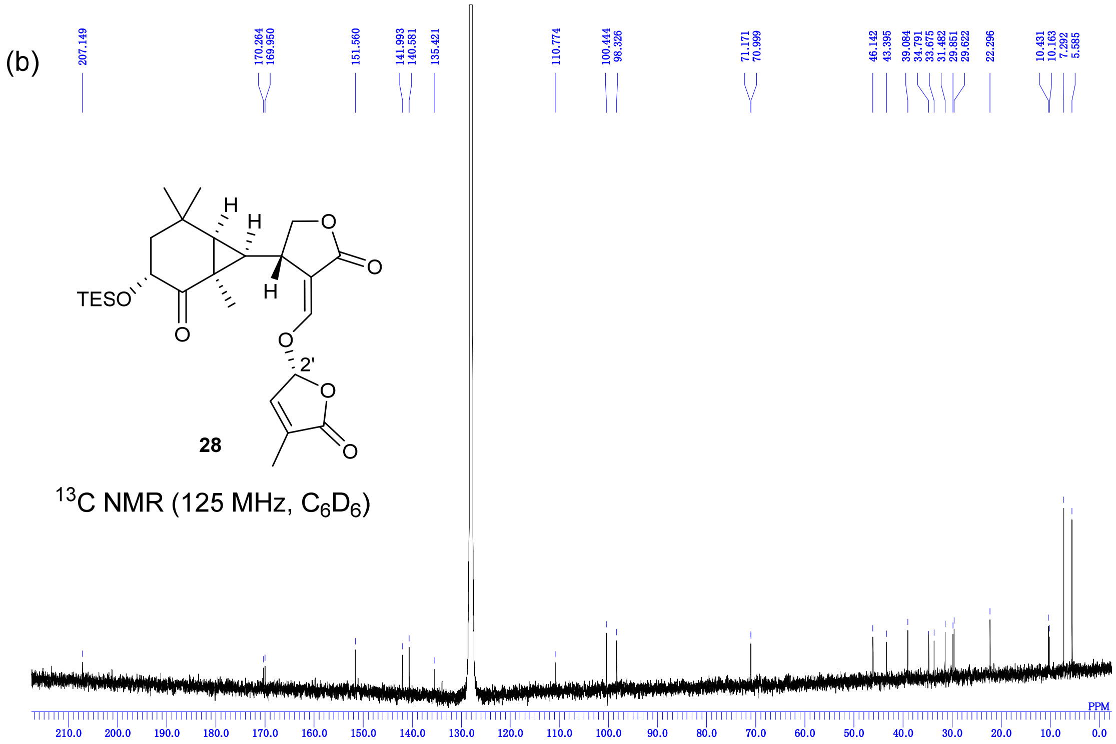

Supplementary Fig. 34. ^{1}**H (a) and** ^{13}**C NMR (b) spectra of 28**

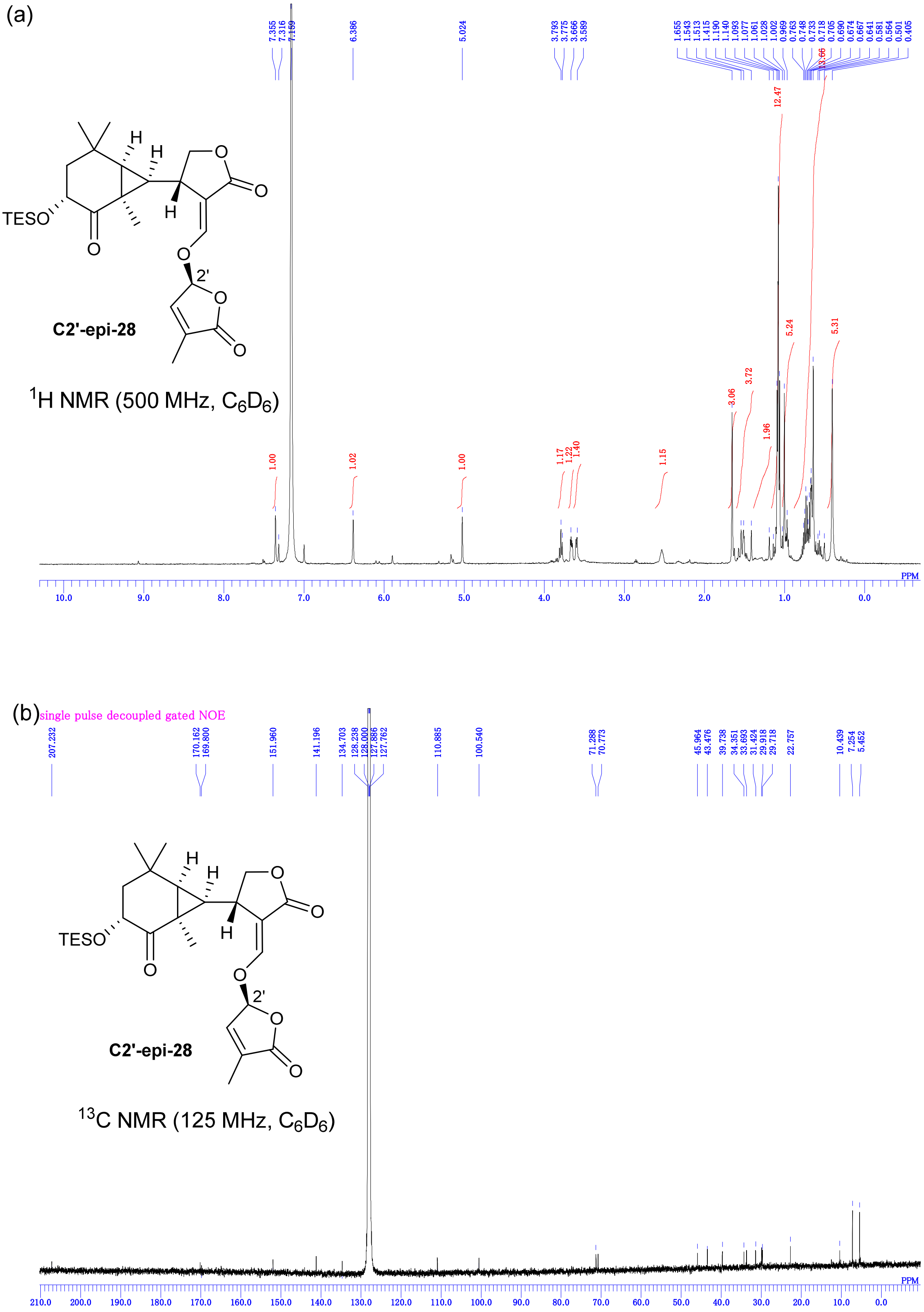

Supplementary Fig. 35. ^{1}H **(a) and** ^{13}C **NMR (b) spectra of C2'-epi-28**

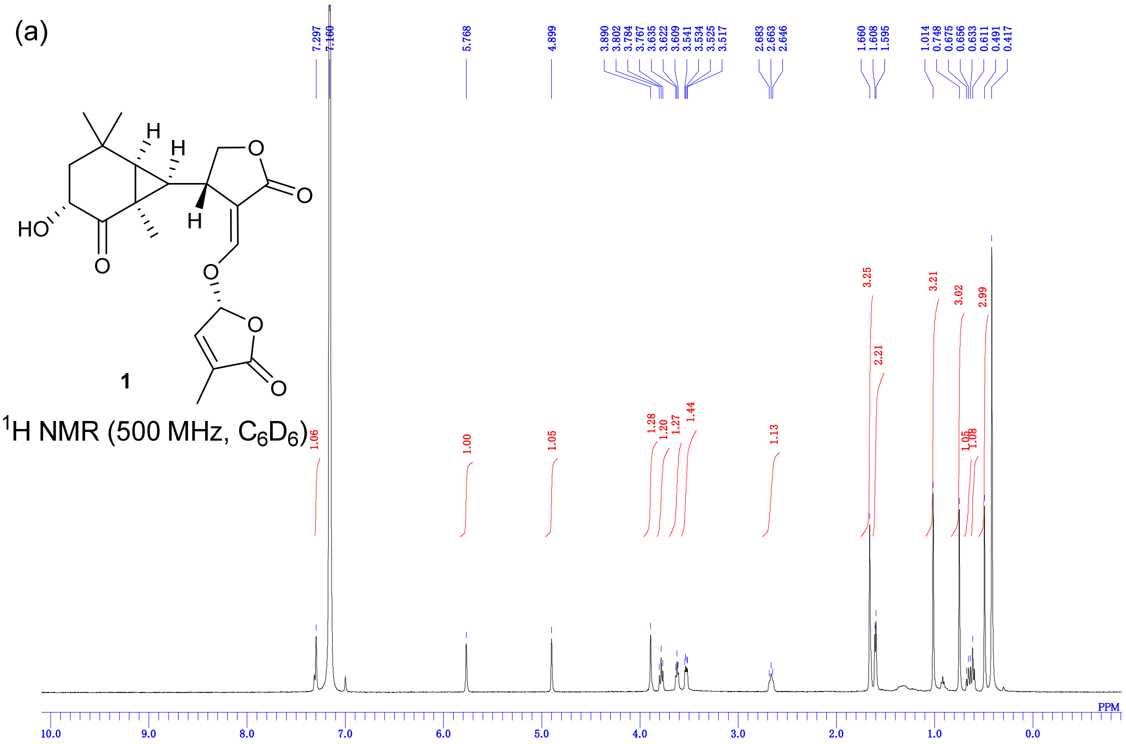

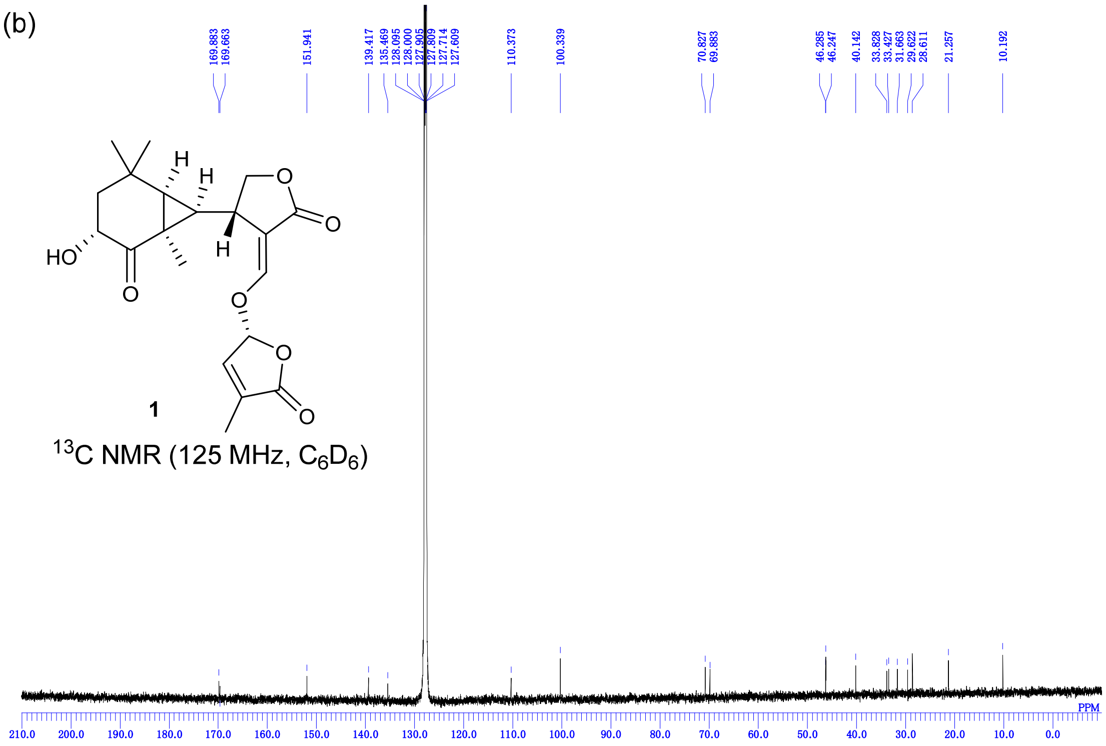

Supplementary Fig. 36. ^{1}H (a) and ^{13}C NMR (b) spectra of 1

(a)

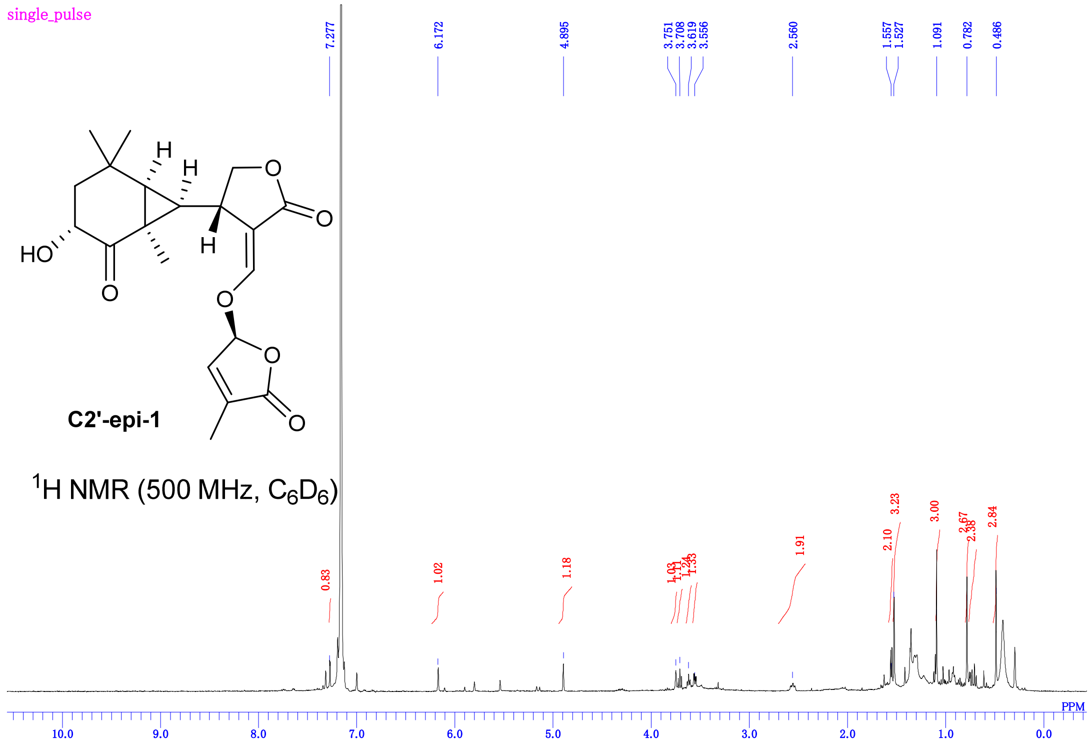

(b)

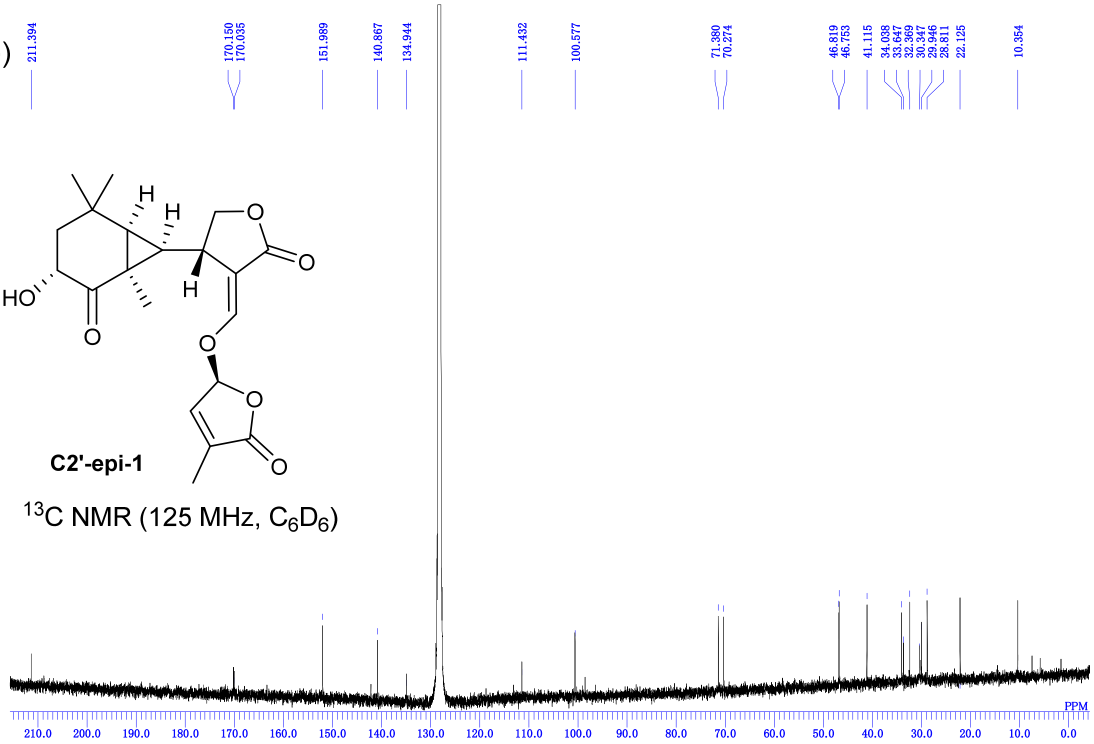

Supplementary Fig. 37. ^{1}H (a) and ^{13}C NMR (b) spectra of C2'-epi-1

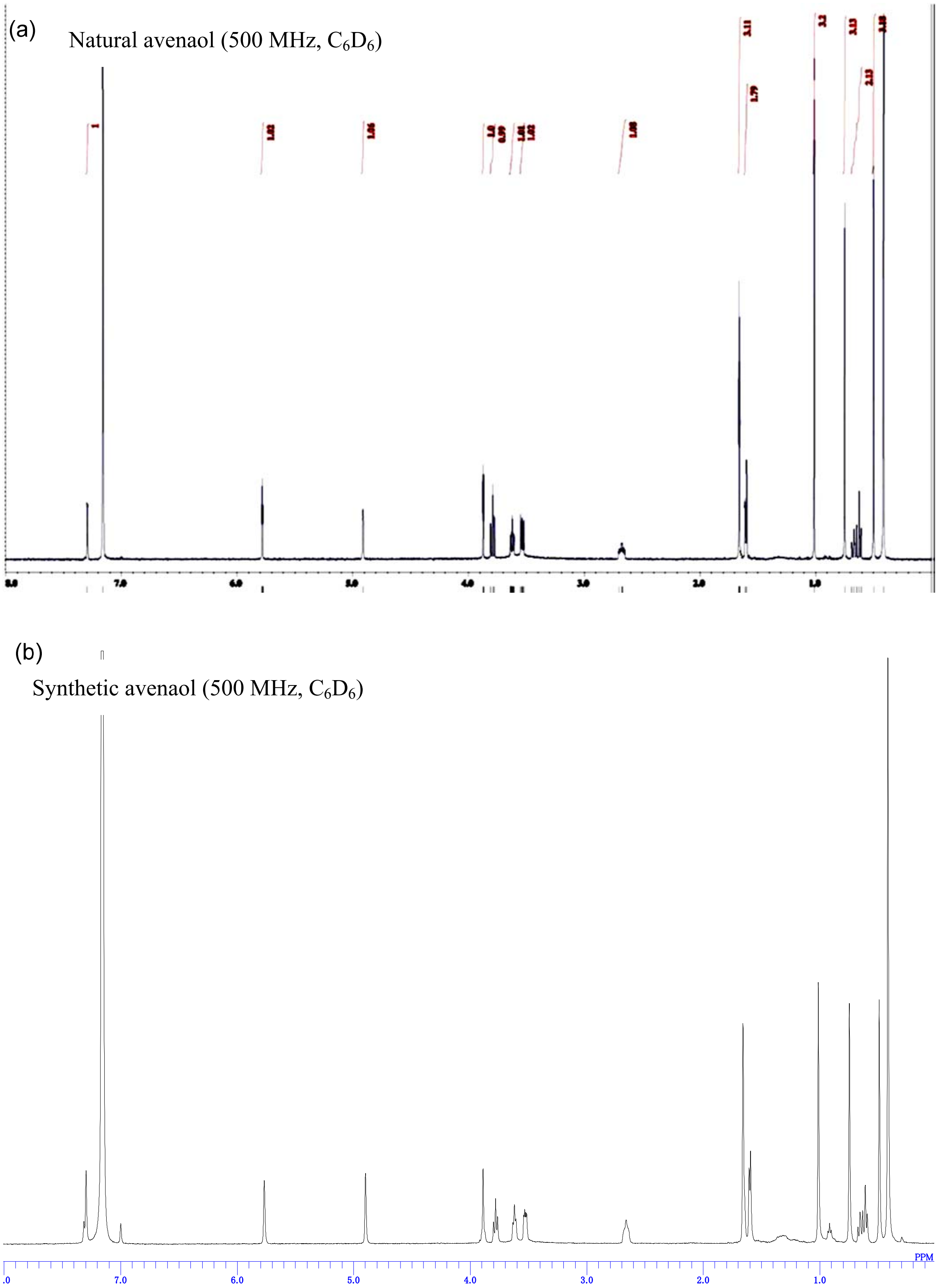

Supplementary Fig. 38. Comparison of ^{1}H NMR spectra of the natural (a) and synthetic avenaol (b).

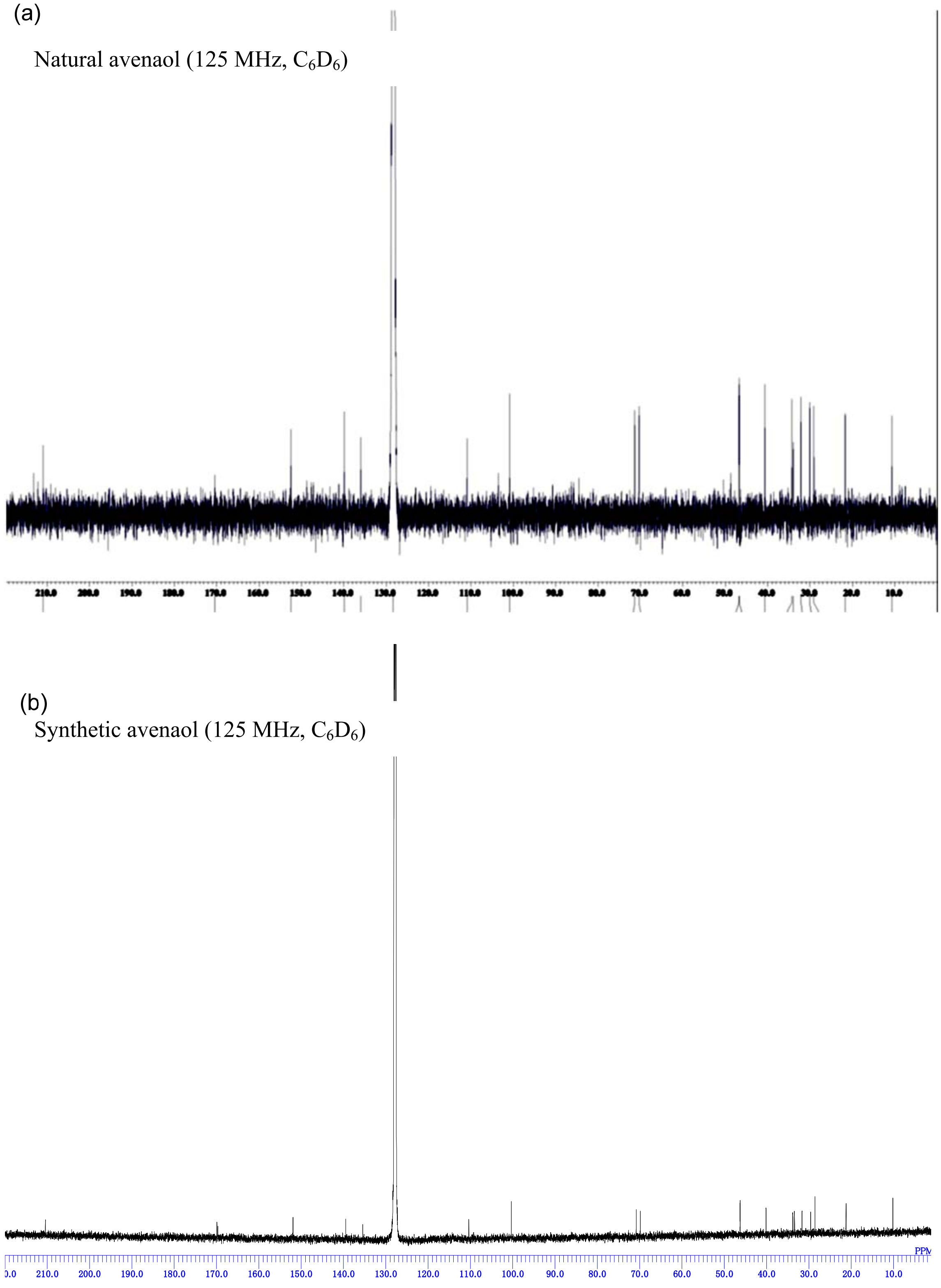

Supplementary Fig. 39. Comparison of ^{13}C NMR spectra of the natural (a) and synthetic avenaol (b).
